# Unique progerin C-terminal peptide ameliorates Hutchinson–Gilford progeria syndrome phenotype by rescuing BUBR1

**DOI:** 10.1038/s43587-023-00361-w

**Published:** 2023-02-02

**Authors:** Na Zhang, Qianying Hu, Tingting Sui, Lu Fu, Xinglin Zhang, Yu Wang, Xiaojuan Zhu, Baiqu Huang, Jun Lu, Zhanjun Li, Yu Zhang

**Affiliations:** 1 Key Laboratory of Molecular Epigenetics of Ministry of Education (MOE), Northeast Normal University, Changchun, China; 2 Key Laboratory of Zoonosis Research, Ministry of Education, College of Animal Science, Jilin University, Changchun, China; 3 Institute of Genetics and Cytology, Northeast Normal University, Changchun, China

**Keywords:** Senescence, Mechanisms of disease, Cell therapies, Ageing

## Abstract

An accumulating body of evidence indicates an association between mitotic defects and the aging process in Hutchinson–Gilford progeria syndrome (HGPS), which is a premature aging disease caused by progerin accumulation. Here, we found that BUBR1, a core component of the spindle assembly checkpoint, was downregulated during HGPS cellular senescence. The remaining BUBR1 was anchored to the nuclear membrane by binding with the C terminus of progerin, thus further limiting the function of BUBR1. Based on this, we established a unique progerin C-terminal peptide (UPCP) that effectively blocked the binding of progerin and BUBR1 and enhanced the expression of BUBR1 by interfering with the interaction between PTBP1 and progerin. Finally, UPCP significantly inhibited HGPS cellular senescence and ameliorated progeroid phenotypes, extending the lifespan of *Lmna*^*G609G/G609G*^ mice. Our findings reveal an essential role for the progerin-PTBP1-BUBR1 axis in HGPS. Therapeutics designed around UPCP may be a beneficial strategy for HGPS treatment.

## Abstract (web summary)

Zhang et al. demonstrate that targeting BUBR1, a nuclear mitotic spindle assembly checkpoint protein, alleviates premature aging phenotypes of progeria in mice via disrupting its nuclear mislocalization induced by progerin interaction.

## Main

HGPS is a rare and severe premature aging disease caused by a de novo point mutation in the *LMNA* gene encoding the intermediate filament proteins lamins A and C, which are structural components of the nuclear lamina^1,2^. The mutant *LMNA* gene (c.1824C>T; p.Gly608Gly) produces a protein called progerin, with 50 amino acids deleted at the carboxyl terminal through alternative splicing^3–6^. The gradual accumulation of progerin causes fibroblast cell senescence in HGPS, which makes the nucleus of the cell both structurally and functionally deficient^7^. Previous studies have shown that progerin is anchored to the nuclear membrane due to the retention of farnesylation modifications, which form insoluble aggregates in the cytoplasm, causing mitotic defects such as abnormal chromosome separation and binuclear and aneuploidy during mitosis in HeLa-transfected progerin and HGPS cells^8,9^. In addition, progerin causes defects in chromosome segregation as early as metaphase, delays nuclear envelope reformation, and traps lamina components and inner nuclear envelope proteins in the endoplasmic reticulum at the end of mitosis^10,11^. Other studies have shown that progerin accumulates with mitotic defects in normal physiologically aging fibroblasts, HGPS cells and Hela-progerin cells^8^. It has been suggested that progerin also causes similar mitotic defects during normal aging. This accumulation of progerin-dependent defects with each round of mitosis predisposes cells to premature senescence. However, the mechanism of how progerin causes mitotic defects in HGPS cells is unclear.

Accurate chromosome segregation is critical to the preservation of euploidy during mitosis in eukaryotic cells. Errors in the molecular mechanisms regulating segregation result in aneuploidy—a hallmark of spontaneous abortions, birth defects and most cancers^12^. In eukaryotes, the spindle assembly checkpoint (SAC) is a ubiquitous safety device that ensures the fidelity of chromosome segregation in mitosis. The core components of the SAC include mainly MAD1, MAD2, BUB1, BUB3, BUBR1 and CDC20, which prevent chromosome missegregation and aneuploidy, and its dysfunction is implicated in tumorigenesis and premature aging^13–15^. BUBR1 is encoded by *BUB1B* and is a component of the SAC that has emerged as a key regulator of aging and longevity in mice^15–17^. A decline in the BUBR1 level occurs with natural aging and induces progeroid features in mice and humans with mosaic variegated aneuploidy (MVA) syndrome^17,18^. *BubR1*^*H/H*^ (hypomorphic BubR1 models) mice are normal in appearance and size at birth but undergo slow postnatal growth and have a shortened lifespan, exhibiting premature aging phenotypes^16^. MVA patients have various progeroid traits, including a shortened lifespan, short stature, variable developmental delay, facial dysmorphisms and cataracts^19–21^. Together, these studies indicate that BUBR1 regulates mitosis and is an important contributor to normal aging. However, whether BUBR1 is involved in the development of HGPS has not been reported.

At present, clinical drugs for HGPS are very rare except for Zokinvy (lonafarnib), which has been approved by the United States (US) Food and Drug Administration (FDA). Zokinvy (lonafarnib) is accompanied by adverse reactions such as diarrhea and vomiting, and these factors limit the effect of farnesyltransferase inhibitors as a means of treatment^22^. Recently, some gene therapies for HGPS treatment have been developed. The clustered regularly interspaced short palindromic repeat–adeno-associated virus 9 system is used to reduce the expression of progerin and the adenine base editor–adeno-associated virus 9 system is used to correct the pathogenic HGPS mutation. In addition, antisense peptide-conjugated phosphorodiamidate morpholino oligomers (PPMOs) are used to block pathogenic splicing of progerin transcripts. Although the application of gene technologies can significantly improve the aging phenotype of HGPS mice and extend their lifespan, it remains unknown whether the application of gene editing can be used safely and effectively in the human body. Therefore, there is an urgent need to develop new drugs for the treatment of HGPS. Peptides are a promising class of molecules that have played a notable role in medical practice, such as in anti-aging, diabetes, antiviral medicines and other areas^23,24^. Previous studies have shown that the FOXO4-DRI peptide selectively induces the apoptosis of senescent cells and reverses the effects of chemotoxicity and aging in mice^25^. In addition, neuropeptide Y delays the cellular HGPS aging phenotype by stimulating autophagy and decreasing progerin accumulation^26^. Therefore, the development of peptide drugs will provide effective new strategies for HGPS treatment. In this study, we developed a unique progerin C-terminal peptide (UPCP) that blocked the binding of progerin and BUBR1 and upregulated BUBR1 dependent on PTBP1. More importantly, UPCP rescued HGPS cellular senescence and ameliorated progeroid features, extending the lifespan in *Lmna*^*G609G/G609G*^ mice. This indicates that UPCP may be a new drug candidate for the treatment of HGPS.

## Results

### BUBR1 is downregulated in HGPS cells

**Fig. 1:**
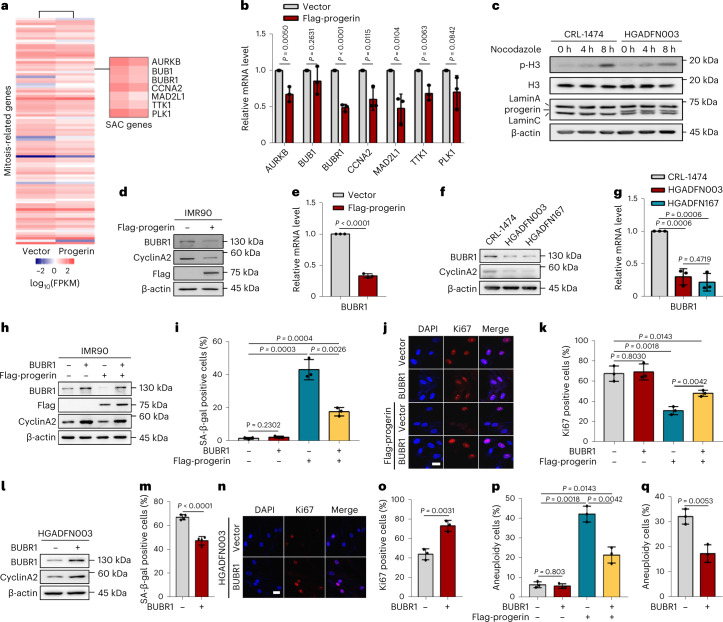
BUBR1 reduction exhibits phenotypes associated with HGPS cellular senescence. **a**, Heatmap showing gene expression of mitosis-related genes in progerin-expressing CRL-1474 cells relative to control cells. **b**, RT-PCR analysis of SAC-related genes in progerin-expressing IMR90 cells relative to control cells; *n* = 3 biologically independent replicates. **c**, Western blot analysis of H3, p-H3, lamin A/C and progerin in CRL-1474 and HGADFN003 cells treated with nocodazole (200 ng ml^–1^) for the indicated number of hours. **d**, Western blot analysis of BUBR1 and CyclinA2 in IMR90 cells with the indicated treatments. **e**, RT-PCR analysis of *BUBR1* in IMR90 cells with the indicated treatments; *n* = 3 biologically independent replicates. **f**, Western blot analysis of BUBR1 and CyclinA2 in CRL-1474 and HGPS cells. **g**, RT-PCR analysis of *BUBR1* in CRL-1474 and HGPS cells; *n* = 3 biologically independent replicates. **h**, Western blot analysis of BUBR1 and CyclinA2 in BUBR1 re-expression IMR90-progerin cells. **i**, Quantification of SA-β-gal positive cells (%) in IMR90 cells with the indicated treatments; *n* = 3 biologically independent replicates. **j**,**k**, Immunofluorescence analysis of Ki67 in IMR90 cells with the indicated treatments. Representative images of Ki67 positive cells (**j**) and quantification of the Ki67 positive cells (%) (**k**); *n* = 3 biologically independent replicates. Scale bars, 20 μm. **l**, Western blot analysis of BUBR1 and CyclinA2 in BUBR1 re-expression HGADFN003 cells. **m**, Quantification of SA-β-gal positive cells (%) in HGADFN003 cells with the indicated treatments (*n* ≥ 200); *n* = 4 biologically independent replicates. **n**,**o**, Immunofluorescence analysis of Ki67 in HGADFN003 cells with the indicated treatments. Representative images of Ki67 positive cells (**n**) and quantification of the Ki67 positive cells (%) (**o**); *n* = 3 biologically independent replicates. Scale bars, 20 μm. **p**, Quantification of aneuploid cells in IMR90 cells with the indicated treatments; *n* = 3 biologically independent replicates. **q**, Quantification of aneuploid cells in HGADFN003 cells with the indicated treatments; *n* = 3 biologically independent replicates. For bar graphs, data are presented as mean ± s.d. Statistical significance was determined in **b**, **e**, **g**, **i**, **k**, **m**, **o**, **p** and **q** using two-tailed unpaired Student’s *t*-test. Source data

**Extended Data Fig. 1:**
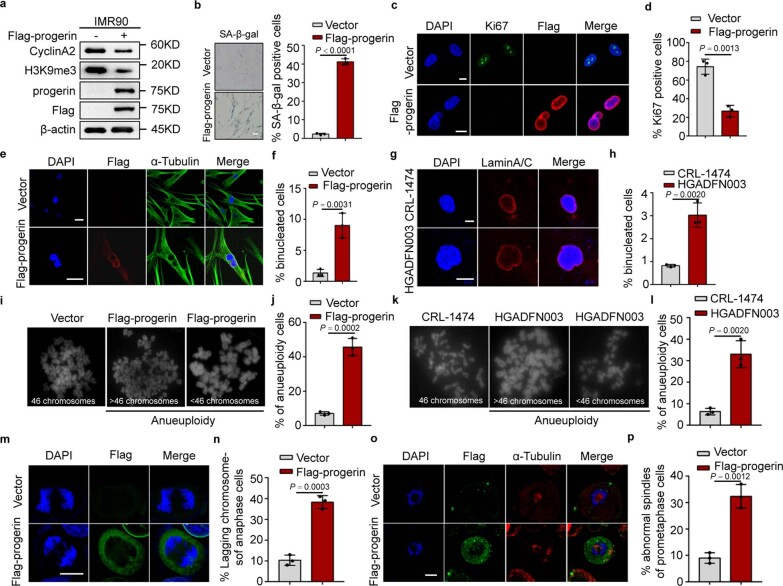
Progerin induces cellular senescence and mitosis defects. a. Western blot analysis of CyclinA2, H3K9me3 and progerin in progerin-expressing IMR90 cells. n = 3 biologically independent replicates. b. β-Gal staining analysis and quantification of SA-β-gal positive cells (%) in progerin-expressing IMR90 cells. n = 3 biologically independent replicates. Scale bars: 100 μm. c,d. Immunofluorescence analysis of Ki67 in progerin-expressing IMR90 cells. Representative images (c) and quantification of the Ki67 positive cells (%) (d). n = 3 biologically independent replicates. Scale bars: 10 μm. e,f. Immunofluorescence analysis of binucleated cells in progerin-expressing IMR90 cells. Representative images (e) and quantification of binucleated cells (%) (f). n = 3 biologically independent replicates. Scale bars: 10 μm. g,h. Immunofluorescence analysis of binucleated cells in CRL-1474 and HGADFN003 cells. Representative images (g) and quantification of binucleated cells (%) (h). n = 3 biologically independent replicates. Scale bars: 30 μm. i,j. Metaphase chromosome spread assay in progerin-expressing IMR90 cells. Representative images (i) and quantification of anueuploid cells (%) (j). n = 3 biologically independent replicates. k,l. Metaphase chromosome spread assay in CRL-1474 and HGADFN003 cells. Representative images (k) and quantification of aneuploid cells (%) (l). n = 3 biologically independent replicates. m,n. Immunofluorescence analysis of chromosome laggings in progerin-expressing H1299 cells. Representative images (m) and quantification of chromosome laggings cells (%) (n). n = 3 biologically independent replicates. Scale bars: 10 μm. o,p. Immunofluorescence analysis of spindles in progerin-expressing H1299 cells. Representative images (o) and quantification of abnormal spindles cells (%) (p). n = 3 biologically independent replicates. Scale bars: 10 μm. For bar and line graphs, data are presented as mean ± SD. Statistical significance was determined in b, d, f, h, j, l, n and p using two-tailed unpaired Student’s t-test. Source data

**Extended Data Fig. 2:**
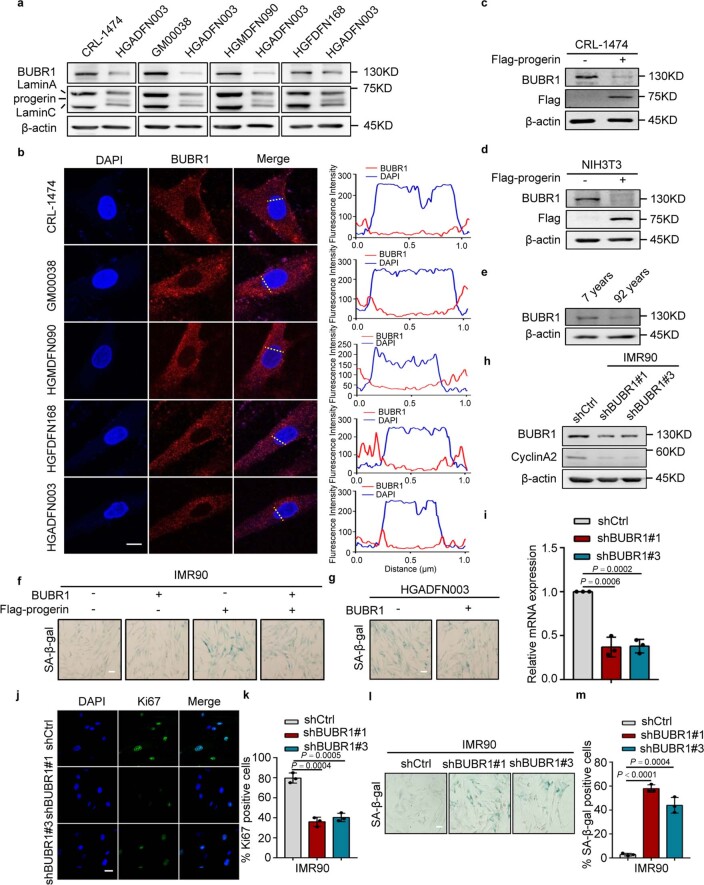
Insufficience of BUBR1 promotes cellular senescence. a. Western blot analysis of BUBR1 in CRL-1474, GM00038, HGMDFN090, HGFDFN168 and HGADFN003 cells. n = 3 biologically independent replicates. b. Immunofluorescence analysis of BUBR1 in control cells (CRL-1474, GM00038, HGMDFN090, HGFDFN168) and HGPS cells (HGADFN003). Representative images of BUBR1 were showed (b, left). Line graphs indicating IF signal intensity across the dotted lines in (b, right). n = 3 biologically independent replicates. Scale bars: 10 μm. c. Western blot analysis of BUBR1 in CRL-1474 cells infected with Flag-progerin. n = 3 biologically independent replicates. d. Western blot analysis of BubR1 in NIH-3T3 cells infected with Flag-progerin. n = 3 biologically independent replicates. e. Western blot analysis of BUBR1 in human skin fibroblasts from 7-year-old and 92-year-old individual. n = 3 biologically independent replicates. f. Representative images of SA-β-Gal positive cells in IMR90 cells with the indicated treatments. Scale bars: 100 μm. g. Representative images of SA-β-Gal positive cells in HGADFN003 cells with the indicated treatments. Scale bars: 100 μm. h. Western blot analysis of BUBR1 and CyclinA2 in IMR90 cells infected with shRNA (shCtrl, shBUBR1#1 or #3). i. RT-PCR analysis of BUBR1 in IMR90 cells with the indicated treatments. n = 3 biologically independent replicates. j,k. Immunofluorescence analysis of Ki67 in IMR90 cells with the indicated treatments. Representative images (j) and quantification of the Ki67 positive cells (%) (k). n = 3 biologically independent replicates. Scale bars: 20 μm. l,m. β-Gal staining analysis in IMR90 cells with the indicated treatments. Representative images (l) and quantification of SA-β-gal positive cells (%) (m). n = 3 biologically independent replicates. Scale bars: 100 μm. For bar and line graphs, data are presented as mean ± SD. Statistical significance was determined in i, k and m using two-tailed unpaired Student’s t-test. Source data

To explore the mechanism of how progerin induces cellular defects and premature aging, human fibroblasts IMR90 were used as an HGPS model via viral infection with progerin. Expression of progerin resulted in mitotic defects (Extended Data Fig. 1). To discover how progerin induced mitotic defects in premature aging, we conducted RNA-sequencing analysis to explore which mitosis-related genes were regulated by progerin. This analysis identified 94 differentially expressed mitosis-related genes (Fig. 1a). Among them, genes related to SAC such as *AURKB*, *BUB1*, *BUBR1*, *MAD2L1*, *TTK1* and *PLK1* were identified. The data showed that *AURKB*, *BUBR1*, *MAD2L1*, *TTK1* and *CCNA2* were downregulated in IMR90 cells with progerin expression (IMR90-progerin) (Fig. 1b). Decreased SAC activity was also observed, as evidenced by reduced phosphohistone H3 in HGPS fibroblasts, which indicated that progerin inhibited the expression of SAC-related genes and impaired the SAC signal response in premature aging (Fig. 1c). Prominently, BUBR1 was reduced in IMR90-progerin cells and HGPS patient cells (Fig. 1d–g and Extended Data Fig. 2a,b), which has not been reported in HGPS. Consistently, the same phenomenon was observed in CRL-1474 and NIH-3T3 fibroblasts transfected with progerin (Extended Data Fig. 2c,d). Meanwhile, BUBR1 was decreased in physiological fibroblasts from a 92-year-old individual (Extended Data Fig. 2e). Therefore, we discovered that BUBR1 was downregulated in HGPS cells.

To study the role of BUBR1 in regulating HGPS cellular senescence, we overexpressed BUBR1 in HGPS cells. BUBR1 significantly antagonized HGPS cellular senescence, including the increased level of CyclinA2 (Fig. 1h), the reduced activity of SA-β-gal (Fig. 1i and Extended Data Fig. 2f) and the increased Ki67 positive staining in IMR90-progerin cells (Fig. 1j,k). Similarly, BUBR1 also restored HGPS fibroblast senescence (Fig. 1l–o and Extended Data Fig. 2g). Mitotic defects were also reduced markedly by the overexpression of BUBR1 in IMR90-progerin cells and HGPS cells, accompanied by a decreased number of aneuploid cells (Fig. 1p,q). Moreover, depletion of BUBR1 significantly induced IMR90 fibroblast senescence (Extended Data Fig. 2h–m). Taken together, these data indicate that BUBR1 antagonizes progerin-mediated HGPS cellular senescence, which means that BUBR1 plays an important role in HGPS.

### BUBR1 mislocalization by interaction with progerin

**Fig. 2:**
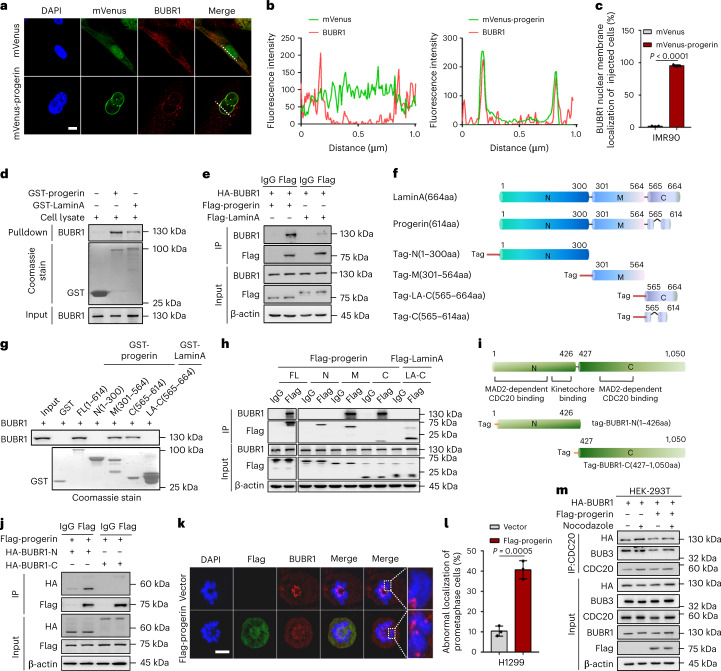
BUBR1 is anchored to the nuclear membrane and forms complexes with progerin. **a**, Immunofluorescence analysis of BUBR1 in IMR90 cells with the indicated treatments. Scale bar, 10 μm. **b**, Line graphs indicating immunofluorescence signal intensity across the dotted lines in **a**. **c**, Quantification of the BUBR1 nuclear membrane localization (%) in IMR90 cells with the indicated treatments; *n* = 3 biologically independent replicates. **d**, GST pulldown assay of progerin or lamin A and BUBR1, GST-progerin or GST-lamin A, which were incubated with HEK-293T cell lysate. **e**, CoIP of progerin or lamin A and BUBR1 in HEK-293T cells. HEK-293T cells were cotransfected with Flag-progerin or Flag-lamin A and HA-BUBR1. Progerin or lamin A was immunoprecipitated (IP) with anti-Flag antibody. **f**, Diagram of the domains in progerin and lamin A. **g**, GST pulldown assay of progerin or lamin A truncated mutants and BUBR1. Different truncated mutants of progerin or lamin A were incubated with HEK-293T cell lysate. **h**, CoIP of different truncated mutants of progerin or lamin A and BUBR1 in HEK-293T cells. HEK-293T cells were transfected with different truncated mutants of progerin or lamin A, which were IP with the anti-Flag antibody. **i**, Diagram of the domains in BUBR1. **j**, CoIP of progerin and different truncated mutants of BUBR1 in HEK-293T cells. HEK-293T cells were cotransfected with Flag-progerin and HA-BUBR1-N truncated mutant (aa residues 1–426) or HA-BUBR1-C (truncated mutant aa residues 427–1,050). Progerin was IP with the anti-Flag antibody. **k**,**l**, Immunofluorescence analysis of BUBR1 in H1299 cells infected with Flag-progerin (**k**) and quantification of cells with abnormal localization of BUBR1 (%) (**l**); *n* = 3 biologically independent replicates. Scale bar, 10 μm. **m**, CoIP of CDC20 and BUBR1 or BUB3 in HEK-293T cells. HEK-293T cells were coinfected with the HA-BUBR1 and/or Flag-progerin for 48 h and treated with nocodazole for 8 h. CDC20 was IP with the anti-CDC20 antibody. For bar graphs, data are presented as mean ± s.d. Statistical significance was determined in **c** and **l** using two-tailed unpaired Student’s *t*-test. IgG, immunoglobulin G. Source data

**Extended Data Fig. 3:**
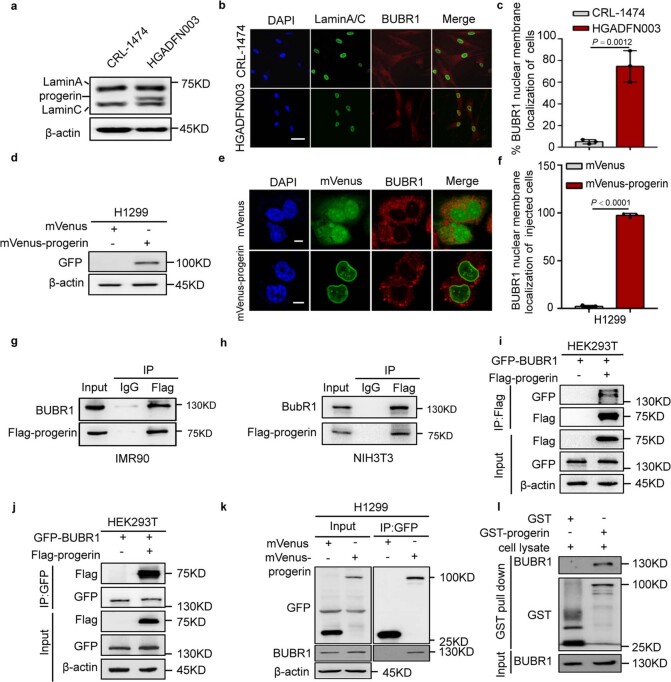
BUBR1 interacts with progerin and they co-locate on the nuclear membrane. a. Western blot analysis of Lamin A/C and progerin in CRL-1474 and HGADFN003 cells. n = 3 biologically independent replicates. b. Immunofluorescence analysis of Lamin A/C and BUBR1 in CRL-1474 and HGADFN003 cells, Scale bars: 50 μm. c. Quantification of the BUBR1 nuclear membrane localization in CRL-1474 and HGADFN003 cells. n = 3 biologically independent replicates. d. Western blot analysis of mVenus-progerin in H1299 cells with the indicated treatments. mVenus tag, a variant form of GFP tag could be detected by anti-GFP antibody. n = 3 biologically independent replicates. e. Immunofluorescence analysis of mVenus-progerin and BUBR1 in H1299 cells with the indicated treatments, Scale bars: 10 μm. f. Quantification of the BUBR1 nuclear membrane localization in H1299 cells with the indicated treatments. n = 3 biologically independent replicates. g,h. Co-IP of progerin and BUBR1 in IMR90 cells or NIH-3T3 cells-transfected with Flag-progerin, the progerin band pulled down by IP with the Flag-antibody. n = 3 biologically independent replicates. i,j. Co-IP of progerin and BUBR1 in HEK-293T cells. HEK-293T cells were co-transfected with Flag-progerin and GFP-BUBR1. Progerin or BUBR1 was IP from HEK-293T cells lysate with the anti-Flag or anti-GFP antibody. n = 3 biologically independent replicates. k. Co-IP assay of progerin and BUBR1 in H1299 cells. H1299 cells were transfected with mVenus or mVenus-progerin. Progerin was IP from H1299 cells lysate with the anti-GFP antibody. n = 3 biologically independent replicates. l. GST pulldown assay of progerin and BUBR1. GST or GST-progerin was incubated with HEK-293T cells lysate. n = 3 biologically independent replicates. For bar and line graphs, data are presented as mean ± SD. Statistical significance was determined in c and f using two-tailed unpaired Student’s t-test. Source data

Previous studies have shown that BUBR1 is located in the cytoplasm in interphase cells and in the kinetochore in prometaphase cells^27^. However, we discovered that not only was the level of BUBR1 reduced during HGPS cellular senescence, but parts of the remaining BUBR1 were also anchored to the nuclear membrane and colocalized with progerin in interphase HGPS cells, implying that progerin and BUBR1 form a complex in HGPS cells (Fig. 2a–c, Extended Data Figs. 2b and 3). To verify whether BUBR1 mislocalization may be caused by a differential interaction of BUBR1 with progerin compared with lamin A, we detected the interaction of progerin–BUBR1 using glutathione S-transferase (GST) pulldown and coimmunoprecipitation (CoIP) assays. The interaction of BUBR1 with progerin was enhanced strongly compared with lamin A (Fig. 2d,e).

To map the progerin/lamin A domain involved in BUBR1 binding, we created a series of truncations of progerin and lamin A^28^: the GST/Flag-tagged progerin N-terminal region (progerin-N; amino acid (aa) residues 1–300), the progerin middle region (progerin-M; aa residues 301–564) and the progerin C-terminal region (progerin-C; aa residues 565–614) or the lamin A C-terminal region (lamin A-C; aa residues 565–664) (Fig. 2f). We found that although the middle region of progerin/lamin A could bind to BUBR1, the C-terminal region of progerin, but not the C-terminal region of lamin A, bound to BUBR1, which explained the different affinities of progerin–BUBR1 and lamin A–BUBR1 (Fig. 2g,h). Next, we found that the N-terminal region of BUBR1 (BUBR1-N; aa residues 1–426) was critical for the binding of BUBR1 with the kinetochore or CDC20 (another component of the SAC, could bind to BUBR1 during the SAC signal response) bound with progerin (Fig. 2i,j). The combination caused abnormal kinetochore localization of BUBR1 in H1299 cells (Fig. 2k,l) and interfered in the binding of BUBR1-CDC20 treated with or without nocodazole (a microtubule-disrupting agent, which could activate the SAC signal response) (Fig. 2m). Collectively, these data demonstrate that progerin mediated BUBR1 mislocalization and interfered in the binding between BUBR1 and other SAC proteins.

### UPCP blocks progerin–BUBR1 binding and upregulates BUBR1 abundance

**Fig. 3:**
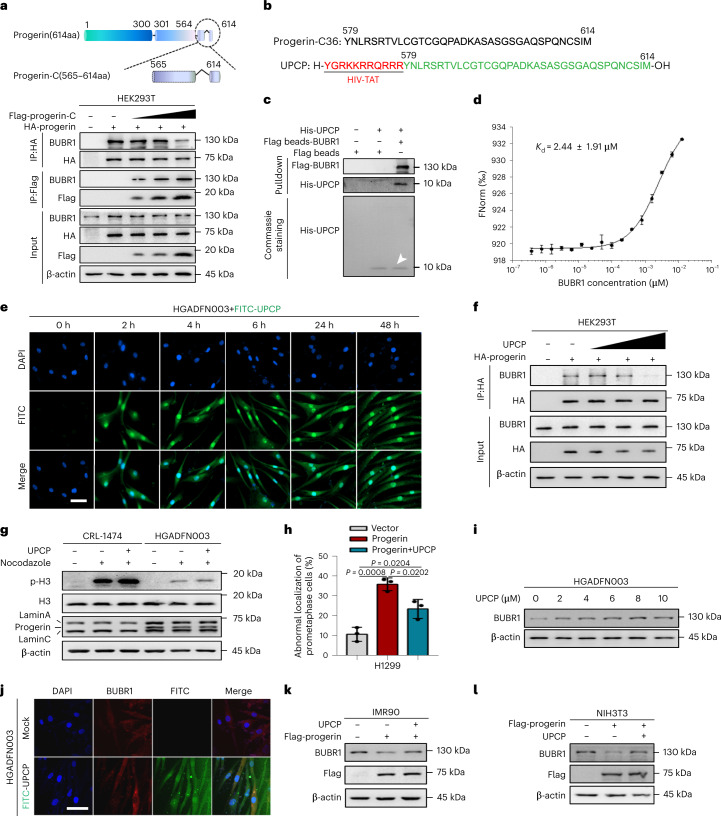
UPCP prevents BUBR1 from interacting with progerin and modulates BUBR1 abundance. **a**, Top panel shows diagram of the domains in progerin or progerin-C (aa residues 565–614). CoIP of progerin or progerin-C and BUBR1 in HEK-293T cells. HEK-293T cells were cotransfected with increasing amounts of Flag-progerin-C (5 μg, 10 μg and 15 μg) and HA-progerin. Progerin-C or Progerin was IP with the anti-Flag or anti-HA antibody; *n* = 3 biologically independent replicates. **b**, Sequence of progerin-C36 (aa residues 579–614) used for the design of UPCP. Red letters indicate HIV-TAT peptide. **c**, Pulldown assay of the interaction between UPCP and BUBR1. Purified His-tagged UPCP was incubated with the Flag beads, Flag beads-BUBR1 (BUBR1 coupled to Flag beads). Total amounts of His-UPCP were visualized by Coomassie brilliant blue staining. The white arrow represents the band of His-UPCP. **d**, MST assay of the interaction between UPCP and BUBR1; *n* = 3 biologically independent replicates. FNorm, normal fluoescence. K_d_, dissociation constant. **e**, Cellular uptake of UPCP in HGPS cells visualized by FITC, Scale bars, 50 μm. **f**, CoIP of progerin and BUBR1 in HEK-293T cells. HEK-293T cells were transfected with Flag-progerin and treated with increasing concentrations of UPCP (6 μM, 12 μM and 18 μM). Progerin was IP with the anti-Flag antibody. **g**, Western blot analysis of H3, p-H3, lamin A/C and progerin in CRL-1474 and HGADFN003 cells treated with nocodazole (200 ng ml^–1^) alone or combination with UPCP. **h**, Quantification of the number of abnormal localization cells of BUBR1 (%) in H1299 cells with the indicated treatments; *n* = 3 biologically independent replicates. **i**, Western blot analysis of BUBR1 in HGADFN003 cells treatment with the increasing concentrations of UPCP (0 μM, 2 μM, 4 μM, 6 μM, 8 μM, 10 μM). **j**, Immunofluorescence analysis of BUBR1 in HGADFN003 cells with indicated treatments, Scale bar, 50 μm. **k**, Western blot analysis of BUBR1 in progerin-expressing IMR90 cells with the indicated treatments. **l**, Western blot analysis of BUBR1 in progerin-expressing NIH-3T3 cells with the indicated treatments. For bar graphs, data are presented as mean ± s.d. Statistical significance was determined in **h** using two-tailed unpaired Student’s *t*-test. Source data

**Extended Data Fig. 4:**
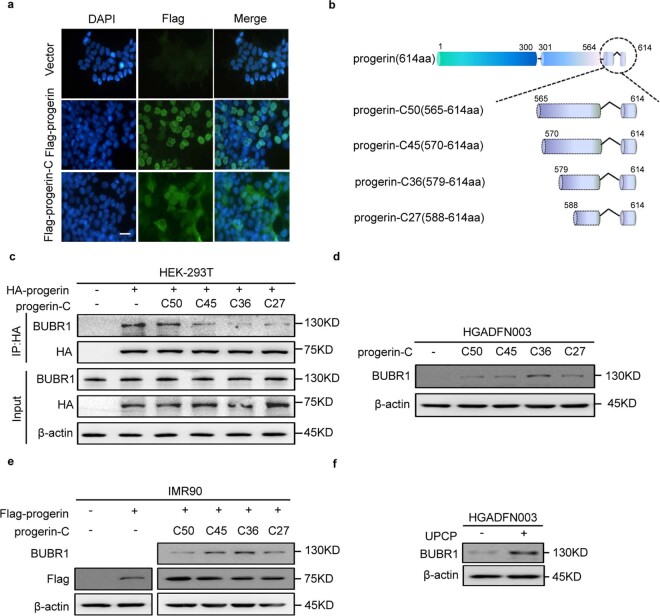
Progeirn-C blocks the interaction between BUBR1 and progerin. a. Immunofluorescence analysis of Flag-progerin-C and Flag-progerin in HEK-293T cells with the indicated treatments. n = 3 biologically independent replicates. Scale bars: 100 μm. b. Diagram of the domains in progerin-C. c. Co-IP assay of progerin and BUBR1 in HEK-293T cells co-transfected with truncated mutants of progerin-C and HA-progerin. Progerin was IP from HEK-293T cells lysate with the anti-HA antibody. n = 3 biologically independent replicates. d. Western blot analysis of BUBR1 in HGADFN003 cells infected with truncated mutants of progerin-C. n = 3 biologically independent replicates. e. Western blot analysis of BUBR1 in IMR90-progerin cells infected with truncated mutants of progerin-C. n = 3 biologically independent replicates. f. Western blot analysis of BUBR1 in HGADFN003 cells treatment with UPCP (6 μM) for 72 h. n = 3 biologically independent replicates. Source data

We have confirmed that progerin tethered BUBR1 to the nuclear membrane (Fig. 2), which prompted us to examine whether BUBR1 could be released from the nuclear membrane by interfering with the interaction of progerin and BUBR1. We showed that progerin-C can bind with BUBR1 specifically (Fig. 2g,h), so we speculated that progerin-C could compete with progerin to bind BUBR1. We first investigated the location of progerin-C (aa residues 565–614), which showed that progerin-C was distributed throughout the cell, unlike progerin, which is distributed in the nuclear envelope (Extended Data Fig. 4a). Moreover, as the expression of progerin-C gradually increased, the interaction between progerin and BUBR1 gradually weakened and the interaction between progerin-C and BUBR1 gradually increased (Fig. 3a). Thus, progerin-C competes with progerin to bind BUBR1 and releases BUBR1 from progerin. To further determine the functional domain of progerin-C, we expressed a series of truncated progerin-C proteins (Extended Data Fig. 4b) and performed interference experiments with progerin and BUBR1. The results showed that progerin-C36 (aa residues 579–614) could block the binding of progerin and BUBR1 (Extended Data Fig. 4c). We therefore designed a cell-permeable UPCP (unique progerin C-terminal peptide, which was designed as a fusion with HIV-TAT) comprising part of the BUBR1-interaction domain of progerin (Fig. 3b). Pulldown and microscale thermophoresis (MST) assays showed that UPCP could combine with BUBR1 directly in vitro (Fig. 3c,d). Moreover, we observed that UPCP, which was modified by fluorescein isothiocyanate (FITC), was taken up by the cells as soon as 2 h after administration (Fig. 3e). Following its uptake, UPCP interfered with the interaction between progerin and BUBR1 (Fig. 3f). Further, UPCP repaired the SAC signal response and correctly localized BUBR1 to the kinetochore (Fig. 3g,h). Unexpectedly, we found that UPCP could also modulate BUBR1 abundance in HGPS cells, IMR90-progerin and NIH-3T3-progerin cells (Fig. 3i–l and Extended Data Fig. 4d–f). Our data demonstrate that UPCP interfered with the interaction of progerin and BUBR1 and upregulated BUBR1.

### UPCP stabilizes *BUBR1* mRNA dependent on PTBP1 in HGPS cells

**Fig. 4:**
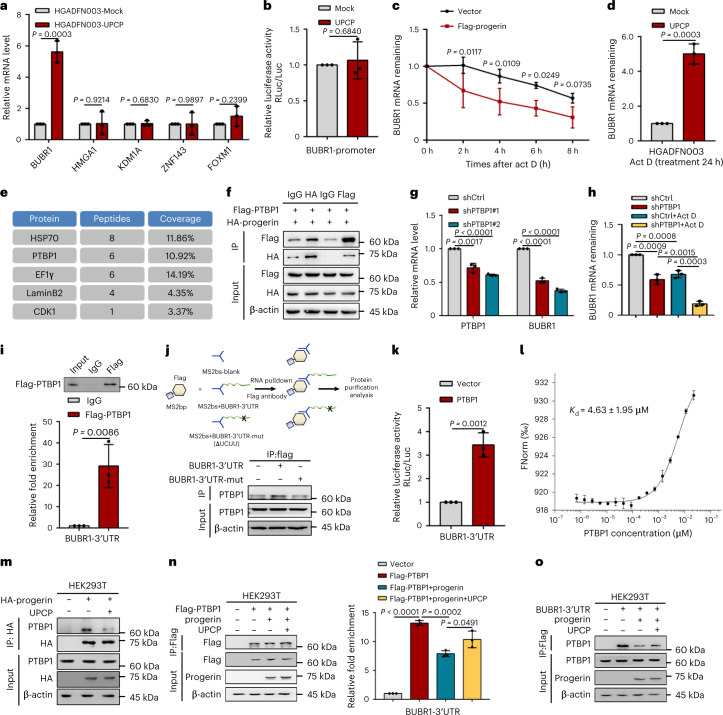
PTBP1 stabilizes *BUBR1* mRNA stability. **a**, RT-PCR analysis of *BUBR1*, *HMGA1*, *KDM1A*, *ZNF143* and *FOXM1* in HGADFN003 cells with the indicated treatments; *n* = 3 biologically independent replicates. **b**, Luciferase assay of *BUBR1* promoter in HEK-293T cells with the indicated treatments; *n* = 3 biologically independent replicates. **c**, mRNA stability assay of *BUBR1* in progerin-expressing IMR90 cells treatment with Actinomycin D (10 μg ml^–1^); *n* = 3 biologically independent replicates. **d**, mRNA stability assay of *BUBR1* in HGADFN003 cells with the indicated treatments; *n* = 3 biologically independent replicates. **e**, HSP70, PTBP1, EF1γ, lamin B2 and CDK1 were identified as progerin-binding proteins by MS. **f**, CoIP of progerin and PTBP1 in HEK-293T cells with the indicated treatments; *n* = 3 biologically independent replicates. **g**, RT-PCR analysis of *PTBP1* and *BUBR1* in IMR90 cells infected with shRNA (shCtrl, shPTBP1#1 or #2); *n* = 3 biologically independent replicates. **h**, mRNA stability assay of *BUBR1* in IMR90 cells with the indicated treatments; *n* = 3 biologically independent replicates. **i**, RIP assay of interaction between PTBP1 and *BUBR1* mRNA 3′-UTR in HEK-293T cells with the indicated treatments; *n* = 3 biologically independent replicates. **j**, Schematic diagram of the MS2-based RNA pulldown strategy (top). RNA pulldown assay of *BUBR1* mRNA 3ʹ-UTR or 3ʹ-UTR-mut (△UCUU) and PTBP1 in HEK-293T cells with the indicated treatments. **k**, Luciferase activity assay of *BUBR1* mRNA 3ʹ-UTR in overexpressing PTBP1 HEK-293T cells; *n* = 3 biologically independent replicates. **l**, MST assay of the interaction between UPCP and PTBP1; *n* = 3 biologically independent replicates. **m**, CoIP of progerin and PTBP1 in HEK-293T cells treatment with or without UPCP. **n**, RIP assay of interaction between PTBP1 and *BUBR1* mRNA 3ʹ-UTR in HEK-293T cells treatment with or without UPCP; *n* = 3 biologically independent replicates. **o**, RNA pulldown assay of *BUBR1* mRNA 3ʹ-UTR or 3ʹ-UTR-mut and PTBP1 in HEK-293T cells treatment with or without UPCP. For bar and line graphs, data are presented as mean ± s.d. Statistical significance was determined in **a**, **b**, **d**, **g**–**i**, **k** and **n** using two-tailed unpaired Student’s *t*-test and in **c** using two-way ANOVA. Source data

To explore the mechanism of UPCP-modulated BUBR1 abundance, we first detected expression of *BUBR1* at the transcriptional level in HGPS cells treated with UPCP. The results showed that UPCP could upregulate expression of *BUBR1* (Fig. 4a). Studies have shown that multiple transcription factors, including Sp1 (ref. ^29^), C-MYC^30^, HMGA1 (ref. ^31^), KDM1A^32^, ZNF143 (ref. ^33^) and FOXM1 (ref. ^34^) regulate *BUBR1* gene transcription. We next examined the effect of UPCP on these transcription factors and found that UPCP had little effect on their expression in HGPS cells (Fig. 4a). Consistently, UPCP also failed to enhance luciferase activity of the *BUBR1* promoter (Fig. 4b), which showed that the abundance of BUBR1 modulated by UPCP may be regulated at the posttranscriptional level. To confirm this, actinomycin D (Act D) was used to block de novo transcription, and we confirmed that the degradation of *BUBR1* mRNA was faster in IMR90-progerin cells compared with control cells (Fig. 4c), implying that progerin accelerates the degradation of *BUBR1* mRNA. Then, we treated HGPS cells with UPCP together with Act D and found that the mRNA levels of *BUBR1* were significantly increased (Fig. 4d), indicating that UPCP upregulated the expression of BUBR1 by stabilizing *BUBR1* mRNA.

**Extended Data Fig. 5:**
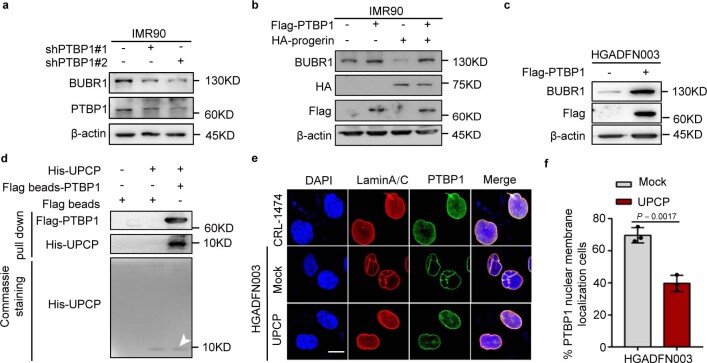
The correlation between PTBP1 and BUBR1. a. Western blot analysis of BUBR1 and PTBP1 in IMR90 cells infected with shRNA (shCtrl, shPTBP1#1 or #2). n = 3 biologically independent replicates. b. Western blot analysis of BUBR1 in PTBP1 overexpression IMR90-progerin cells. n = 3 biologically independent replicates. c. Western blot analysis of BUBR1 in HGADFN003 cells infected with Flag-PTBP1. n = 3 biologically independent replicates. d. Pulldown assay of the interaction between UPCP and PTBP1. Purified His-tagged UPCP was incubated with the Flag beads, Flag beads-PTBP1 (PTBP1 coupled to Flag beads). Total amounts of His-UPCP were visualized by Coomassie brilliant blue staining. The white arrow represents the band of His-UPCP. n = 3 biologically independent replicates. e,f. Immunofluorescence analysis of PTBP1 in CRL-1474 and HGADFN003 cells treated with or without UPCP. Representative images (e) and quantification of the PTBP1 nuclear membrane localization in HGPS cells with the indicated treatments (%) (f). n = 3 biologically independent replicates. Scale bar: 10 μm. For bar and line graphs, data are presented as mean ± SD. Statistical significance was determined in f using two-tailed unpaired Student’s t-test. Source data

To further investigate the possible mechanism of how UPCP stabilizes *BUBR1* mRNA, we used Flag-tagged progerin as bait in mass spectrometry (MS) to survey possible regulatory factors. The results revealed PTBP1 as a progerin-interacting protein (Fig. 4e,f) that is involved in regulating mRNA stability by binding the target gene 3ʹ-UTR^35,36^. To investigate whether PTBP1 affected the stability of BUBR1 mRNA, we first detected the expression of BUBR1 when PTBP1 is deleted in IMR90 cells. We found that the level of BUBR1 was reduced after PTBP1 knockdown in IMR90 cells (Fig. 4g and Extended Data Fig. 5a) and restored by overexpressing PTBP1 in IMR90-progerin cells and HGPS cells (Extended Data Fig. 5b,c). Furthermore, reduction of PTBP1 accelerated the degradation of *BUBR1* mRNA, showing that PTBP1 stabilized *BUBR1* mRNA (Fig. 4h). RNA immunoprecipitation (RIP) and RNA pulldown assays showed that PTBP1 was enriched on the *BUBR1* mRNA 3ʹ-UTR by binding its UCUU motif (Fig. 4i,j). Consistently, PTBP1 enhanced *BUBR1* mRNA 3ʹ-UTR luciferase activity (Fig. 4k). Interestingly, UPCP also combined with PTBP1 and weakened the interaction of PTBP1 and progerin in HGPS cells (Fig. 4l,m and Extended Data Fig. 5d–f), suggesting that UPCP may function via PTBP1. Importantly, UPCP promoted PTBP1 to bind to the 3ʹ-UTR of *BUBR1* mRNA by weakening the interaction of PTBP1-progerin (Fig. 4n,o). Taken together, UPCP enhanced *BUBR1* mRNA stability dependent on PTBP1.

### UPCP alleviates HGPS cellular senescence dependent on BUBR1

**Fig. 5:**
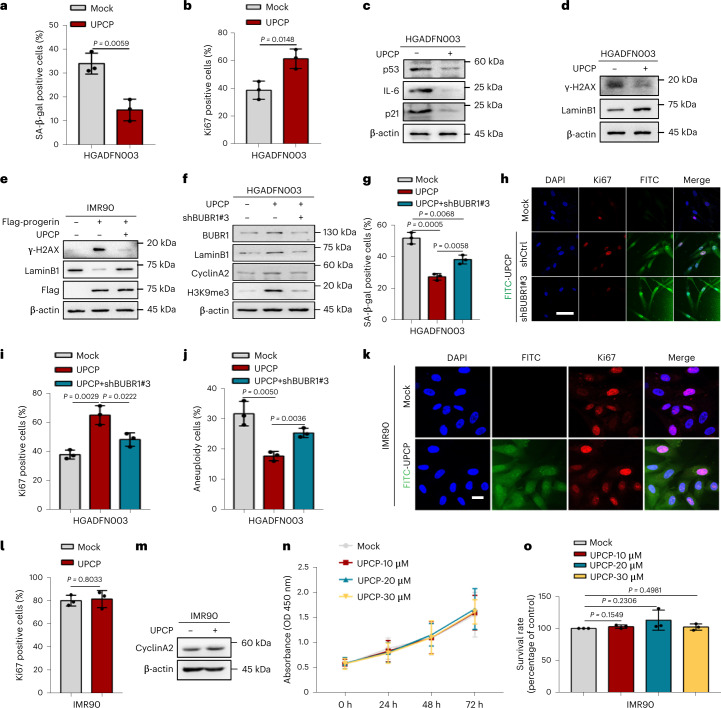
UPCP counteracts cellular senescence depending on BUBR1 in HGPS. **a**, Quantification of SA-β-gal positive cells (%) in HGADFN003 cells treated with or without UPCP; *n* = 3 biologically independent replicates. **b**, Quantification of the Ki67 positive cells (%) in HGADFN003 cells with the indicated treatments; *n* = 3 biologically independent replicates. **c**, Western blot analysis of p53, IL-6 and p21 in HGPS cell (HGADFN003) with the indicated treatment. **d**, Western blot analysis of lamin B1 and γH2AX in HGPS cell (HGADFN003) with or without UPCP (6 μM) treatment for 72 h. **e**, Western blot analysis of lamin B1 and γH2AX in IMR90-progerin cells with or without UPCP (6 μM) treatment for 72 h. **f**, Western blot analysis of BUBR1, lamin B1, CyclinA2 and H3K9me3 in HGADFN003 cells reinhibited the expression of BUBR1 by using BUBR1 shRNA (shBUBR1#3) under treatment with UPCP. **g**, Quantification of SA-β-gal positive cells (%) in HGADFN003 cells with the indicated treatments; *n* = 3 biologically independent replicates. **h**,**i**, Immunofluorescence analysis of Ki67 in HGADFN003 cells with the indicated treatments. Representative images of Ki67 positive cells (**h**) and quantification of the Ki67 positive cells (%) (**i**); *n* = 3 biologically independent replicates. Scale bars, 20 μm. **j**, Quantification of aneuploid cells (%) in HGADFN003 cells with the indicated treatments; *n* = 3 biologically independent replicates. **k**,**l**, Immunofluorescence analysis of Ki67 in IMR90 cells with the indicated treatments. Representative images of Ki67 positive cells (**k**) and quantification of the Ki67 positive cells (%) (**l**); *n* = 3 biologically independent replicates. Scale bars, 20 μm. **m**, Western blot analysis of CyclinA2 in IMR90 cells treatment with or without UPCP. **n**, CCK8 assay of cell viability in IMR90 cells with the indicated treatments; *n* = 3 biologically independent replicates. **o**, Cell survival rate analysis in IMR90 cells with the indicated treatments; *n* = 3 biologically independent replicates. For bar and line graphs, data are presented as mean ± s.d. Statistical significance was determined in **a**, **b**, **g**, **i**, **j**, **l** and **o** using two-tailed unpaired Student’s *t*-test and in **n** using two-way ANOVA. Source data

**Extended Data Fig. 6:**
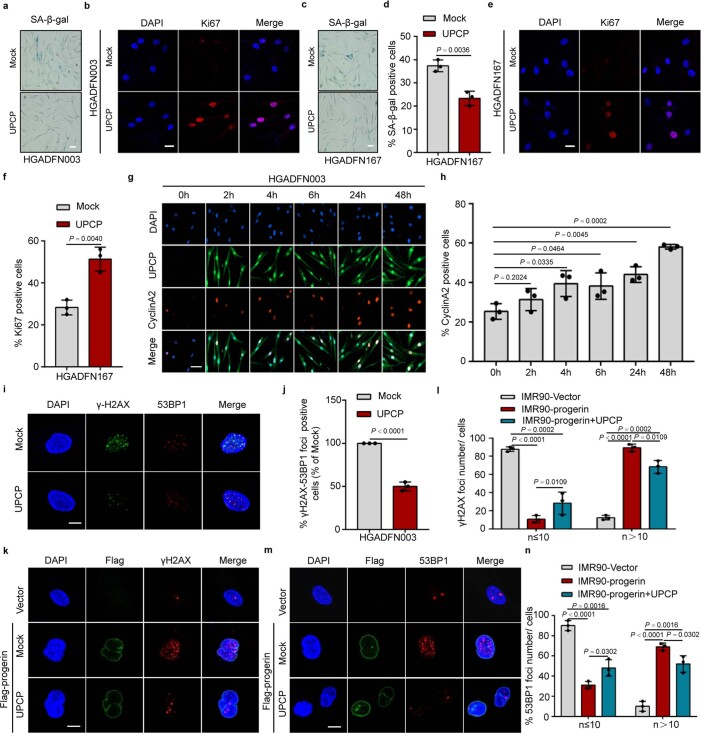
UPCP alleviates HGPS cellular senescence. a. Representative images of SA-β-Gal positive cells in HGADFN003 cells. Scale bars: 100 μm. b. Representative images of Ki67 positive cells in HGADFN003 cells. Scale bars: 20 μm. c,d. β-Gal staining analysis of HGADFN167 cells with the indicated treatments. Representative images (c) and quantification of the SA-β-Gal positive cells (%) (d). n = 3 biologically independent replicates. Scale bars: 100 μm. e,f. Immunofluorescence analysis of Ki67 in HGADFN167 cells with the indicated treatments. Representative images (e) and quantification of the Ki67 positive cells (%) (f). n = 3 biologically independent replicates. Scale bars: 20 μm. g,h. Immunofluorescence analysis of CyclinA2 in HGADFN003 cells with the indicated treatments. Representative images of CyclinA2 positive cells (g) and quantification of the CyclinA2 positive cells (%) (h). n = 3 biologically independent replicates. i,j. Immunofluorescence analysis of γH2AX and 53BP1 in HGPS cells (HGADFN003) with the indicated treatment. Representative images of γH2AX-53BP1 foci (i) and quantification of the γH2AX-53BP1 foci positive cells (%) (j). n = 3 biologically independent replicates. Scale bars: 10 μm. k,l. Immunofluorescence analysis of γH2AX foci and Flag-progerin in IMR90-progerin cells with the indicated treatment. Representative images of γH2AX-progerin double staining (k) and quantification of γH2AX foci number per nucleus (%) (l). n = 3 biologically independent replicates. Scale bars: 10 μm. m,n. Immunofluorescence analysis of 53BP1 and Flag-progerin in IMR90-progerin cells with the indicated treatment. Representative images of 53BP1-progerin double staining (m) and quantification of 53BP1 foci number per nucleus (%) (n). n = 3 biologically independent replicates. Scale bars: 10 μm. For bar and line graphs, data are presented as mean ± SD. Statistical significance was determined in d, f, h, j, l and n using two-tailed unpaired Student’s t-test. Source data

**Extended Data Fig. 7:**
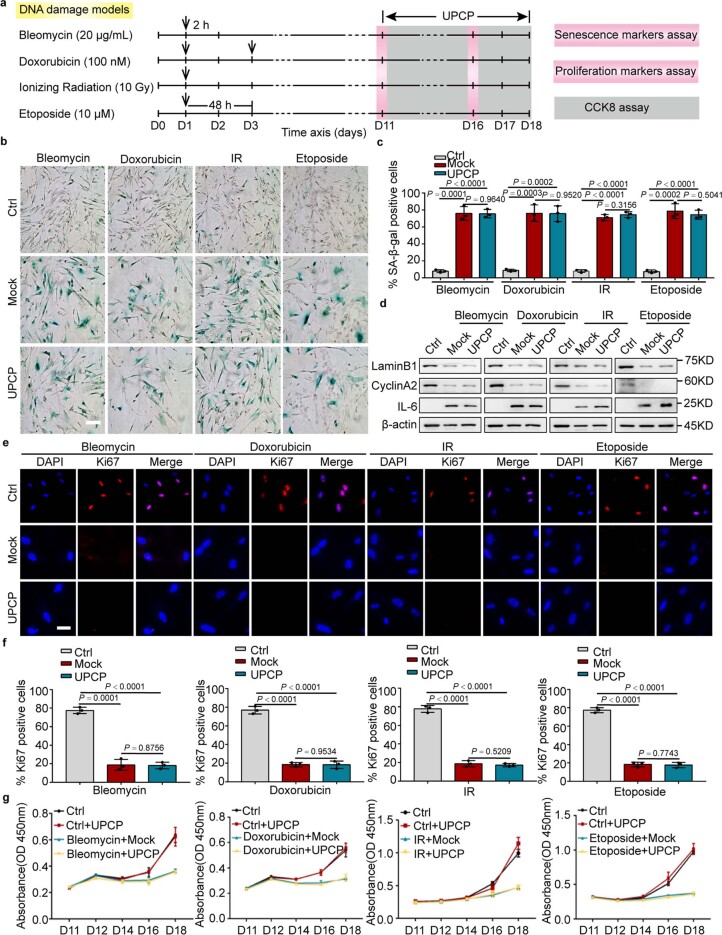
UPCP has little effect on the proliferation of deep senescent cells-induced by different DNA damage stimuli. a. Schematic diagram of the time-axis for different DNA damage strategies[Bleomycin (20 μg/mL for 2 h), Doxorubicin (100 nM treatment twice for a 2 days interval), Ionizing Radiation (IR) (10 Gy Gamma rays) and Etoposide (10 μM for 48 h)]. b,c. β-Gal staining analysis of IMR90 cells (after DNA-damaging attack for 10 days-induced by different DNA damage models [Bleomycin, Doxorubicin, Ionizing Radiation (IR) and Etoposide] treatment with or without UPCP (6 μM) for another 5 days. Representative images (b) and quantification of SA-β-gal positive cells (%) (c). n = 3 biologically independent replicates. Scale bars: 200 μm. d. Western blot analysis of Lamin B1, CyclinA2 and IL-6 in senescent IMR90 cells with the indicated treatments. n = 3 biologically independent replicates. e,f. Immunofluorescence analysis of Ki67 in IMR90 cells with the indicated treatment. Representative images (e) and quantification of the Ki67 positive cells (%) (f). n = 3 biologically independent replicates. Scale bars: 50 μM. g. CCK8 assay of cell viability in IMR90 cells with the indicated treatments. n = 3 biologically independent replicates. For bar and line graphs, data are presented as mean ± SD. Statistical significance was determined in c and f using two-tailed unpaired Student’s t-test and in g using two-way ANOVA. Source data

We have confirmed both that UPCP upregulated BUBR1 expression (Fig. 3) and the effect of BUBR1 on antagonizing HGPS cellular senescence (Fig. 1), suggesting that UPCP represents a new approach to rejuvenating aged HGPS cells. To address this, we examined the effects of UPCP on HGPS cells. HGPS cellular senescence was clearly ameliorated by UPCP (Fig. 5a–e and Extended Data Fig. 6), including the reduced activity of SA-β-gal, the increased Ki67 positive staining, the decreased level of p53, IL-6 and p21 and the increased level of CyclinA2 (Fig. 5a–c and Extended Data Fig. 6a–h). Moreover, UPCP could decrease DNA damage, accompanied with a lower level of γH2AX-53BP1 foci in HGPS fibroblasts and IMR90-progerin cells (Fig. 5d,e and Extended Data Fig. 6i–n). To further evaluate the direct role of UPCP in regulating BUBR1 in HGPS cellular senescence, HGPS cells were transduced with lentiviruses to inhibit BUBR1 after treatment with UPCP. We found that depletion of BUBR1 significantly antagonized the senescence remission mediated by UPCP in HGPS cells (Fig. 5f–j). Thus, UPCP alleviated HGPS cellular senescence through BUBR1. Moreover, we found that UPCP had little effect on proliferation of IMR90 cells (Fig. 5k–o). Also, we investigated the roles of UPCP on the DNA damage stimuli-induced cellular senescence (DNA-damaging attack for 10 days—induced by bleomycin, doxorubicin, ionizing radiation or etoposide) (Extended Data Fig. 7a and Supplementary Data Fig. 1). We found that UPCP did not cause DNA damage-induced senescent cells to proliferate, and failed to rescue the progeria phenotype of these cells (Extended Data Fig. 7b–g), suggesting that UPCP might play a specific role in alleviating HGPS senescence.

### UPCP ameliorates progeroid features of *Lmna*^*G609G/G609G*^ mice

**Fig. 6:**
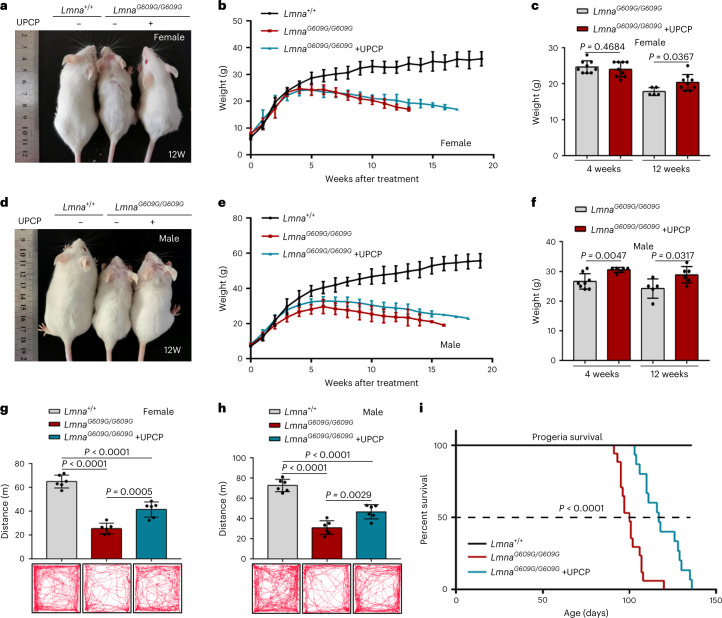
UPCP extends lifespan in *Lmna*^*G609G/G609G*^ mice. **a**, Representative photographs of treatment of *Lmna*^*G609G/G609G*^ female mice with UPCP for 12 weeks (12W). **b**, Progression of bodyweight of female mice treated with PBS or UPCP (*n* = 6 *Lmna*^*+/+*^ mice; *n* = 9 PBS-treated *Lmna*^*G609G/G609G*^ mice; *n* = 9 UPCP-treated *Lmna*^*G609G/G609G*^ mice). **c**, Bodyweight comparison between 4 and 12 weeks after treatment with UPCP in female mice (*n* = 5 or 9 PBS-treated *Lmna*^*G609G/G609G*^ mice; *n* = 9 UPCP-treated *Lmna*^*G609G/G609G*^ mice). **d**, Representative photographs of treatment of *Lmna*^*G609G/G609G*^ male mice with UPCP for 12 weeks. **e**, Progression of bodyweight of male mice treated with PBS or UPCP (*n* = 11 *Lmna*^*+/+*^ mice; *n* = 8 PBS-treated *Lmna*^*G609G/G609G*^ mice; *n* = 6 UPCP-treated *Lmna*^*G609G/G609G*^ mice). **f**, Bodyweight comparison between 4 and 12 weeks after treatment with UPCP in male mice (*n* = 5 or 9 PBS-treated *Lmna*^*G609G/G609G*^ mice; *n* = 6 UPCP-treated *Lmna*^*G609G/G609G*^ mice). **g**,**h**, Open field test showed motion capabilities in female (**g**) and male (**h**) mice (*n* = 6 for each group). **i**, Kaplan–Meier survival plot of PBS versus UPCP-treated *Lmna*^*G609G/G609G*^ mice (*n* = 18 *Lmna*^*+/+*^ mice; *n* = 17 PBS-treated *Lmna*^*G609G/G609G*^ mice; *n* = 15 UPCP-treated *Lmna*^*G609G/G609G*^ mice. For bar and line graphs, data are presented as mean ± s.d. Statistical significance was determined in **c** and **f**–**h** using two-tailed unpaired Student’s *t*-test. Source data

**Extended Data Fig. 8:**
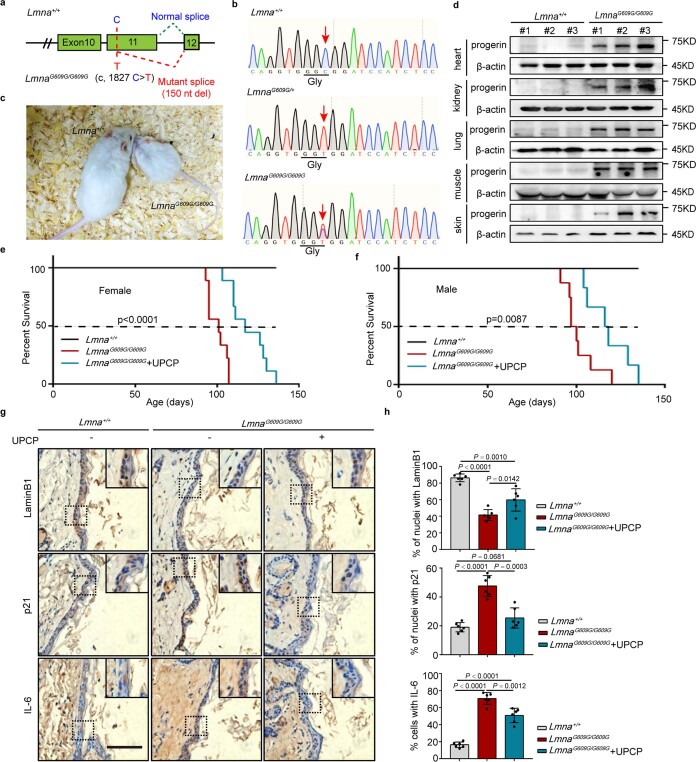
UPCP ameliorates cellular senescence in skin of *Lmna*^*G609G/G609G*^ mice and extends lifespan of *Lmna*^*G609G/G609G*^ mice. a. Schematic diagram of target sites located in exon 11 of the *Lmna*^*G609G/G609G*^. The target bases are marked in blue or red. Green and red lines indicate the normal and mutant mRNA splice forms, respectively. Mutant mRNA splicing results in a hypothetical 150-nucleotide deletion. b. Sanger sequencing chromatograms from *Lmna*^*+/+*^, *Lmna*^*G609G/+*^ and *Lmna*^*G609G/G609G*^ mouse. The red arrows indicate the substituted nucleotide. Relevant codon identities at the target site are represented below the DNA sequence. c. Representative photographs of 3 months *Lmna*^*+/+*^ and *Lmna*^*G609G/G609G*^ mice. d. Western blot analysis of progerin in heart, kidney, lung, muscle and skin from *Lmna*^*+/+*^ and *Lmna*^*G609G/G609G*^ mice. n= 3 for each group. e. Kaplan–Meier survival plot of PBS versus UPCP-treated *Lmna*^*G609G/G609G*^ mice (female, n=9 *Lmna*^*+/+*^ mice; n=9 PBS-treated *Lmna*^*G609G/G609G*^ mice; n=9 UPCP-treated *Lmna*^*G609G/G609G*^ mice). f. Kaplan–Meier survival plot of PBS versus UPCP-treated *Lmna*^*G609G/G609G*^ mice (male, n=9 *Lmna*^*+/+*^ mice; n=8 PBS-treated *Lmna*^*G609G/G609G*^ mice; n=6 UPCP-treated *Lmna*^*G609G/G609G*^ mice). g,h. Immunohistochemistry of Lamin B1, p21 and IL-6 in skin from *Lmna*^*+/+*^ and *Lmna*^*G609G/G609G*^ mice treated with PBS or UPCP. Representative images of LaminB1, p21 and IL-6 (c) and quantification of LaminB1, p21 and IL-6 was calculated (d). n=6. Scale bars: 100 μm. For bar and line graphs, data are presented as mean ± SD. Statistical significance was determined in h using two-tailed unpaired Student’s t-test. Source data

To evaluate the therapeutic potential of UPCP in vivo, we successfully generated a mouse model (ICR background) with the causative HGPS *Lmna*^*G609G*^ mutation using the BE4-Gam system^37,38^ (Extended Data Fig. 8a–d). Subsequently, 20 mg kg^–1^ of UPCP was injected intraperitoneally every other day into 14-day-old *Lmna*^*G609G/G609G*^ mice. We found that injection of UPCP ameliorated the bodyweight of male and female *Lmna*^*G609G/G609G*^ mice in different ways (Fig. 6a–f). For female *Lmna*^*G609G/G609G*^ mice, UPCP could slow down the bodyweight loss (Fig. 6b,c). In contrast, UPCP could increase the bodyweight of male *Lmna*^*G609G/G609G*^ mice (Fig. 6e,f). Moreover, injection of UPCP enhanced the mobility of *Lmna*^*G609G/G609G*^ mice (Fig. 6g,h). Importantly, the median survival of *Lmna*^*G609G/G609G*^ mice treated with UPCP was extended compared with that of PBS-treated mice, from 100 to 117 days, which represents a 17% increase in lifespan. The mean survival was extended from 100.9 days to 118.6 days (Fig. 6i and Extended Data Fig. 8e,f).

**Fig. 7:**
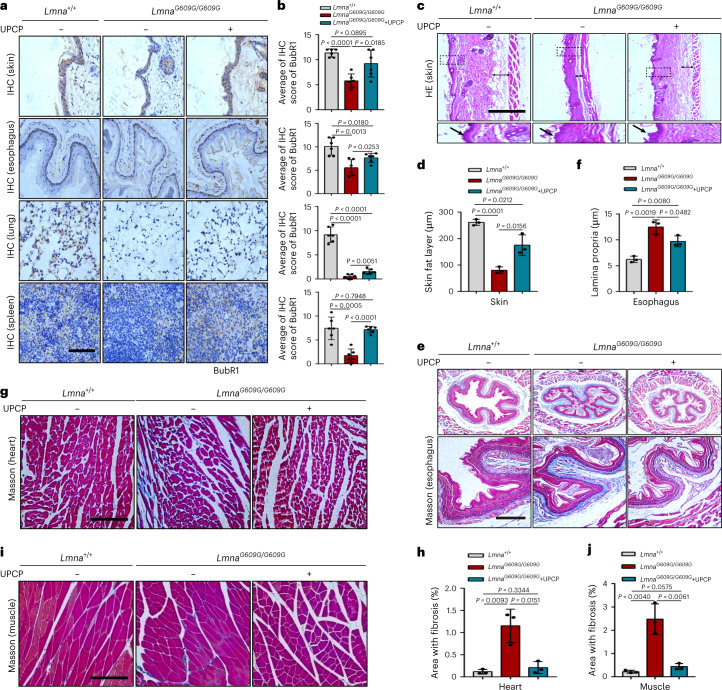
UPCP ameliorates progeroid phenotypes in *Lmna*^*G609G/G609G*^ mice. **a**,**b**, IHC of BubR1 in skin, esophagus, lung and spleen from *Lmna*^*+/+*^ and *Lmna*^*G609G/G609G*^ mice treated with PBS or UPCP. Representative images of BubR1 in skin, esophagus, lung and spleen are shown (**a**) and quantification of BubR1 scored (**b**). Scale bars, 100 μm; *n* = 6. **c**,**d**, Histological analyses of the skin derived from mice treatment with UPCP for 13 weeks versus PBS-treated *Lmna*^*G609G/G609G*^ mice next to mock-treated *Lmna*^*+/+*^ mice. The dashed square box and the single arrow represent the skin of the zoom area and double arrow area represents subcutaneous fat layers. Scale bars, 250 μm (**c**). Measurement of the subcutaneous fat layers was done in ImageJ (*n* = 3 sections per group from three biologically independent samples, three measurements were selected randomly in each section) (**d**). **e**,**f**, Masson staining of esophagus derived from mice treatment with UPCP for 13 weeks versus PBS-treated *Lmna*^*G609G/G609G*^ mice next to mock-treated *Lmna*^*+/+*^ mice. The dashed square box represents a local amplification of esophagus and fibrosis in lamina propria (blue areas) (**e**). Scale bars, 100 μm. The lamina propria was measured in ImageJ (*n* = 3 sections per group from three biologically independent samples, three measurements were selected randomly in each section) (**f**). **g**,**h**, Masson staining of heart derived from mice treatment with UPCP for 13 weeks versus PBS-treated *Lmna*^*G609G/G609G*^ mice next to mock-treated *Lmna*^*+/+*^ mice. Representative photographs of fibrosis in heart (**g**). Scale bars, 100 μm. Fibrosis in heart (**h**) (blue areas) was measured in ImageJ (*n* = 3 for each group). **i**,**j**, Masson staining of muscle derived from mice treatment with UPCP for 13 weeks versus PBS-treated *Lmna*^*G609G/G609G*^ mice next to mock-treated *Lmna*^*+/+*^ mice. Representative photographs of fibrosis in muscle (**i**). Scale bars, 150 μm. Fibrosis in muscle (**j**) (blue areas) was measured in ImageJ (*n* = 3 for each group). For bar and line graphs, data are presented as mean ± s.d. Statistical significance was determined in **b**, **d**, **f**, **h** and **j** using two-tailed unpaired Student’s *t*-test. Source data

To obtain more detailed evidence, we detected the levels of lamin B1, p21 and SASP factor (IL-6) in the skin tissue of *Lmna*^*G609G/G609G*^ mice. Histological analysis showed that UPCP could increase the number of lamin B1 positive cells and reduce p21 and IL-6 expression in the skin of the *Lmna*^*G609G/G609G*^ mice (Extended Data Fig. 8g,h). Also, we measured the expression of BubR1 in the various tissues of *Lmna*^*G609G/G609G*^ mice after treatment with UPCP in comparison with negative controls. We found that UPCP increased the expression of BubR1 in the skin, esophagus, lung and spleen from *Lmna*^*G609G/G609G*^ mice, which is similar to observations in human cells (Fig. 7a,b). Moreover, histological analysis revealed that UPCP improved two aging-associated skin conditions: epidermal thinning (Fig. 7c) and dermal fat loss (Fig. 7d). In addition, UPCP reduced fibrosis of lamina propria in the esophagus and heart of *Lmna*^*G609G/G609G*^ mice and decreased the thickness of the lamina propria in the esophagus (Fig. 7e–h). Consistently, a decrease of fibrosis in the muscle of *Lmna*^*G609G/G609G*^ mice was observed after treatment with UPCP (Fig. 7i,j), which is one of the main systems impaired during aging and in HGPS. Collectively, UPCP treatment ameliorates progeroid features and extends lifespan in *Lmna*^*G609G/G609G*^ mice.

## Discussion

HGPS is caused by the accumulation of progerin^3^. Thus far, the role and mechanism of cell defects induced by progerin remain unclear in HGPS cellular senescence. In this study, we unveiled a new regulatory mechanism of progerin-PTBP1-BUBR1 in HGPS cellular senescence. We showed that accumulation of progerin resulted in decreased expression and mislocalization of BUBR1, which affected its role in the SAC signal response, accelerating HGPS cellular senescence. Indeed, BUBR1 rescued HGPS cellular senescence and showed strong binding affinity for progerin-C. On this basis, we developed a small peptide (UPCP) that blocks the interaction between progerin and BUBR1 and concurrently modulates the abundance of BUBR1 dependent on PTBP1. UPCP reversed HGPS cellular senescence and showed an effect on the progerin-dependent progeria model

Recently, studies have suggested that sustaining high BUBR1 levels helps to preserve genomic integrity by attenuation of SAC defects, improper kinetochore–microtubule attachment and age-associated decline^18,27^. Here, we first confirmed that BUBR1 was involved in the process of HGPS cellular senescence. Our data showed that progerin decreased the abundance of BUBR1 and anchored BUBR1 to the nuclear membrane (Figs. 1 and 2). In previous studies, the abundance of BUBR1 was assessed mainly at the transcriptional^29–34^ and the posttranslational^39–42^ level; here, we focused on the posttranscriptional level of BUBR1. Our results demonstrated that PTBP1 stabilized *BUBR1* mRNA by binding to the *BUBR1* 3ʹ-UTR (Fig. 4g–j). However, PTBP1 could not bind to the *BUBR1* 3ʹ-UTR after interacting with progerin, which decreased the stability of the *BUBR1* mRNA (Fig. 4n,o). In addition, we found that BUBR1 interacted with the lamin A, and BUBR1 did not localize to the nuclear membrane in normal interphase cells (Fig. 2a,d,e). Thus, the interaction between BUBR1 and lamin A seem to be reversible. However, BUBR1 could be anchored to the nuclear membrane by the stronger interaction with progerin in interphase cells, which inhibited the role of BUBR1 in the SAC signal response during mitosis (Fig. 2a,g–m). In these cases, a combination of decreased abundance and mislocalization of BUBR1 cause the loss of BUBR1 function, which promotes the occurrence and development of HGPS. However, the sequence of BUBR1 reduction and mislocalization mediated by progerin remain to be further explored during HGPS cellular senescence.

**Extended Data Fig. 9:**
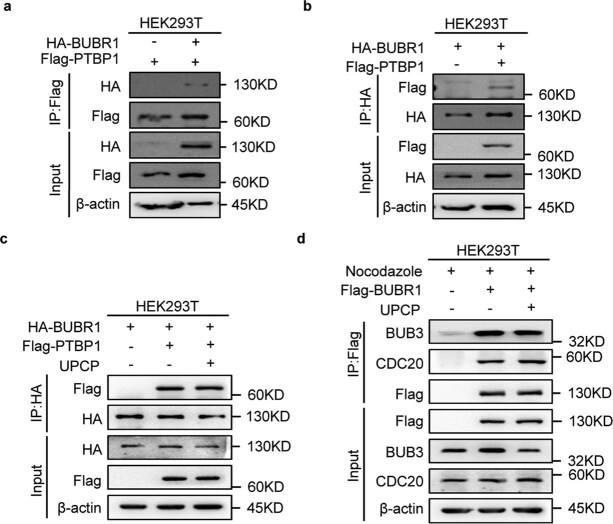
UPCP does not block the binding of BUBR1 to other proteins. a,b. Co-IP assay of PTBP1 and BUBR1 in HEK-293T cells. HEK-293T cells were co-transfected with Flag-PTBP1 and HA-BUBR1. PTBP1 or BUBR1 was IP from HEK-293T cells lysate with the anti-Flag or anti-HA antibody. n = 3 biologically independent replicates. c. Co-IP assay of PTBP1 and BUBR1 in HEK-293T cells treated with or without UPCP (18 μM). HEK-293T cells were co-transfected with Flag-PTBP1 and HA-BUBR1, then treated with or without UPCP for 48 h. BUBR1 was IP from HEK-293T cells lysate with the anti-HA antibody. n = 3 biologically independent replicates. d. Co-IP assay of BUBR1 and SAC proteins (BUB3 and CDC20) in HEK-293T cells treated with or without UPCP (18 μM) under the condition of being exposed to Nocodazole (200 ng ml^–1^,a microtubule-disrupting agent, which could activate the SAC signal response). n = 3 biologically independent replicates.

Studies have shown that progerin anchors to the nuclear membrane due to the retention of farnesylation modifications^9^ and impairment of multicellular stress responses pathways by sequestering different proteins^43,44^. Therefore, it is necessary to find an effective inhibitor that blocks the interaction of progerin with other proteins. It has been shown that JH4 can block pathological progerin-lamin A/C binding, ameliorating the HGPS phenotype^28^. Here, we developed a new peptide, UPCP, which interfered with the interaction of progerin and BUBR1 and modulated BUBR1 abundance (Fig. 3f–l). To explore the possible mechanism of UPCP-modulated BUBR1 abundance, based on the MS data, we found that UPCP released another progerin-interacting protein PTBP1 (Fig. 4f,m). The interaction between UPCP and PTBP1 also released PTBP1 from progerin and promoted PTBP1 to stabilize BUBR1 mRNA, further upregulating the level of BUBR1 (Fig. 4). In total, the dual role of UPCP can restore the function of BUBR1 to a certain extent, and thus alleviate HGPS cellular senescence. Besides, we showed that BUBR1 interacted with PTBP1 (Extended Data Fig. 9a,b) and UPCP did not block the binding of BUBR1 to other proteins such as PTBP1 or other SAC proteins (BUB3 or CDC20) (Extended Data Fig. 9c,d). The data showed that the interaction between UPCP and BUBR1 released BUBR1 from progerin to play roles in mitosis, which means that UPCP does not limit the function of BUBR1. It remains to be further explored how UPCP blocks the interaction between BUBR1 or PTBP1 and progerin, simultaneously or continuously. Strong evidence for protein sequestration at the nuclear periphery has been demonstrated as a potential regulatory mechanism in premature aging^43,45^. Whether UPCP can interfere with the interaction between progerin and its binding partners remains to be explored in the future.

**Extended Data Fig. 10:**
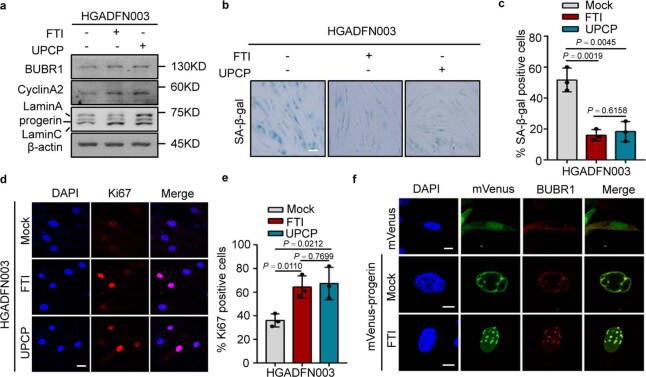
FTI and UPCP rescue HGPS cellular senescence. a. Western blot analysis of BUBR1 and CyclinA2 in HGADFN003 cells treatment with FTI or UPCP. n = 3 biologically independent replicates. b,c. β-Gal staining analysis of HGADFN003 cells with the indicated treatments. Representative images (b) and quantification of SA-β-gal positive cells (%) (c). n = 3 biologically independent replicates. d,e. Immunofluorescence analysis of Ki67 in HGADFN003 cells with the indicated treatments. Representative images (d) and quantification of the Ki67 positive cells (e). n = 3 biologically independent replicates. Scale bars: 20 μm. f. Immunofluorescence analysis of BUBR1 in IMR90 cells with the indicated treatments. n = 3 biologically independent replicates. Scale bars: 10 μm. For bar and line graphs, data are presented as mean ± SD. Statistical significance was determined in c and e using two-tailed unpaired Student’s t-test. Source data

HGPS is an extremely rare congenital genetic disease, and its treatments are still under theoretical research^46^. Although the application of gene editing technologies can significantly improve the aging phenotype of HGPS mice and extend their lifespan^47–52^, it remains unknown whether the application of gene editing can increase the risk of cancer. Currently, although the clinical application of farnesyltransferase inhibitors can extend patients’ lives, it does not completely prevent the accumulation of progerin, accompanied by adverse reactions such as diarrhea and vomiting, and these factors limit the effect of farnesyltransferase inhibitors as a means of treatment^22^. Our data demonstrate that UPCP relieves the efficacy of HGPS cellular senescence, comparable with farnesyltransferase inhibitors (FTI-277). Both UPCP and FTI-277 decreased the activity of SA-β-gal, accompanied with increasing expression of BUBR1, CyclinA2 and Ki67 (Extended Data Fig. 10a–e). However, we found that, although FTI-277 could upregulate expression of BUBR1, it failed to block the interaction of BUBR1 and progerin, which still limited the function of BUBR1 to a certain extent (Extended Data Fig. 10f). UPCP, as a peptide, had no obvious effect on the proliferation of normal cells (Fig. 5k–o). Also, to investigate the role of UPCP on other senescence models, we used Etoposide, Ras or H_2_O_2_ to induce senescence of IMR90 cells, and added UPCP to the cells treated with the drugs mentioned above when the cells just entered early stage senescence. We found that UPCP delayed etoposide-, ras- and H_2_O_2_-induced early stage cellular senescence modestly (Supplementary Data Fig. 2). Although UPCP delayed the phenotype of such senescent cells to a small extent, the senescent cells remaining did not recover like the control group and the proliferation rate is still far lower than that of the control group. Furthermore, UPCP did not cause DNA damage stimuli-induced deep senescent cells to proliferate and failed to rescue the progeria phenotype of these cells (Extended Data Fig. 7), which means that UPCP could just delay early phase cellular senescence to a certain extent and had little effect on the proliferation and phenotype of deep senescent cells-induced by different DNA damage stimuli, suggesting that UPCP does not induce a senescence escape of DNA damage-induced senescence. Particularly, UPCP ameliorated HGPS cellular senescence and progeroid features of *Lmna*^*G609G/G609G*^ mice (Figs. 5–7), suggesting that UPCP might be specific in alleviating HGPS senescence, which is a prerequisite for subsequent translation of HGPS therapeutic strategy. Further work aimed at obtaining UPCPs with stronger stability, longer half-life and optimized physical structures are under planning. Therefore, UPCP may have broad application prospects in the treatment of HGPS.

## Methods

### Cell culture

Cell lines (IMR90, CRL-1474, HEK-293T, NIH-3T3 and H1299) were obtained from the American Type Culture Collection (ATCC). Fibroblasts from patients with HGPS were obtained from The Progeria Research Foundation Cell and Tissue Bank (http://www.progeriaresearch.org). The following fibroblasts were used: HGADFN003 (2 years old), HGADFN167 (8 years old), HGMDFN090 (37 years old) and HGFDFN168 (40 years old). Human dermal fibroblasts (GM00038, 9 years old and AG09602, 92 years old) were obtained from Coriell Cell Repository (https://catalog.coriell.org). These cells were characterized by DNA fingerprinting and isozyme detection and were tested by a MycoBlue Mycoplasma Detector (Vazyme Biotech) to exclude mycoplasma contamination before the experiments. IMR90, CRL-1474, HEK-293T, NIH-3T3 and H1299 cells were cultured in DMEM medium (Sigma-Aldrich) with 10% (v/v) fetal bovine serum (VivaCell). HGADFN003, HGADFN167, HGMDFN090, HGFDFN168, GM00038 and AG09602 were cultured in DMEM medium with 15% (v/v) fetal bovine serum (VivaCell). All cell lines were grown at 37 °C with 5% CO_2_ and IMR90, CRL-1474, HGADFN003, HGADFN167, HGMDFN090, HGFDFN168, GM00038 and AG09602 were cultured with 5% O_2_. For bleomycin-induced senescence, IMR90 cells were incubated with the culture medium containing 20 μg ml^–1^ Bleomycin (Selleck, catalog no. S1214) for 2 h and analyzed 10 days later, or as otherwise indicated. For doxorubicin-induced senescence, IMR90 cells were treated twice with 100 nM Doxorubicin (Selleck, catalog no. S1208) with a 2 day interval and analyzed 10 days later, or as otherwise indicated. For ionizing radiation (IR)-induced senescence, IMR90 cells were exposed to 10 Gy Gamma rays, and analyzed 10 days later, or as otherwise indicated, the control IMR90 cells were mock irradiated^25^. For etoposide-induced senescence, IMR90 cells incubated with the culture medium containing 10 μM Etoposide (Sigma, catalog no. E1383) for 48 h and analyzed 10 days later, or as otherwise indicated^53^.

### Plasmid construction

Restriction enzymes and T4 DNA ligase were obtained from New England BioLabs (NEB). *Bam*HI-HF (catalog no. R3136), *Not*I-HF (catalog no. R3189), *Eco*RI-HF (catalog no. R3101), *Bgl*II (catalog no. R0144), *Kpn*I-HF (catalog no. R3142), *Age*I (catalog no. R0552) and T4 DNA Ligase (catalog no. M0202) were used in this study.

DNA fragments of 3×Flag/HA-tagged progerin/lamin A and a series of truncated progerin or lamin A proteins were inserted into the lentiviral pCDH-CMV-puro expression vector. These target DNA fragments were cloned from the plasmids: pBabe-puro-GFP-wt-lamin A (catalog no. 17662) and pBabe-puro-GFP-progerin (catalog no. 17663), which were purchased from Addgene. GST-tagged progerin/lamin A and a series of truncated progerin or lamin A proteins were made by inserting the above target DNA fragments above into the pGEX-6P-1 vector in frame with the GST coding sequence.

Flag/HA-tagged BUBR1 plasmid and truncated BUBR1 were cloned from the pEGFP-C1-BUBR1 plasmid, which was a gift from X. Yao (University of Science and Technology of China). Target DNA fragments were constructed into the expression vector pCDH-CMV-3×Flag-puro and pcDNA3.1.

The DNA fragment of *BUBR1* mRNA 3′-UTR was inserted into the pGL4.20 vector. The pcDNA3-Flag-MS2bp and pcDNA3-12×MS2bs plasmids were kindly provided by X. Zheng (Academy of Military Medical Sciences, China). The 3′-UTR or 3′-UTR-△UCUU of *BUBR1* mRNA was inserted into the pcDNA3-12×MS2bs plasmid. pWPXLd-Flag-PTBP1, pDSL-hpUGIP, pDSL-hpUGIP-PTBP1#1, and pDSL-hpUGIP-PTBP1#2 were provided by P. Hou (Cancer Institute, Xuzhou Medical University, China).

The DNA fragment of UPCP was inserted into the vector pET28a(+).

Human BUBR1 shRNAs were cloned into pLKO.1 plasmid (pLKO.1-BUBR1#1 and pLKO.1-BUBR1#3). The sequences of shRNAs are described below: shCtrl_fwd (5′-CCGGAATGCCTACGTTAAGCTATACCTCGAGGTATAGCTTAACGTAGGCATTTTTTTG-3′) and shCtrl_rev (5′-AATTCAAAAAAATGCCTACGTTAAGCTATACCTCGAGGTATAGCTTAACGTAGGCATT-3′); shBUBR1#1_fwd (5′-CCGGGCAGAGAAGAGAGCAGAAACTCGAGTTTCTGCTCTCTTCTCTGCTTTTTG-3′) and shBUBR1#1_rev (5′-AATTCAAAAAGCAGAGAAGAGAGCAGAAACTCGAGTTTCTGCTCTCTTCTCTGC-3′); shBUBR1#3_fwd (5′-CCGGTGCAAGAAGAGACGGAGAACTCGAGTTCTCCGTCTCTTCTTGCATTTTTG-3′) and shBUBR1#3_ rev (5′-AATTCAAAAATGCAAGAAGAGACGGAGAACTCGAGTGCAAGAAGAGACGGAGAACTC-3′).

### Lentiviral production and infection

Detailed descriptions were performed as previously described^53^. The packaging/envelope plasmids pMDLg/RRE, VSVG and pRSV-Rev were transfected into HEK-293T together with pCDH-CMV-puro or pLKO.1/pDSL-hpUGIP. Polyethylenimine (PEI), as a transfection reagent, was purchased from Sigma. The generation of virus in HEK-293T cells and transfection of viral constructs into recipient cell lines were performed according to the manufacturer’s instructions (Invitrogen).

### UPCP peptide development

UPCP consists of the following amino acid sequence in l-isoform:

H-YGRKKRRQRRRYNLRSRTVLCGTCGQPADKASASGSGAQSPQNCSIM-OH; MW, 5,704.92. It was manufactured by GL Biochem Ltd and ChinaPeptides Co. at greater than 95% purity and stored at –20°C in 1 mg powder aliquots until used to avoid freeze–thawing artifacts. For in vitro experiments, UPCP was dissolved in PBS to generate a 2 mM stock. For in vivo use, UPCP was dissolved in PBS to generate a 5 mg ml^–1^ stock solution, which was kept on ice until injection. Before injection, the solution was brought to room temperature.

### RNA preparation and reverse transcription-PCR

RNA preparation was performed as described^54^. Total RNA was extracted using Trizol reagent (TaKaRa) following the manufacturer’s instructions. cDNA was generated with M-MLV reverse transcriptase (Promega, catalog no. M170B). Reverse transcription with PCR (RT-PCR) was performed with SYBR Green Real-Time PCR Master Mix (TaKaRa). RT-PCR was carried out on a QuantStudio 3 Real-Time-PCR Instrument (ABI). Data were analyzed and further processed in QuantStuido Design and Analysis Software and GraphPad Prism v.7. Fold change in gene expression over control samples was calculated using the 2^–ΔΔCt^ method, where β-actin Ct values were used as an internal control. The primer sequences for PCR, synthesized by Comate Bioscience, were as follows:

β-actin_fwd (5′-GAGCACAGAGCCTCGCCTTT-3′) and β-actin_rev (5′-ATCCTTCTGACCCATGCCCA-3′); AURKB_fwd (5′-CAGTGGGACACCCGACATC-3′) and AURKB_rev (5′-GTACACGTTTCCAAACTTGCC-3′); BUB1_ fwd (5′-ACACCATTCCACAAGCTTCC-3′) and BUB1_rev (5′-CGCCTGGGTACACTGTTTTG-3′); BUB1B_fwd (5′-TGGAAGAGACTGCACGACAG-3′) and BUB1B_ rev (5′-CAGGCTTTCTGGTGCTTAGG-3′); CCNA2_fwd (5′-TTCATTTAGCACTCTACACAGTCACGG-3′) and CCNA2_ rev (5′-TTGAGGTAGGTCTGGTGAAGGTCC-3′); Mad2l1_fwd (5′-GACATTTCTGCCACTGTTGG-3′) and Mad2l1_ rev (5′-AACTGTGGTCCCGACTCTTC-3′); TTK1_fwd (5′-ACCAAGCAGCAATACCTTGG-3′) and TTK1_ rev (5′-ACTGACAAGCAGGTGGAAAG-3′); PLK1_fwd (5′-CAGTCACTCTCCGCGACAC-3′) and PLK1_ rev (5′-GAGTAGCCGAATTGCTGCTG-3′); PTBP1_fwd (5′-AGCGCGTGAAGATCCTGTTC-3′) and PTBP1_ rev (5′-CAGGGGTGAGTTGCCGTAG-3′); HMGA1_fwd (5′-AGCGAAGTGCCAACACCTAAG-3′) and HMGA1_ rev (5′-TGGTGGTTTTCCGGGTCTTG-3′); KDM1A1_fwd (5′-AGCGTCATGGTCTTATCAA-3′) and KDM1A1_ rev (5′-GAAATGTGGCAACTCGTC-3′); ZNF143_fwd (5′-GTACAGGGGACAGTTTGCGTC-3′) and ZNF143_ rev (5′-TGGAGGTGTGGTGAATAAATGC-3′); FOXM1_fwd (5′-CGTCGGCCACTGATTCTCAAA-3′) and FOXM1_ rev (5′-GGCAGGGGATCTCTTAGGTTC-3′).

### RNA-seq analysis

Total RNA samples were isolated and prepared at APTBIO. Total RNA of CRL-1474-Vector and CRL-1474-progerin (transfected with Vector or progerin for 9 days) cells was isolated using TRizol reagent. The RNA-seq libraries were constructed using a VAHTS Universal V6 RNA-seq Library Pren Kit (Vazyme) for Illumina and sequenced on an IlluminaHiSeq 2500 at Biomarker Technologies. All raw RNA-seq reads were mapped to the human genome (hg19) with TopHat coupled with Bowtie 2 and default parameters. Transcriptomes were assembled and fragments per kilobase per million reads for each gene were computed with Cufflinks. A cut-off *P* value <0.05 and absolute values of log_2_-fold changes greater than 1 were used for differential gene expression analysis.

### Western blot

Western blot was performed as previously described^54^. Cells were lysed in 1× Laemmli sample buffer after washing twice in cold PBS without calcium and magnesium. Protein concentrations were determined using the bicinchoninic acid (BCA) protein assay. Protein lysates were subjected to SDS–PAGE, transferred to 0.45-μm pore size hydrophobic PVDF transfer membrane (Merck Millipore), detected with the appropriate primary antibodies coupled with horseradish peroxidase-conjugated corresponding secondary antibodies, and visualized using enhanced chemiluminescence ECL reagent (GE Healthcare). The Tanon 5500 high-definition low-illumination CCD system (Tanon FLI Capture v.1.02) was used to capture chemiluminescent signals.

### Immunofluorescence

Immunofluorescence was performed as described previously^54^. Cells were plated on cover slips in 12-well plates, left overnight before the treatment. Cells were fixed in 1% formaldehyde in culture medium for 10 min at 37 °C and permeabilized with 0.2% Triton X-100 in PBS for 10 min at room temperature. Cells were washed twice in PBS and blocked for 1 h with 5% BSA in PBS and then incubated with primary antibodies at 4 °C overnight, washed three times in PBS and incubated with secondary antibodies for 1 h at room temperature. Cell nuclei were counterstained with a 500 nM concentration of 4,6-diamidino-2-phenylindole (DAPI) (Sigma). Images were acquired by a fluorescence microscope (OLYMPUS FV-1000, Carl Zeiss LSM880 or Leica DMi8) and analyzed with software NIS-Elements AR v.5.0.1 (Nikon), FLUOVIEW v.4.2b (OLYMPUS), ZEN v.2.3 SP1 (Carl Zeiss) and Leica Application Suite X v.3.6.0.24 (Leica). Cells were quantified by investigators who were blinded to the identity of the analyzed cell.

### Reagents and antibodies

All reagents were obtained from Sangon Biotech (Shanghai) except those listed as follows: Actinomycin D (Act D; Sigma, catalog no. A1410) was obtained from Sigma-Aldrich. Antibodies used in this study at the indicated dilution are as follows: anti-lamin A/C 1:3,000 (Abcam, catalog no. ab108595), anti-progerin (13A4) 1:1,000 (Abcam, catalog no. ab66587), anti-P-H3 (S10) 1:1,000 (Cell Signaling Technology (CST), catalog no. 3377), anti-H3K9me3 1:10,000 (Millipore, catalog no. 07-523), anti-H3K27me3 1:10,000 (Millipore, catalog no. 07-449), anti-BUBR1 1:1,000 for western blot and 1:500 for immunofluorescence (Abcam, catalog no. ab54894), anti-BUBR1 1:1,000 for western blot and 1:500 for immunofluorescence (Abcam catalog no. ab209998), anti-CDC20 1:1,000 (Abcam, catalog no. ab26483), anti-BUB3 1:1,000 (BD Biosciences, catalog no. BD611730), anti-CyclinA2 1:2,000 (Abcam, catalog no. ab181591), anti-PTBP1 1:1000 for western blot and 1:500 for immunofluorescence (Santa Cruz, catalog no. sc56701), anti-lamin B1 1:1,000 (Santa Cruz, catalog no. sc-6216), anti-Ki67 1:500 for immunofluorescence (GeneTex, catalog no. GTX16667), anti-Flag 1:3000 for western blot and 1:500 for immunofluorescence (Sigma-Aldrich, catalog no. F1804), anti-HA 1:2,000 (Sigma-Aldrich, catalog no. H9658), anti-GFP 1:2,000 for western blot (Sungene Biotech, catalog no. KM8009), anti-γH2AX 1:2,000 for western blot and 1:500 for immunofluorescence (CST, catalog no. 80312), anti-γH2AX 1:500 for immunofluorescence (CST, catalog no. 9718), anti-53BP1 1:500 for immunofluorescence (CST, catalog no. 4937), anti-IL-6 1:2,000 for western blot (Immunoway, catalog no. YT5348), anti-p21 1:2,000 for western blot (Proteintech, catalog no. 10355-1-AP), anti-p53 1:1,000 for western blot (Sigma-Aldrich, catalog no. P6749), Alexa Fluor 594 goat anti-mouse IgG (H+L) 1:500 for immunofluorescence (Invitrogen, catalog no. A21203), Alexa Fluor 594 goat anti-rabbit IgG (H+L) 1:500 for immunofluorescence (Invitrogen, catalog no. A11037), Alexa Fluor 488 goat anti-mouse IgG (H+L) 1:500 for immunofluorescence (Invitrogen, catalog no. A11029), Alexa Fluor 488 goat anti-rabbit IgG (H+L) 1:500 for immunofluorescence (Invitrogen, catalog no. A11008). Normal mouse IgG 1:1,000 (Santa Cruz Biotechnology, catalog no. sc-2025), normal rabbit IgG 1:1,000 (CST, catalog no. 2729), secondary goat anti-mouse (1:3,000), and goat anti-rabbit (1:2,000) antibodies were obtained from ZSGB-BIO.

### SA-β-gal staining

SA-β-gal staining was performed as previously described^54^. Cells were fixed in 0.5% glutaraldehyde for 15 min at room temperature, washed three times with PBS (pH 6.0) containing 1 mM MgCl_2_ and exposed overnight 37 °C to containing 1 mg ml^–1^ 5-bromo-4-chloro-3-indolyl-β-d-galactopyranoside (MCE, catalog no. HY-15934), 0.12 mM potassium ferrocyanide (Sigma, catalog no. P3289), 0.12 mM potassium ferricyanide (Sigma, catalog no. P8131) and 1 mM MgCl_2_ PBS buffer (pH 6.0). Cells were washed three times with H_2_O and observed under a microscope (OLYMPUS CKX53). SA-β-gal positive cells were quantified by investigators who were blinded to the identity of the analyzed cell.

### Coimmunoprecipitation

Detailed descriptions of CoIP were described previously^55,56^. Cells were harvested and washed three times in cold PBS, then lysed in CoIP buffer (25 mM Tris-HCl (pH 7.5), 150 mM KCl, 5 mM EDTA, and 0.5% NP-40) plus protease inhibitor cocktail tablet (Roche). Incubate on ice and shock lysates on the vortex shaker 15 times for 30 s each. Microcentrifuge in 12,000*g* for 15 min at 4 °C, collect the supernatant, and incubate with 3 μg primary antibodies with gentle shaking overnight at 4 °C, followed by adding 20–40 μl Pure Proteome Protein A/G Mix Magnetic Beads (Millipore, catalog no. LSKMAGAG02) for 3 h. Wash the beads three times with 1 ml CoIP buffer for 8 min each, resuspend in 50 μl 1× loading buffer, and incubate at 100 °C for 8 min. Throw beads away and analyze by immunoblotting.

### RIP assay and RNA pulldown assay

RIP and RNA pulldown assays were performed as previously described^57^. The RIP experiment was carried out using the EZ-Magna RIP Kit (Millipore, catalog no. 17-701). The coprecipitated RNAs were extracted through Trizol reagent and detected by RT-PCR. The primer sequences for PCR, synthesized by Comate Bioscience, were as follows:

BUBR1-3′UTR_fwd (5′-TACAAATGGTTACCTTGT-3′) and BUBR1-3′UTR_rev (5′- AAAATACATGGTGATCATAAGAGA-3′).

Flag-MS2bp-MS2bs-based RNA pulldown assay details: pcDNA3-Flag-MS2bp and pcDNA3-BUBR1-3′-UTR or pcDNA3-BUBR1-3′-UTR-ΔUCUU were cotransfected to HEK-293T cells, the cells were fixed in 3% formaldehyde for 10 min and terminated by glycine (125 mM) for 5 min after 48 h. The cells were harvested and lysed in soft lysis buffer (20 mM Tris-HCl (pH 8.0), 10 mM NaCl, 1 mM EDTA, 0.5% NP-40) with RNasin (80 U ml^–1^, Promega). Then, 50 μl ANTI-FLAG M2 Magnetic beads (Millipore) was added to each binding reaction tube and incubated overnight at 4 °C. The beads were washed five times with lysis buffer for 8 min each, resuspend in 50 μl 1× loading buffer, and incubated at 100 °C for 8 min. The beads were discarded and the sample analyzed by immunoblotting.

### MS analysis

Flag-tagged progerin was transfected into HEK-293T cells. PEI reagent was used according to the manufacturer’s instructions. The cells were lysed in buffer A mixed with protease inhibitor cocktail tablet (Roche) after 48 h; 30 μl anti-Flag affinity gel (Biotool, catalog no. B23102) was added and incubated for 16 h at 4 °C. The beads were washed three times using 1× Tris saline buffer (50 mM Tris-HCl (pH 7.4) and 150 mM NaCl)) for 5 min each. Flag-progerin protein was purified from HEK-293T cells, followed by SDS–PAGE analysis. The gel was stained by Coomassie Brilliant Blue. Progerin-binding proteins were analyzed by liquid chromatography–tandem MS performed at APTBIO.

### Metaphase chromosome spread assay

For the metaphase chromosome spread assay, 1.5 × 10^6^ cells were seeded in a 6 cm dish and incubated for 24 h with fresh medium. Then, colchicine was added to a final concentration of 0.2 μg ml^–1^ and the cells were incubated at 37 °C for 2 h. After digestion, we collected and rinsed the cells with PBS. The cells were centrifuged at 160*g* for 10 min. Then, 1 M KCl was added to a final concentration of 0.075 mol l^–1^ and the samples were incubated at 37 °C for 20 min. The cells were centrifuged, and the supernatant aspirated. The pellet was resuspended, and 1 ml methanol:glacial acetic acid (3:1) solution was added. The sample was rested at room temperature for 10 min. Then, the cells were centrifuged at 160*g* for 10 min. The supernatant was aspirated, and the pellet resuspended in 1 ml fixative for 30 min. The cells were centrifuged and the pellet was suspended in 1 ml fixative again for 30 min. The cells were centrifuged at 160*g* for 10 min and the supernatant aspirated. The pellet was then resuspended in a small volume of fixative (typically less than 500 μl) until the cell suspension appeared lightly milky. A small quantity of the cell suspension was held vertically in a pipette with the end about 10 cm above the slide. A single drop was released from this height onto the slide and allowed to air dry. The cells were then incubated with 500 nM of DAPI (Invitrogen) for 10 min. Slides were examined with an Olympus BX53 fluorescence microscope and the images were captured by using the Olympus IPP v.7.0 software. Aneuploid cells were quantified by investigators who were blinded to the identity of the analyzed cell.

### Luciferase reporter assay

The experiments were performed as described previously^58^. The BUBR1 (–1,200 to +100) promoter or the DNA fragment of *BUBR1* mRNA 3′-UTR was inserted into the pGL4.20 vector. Cells were plated in 24-well plates. After 24 h, cells were cotransfected with 200 ng of the indicated firefly luciferase reporter plasmid and 50 ng of Renilla luciferase normalization control plasmid using PEI. At 48 h after transfection, cell lysates were collected to measure luciferase activity using the Dual-Luciferase Reporter Assay System (Promega, catalog no. E1910) according to the manufacturer’s instructions. Luciferase reporter assay was carried out on an TD-20/20 Turner Designs luminometer, data were analyzed and further processed in Microsoft Excel and GraphPad Prism v.7.

### Cell viability assay

Cell viability was assessed using the Cell Counting Kit-8 (CCK8) (APE, catalog no. k1018). The cells were plated in triplicate in 96-well plates (typically 10,000 cells per 100 μl per well) and were incubated with fresh medium for 24 h. Then, the cells were incubated with different concentrations (0 μM, 10 μM, 20 μM, 30 μM) of UPCP for 0 h, 24 h, 48 h or 72 h. CCK8 solution (10 μl) was added to the well for a 2 h incubation at 37 °C. Absorbance was measured at 450 nm with a 96-well plate reader (Tecan Infinite M NANO^+^). Data were analyzed and further processed in i-control v.2.0 and GraphPad Prism v.7.

### Affinity purification of Flag-tagged protein

PCDH-Flag-BUBR1 or pWPXLd-Flag-PTBP1 plasmid was transfected into HEK-293T cells by using the PEI reagent according to the manufacturer’s instructions. Cells were harvested and lysed in buffer A mixed with protease inhibitor cocktail tablet (Roche) after 48 h. Then, 30 μl anti-Flag affinity gel (Biotool, catalog no. B23102) was added and incubated for 16 h at 4 °C. The beads were washed three times using 1× Tris saline buffer (50 mM Tris-HCl (pH 7.4) and 150 mM NaCl)) for 8 min each. The bound proteins BUBR1 or PTBP1 were eluted with 3×Flag peptide (ApexBio) for 2 h at 4 °C. Finally, BUBR1 or PTBP1 was concentrated and recovered by using the Centrifugal Filters (Amicon Ultra).

### Pulldown assay

A detailed description of the pulldown assay was described previously^56^. His-UPCP fusion protein was expression in bacteria (BL21) and induced with 0.5 mM isopropyl-β-d-thio-galactoside for 16 h at 16 °C. The bacteria were harvested and lysed in TEN buffer (20 mM Tris-HCl (pH7.4), 0.1mM EDTA, 100 mM NaCl). The supernatant was collected by centrifugation at 12,000*g* for 15 min at 4 °C, incubated with His-tag affinity gel (Elut-p008, Enriching Biotechnology LTD), and then washed three times with TEN buffer. His-UPCP was eluted with Imidazole eluent (50 mM NaH_2_PO_4_, 300 mM NaCl and 50 mM Imidazole) for 2 h at 4 °C and concentrated and recovered using centrifugal filters (Amicon Ultra). For the pulldown of His-UPCP and Flag-BUBR1 or Flag-PTBP1, 10 μg His-UPCP was incubated with Flag beads (Biotool, catalog no. B23102), Flag beads-BUBR1 or Flag beads-PTBP1 respectively for 16 h at 4 °C. The beads were washed three times with PBS and added to 1× loading buffer for western blot.

### Microscale thermophoresis assay

His fusion proteins (His-UPCP) were expressed in bacteria (BL21) and induced with 0.5 mM isopropyl-β-d-thio-galactoside for 16 h at 16 °C and purified by His-tag affinity gel (Ni-NTA). His-UPCP was eluted with Imidazole eluent for 2 h at 4 °C and concentrated and recovered by using centrifugal filters (Amicon Ultra). Flag-BUBR1 or PTBP1 was purified as described in Affinity purification of Flag-tagged protein. The purified protein was diluted with 0.05% Tween-20 in PBS in to an appropriate concentration (His-UPCP: 50 nM, Flag-BUBR1: 25 μM, Flag-PTBP1: 45 μM). Then, His-UPCP was labeled with RED-Tris-NAT according to the manufacturer’s instructions (NanoTemper, MO-L018). Flag-BUBR1 or Flag-PTBP1 was diluted by 16 concentration gradients in turn and mixed with labeled protein (His-UPCP). The capillaries (MST NT115 premium) were loaded and the samples measured on the instrument (NanoTemper Monolith NT.115). Data were analyzed by MO.AffinityAnalysis and curves were drawn using Origin 2019.

### Animal experiments

All animal experiments were approved by the Ethics Committee of School of Life Sciences, Northeast Normal University, China (AP20191011) and carried out in accordance with the relevant guidelines: National Standards of the People’s Republic of China (GB/T 35892–2018), Laboratory Animal—Guideline for Ethical Review of Animal Welfare. We generated a mouse model (ICR background), carrying the *Lmna*^*G609G*^ using the BE4-Gam system. The protocols for the BE4-Gam system and microinjection of pronuclear-stage embryos have been described in detail previously^37,38,59^. Briefly, a mixture of BE4-Gam mRNA (200 ng μl^–1^) and sgRNA-Lmna (30 ng μl^–1^) was coinjected into the cytoplasm of pronuclear-stage embryos of the mouse. The injected embryos were transferred to embryo culture medium for 30–60 min, followed by transferring the injected embryos into the recipient mother. The embryos continued to develop and the founder mice (referred to as F0) were born: wild-type mice (*Lmna*^*+/+*^), heterozygous mice (*Lmna*^*G609G/+*^) and homozygous mice (*Lmna*^*G609G/G609G*^). F1 heterozygous mice (*Lmna*^*G609G/+*^) were obtained by crossing F0 heterozygous mice (*Lmna*^*G609G/+*^). F2 homozygous mice (*Lmna*^*G609G/G609G*^) were obtained by crossing F1 heterozygous mice (*Lmna*^*G609G/+*^). We selected F2 mice (*Lmna*^*+/+*^ and *Lmna*^*G609G/G609G*^) for subsequent experiments. The mice were housed with a 12-h light/dark cycle (6:00 to 18:00) in a 20 °C and 40% humidity-controlled room. Water and rodent feed were fed ad libitum.

sgRNA-Lmna oligo sequences used: GTGGGCGGATCCATCTCCTC. Genotypes of newborn mice were identified using the Mouse Direct PCR Kit (Bimake, catalog no. B40015). Genotyping of mice For PCR genotyping the following primers were used: mLmna_fwd (5′-CGAAGGCTTCCTGGCTATTT-3′) and mLmna_rev (5′-TGCTGTAGGGCAGAGATGA-3′).

For UPCP injection, 14-day-old *Lmna*^*G609G/G609G*^ mice were injected intraperitoneally with either PBS or UPCP (20 mg kg^–1^, every other day) and wild-type mice were injected with PBS. All phenotypic analyses were performed after 12 weeks of treatment with UPCP. Bodyweight and lifespan were monitored after UPCP treatment. The survival rate was analyzed by the Kaplan–Meier method. We only included mice that were of sufficient bodyweight at the start of the experiment, typically at least 80% of the average littermate weight of the same sex and genotype. All animals meeting criteria for inclusion were then divided randomly into three groups according to genotype (*Lmna*^*+/+*^, *Lmna*^*G609G/G609G*^-Mock and *Lmna*^*G609G/G609G*^-UPCP).

### Histological analyses

For histological analyses, mice of the indicated genotypes were perfused with PBS, then with 4% paraformaldehyde (PFA). Whole organs were incubated in 4% PFA at 4 °C for 16 h. All samples were paraffin embedded and sectioning by Servicebio. Subsequently, the tissues were deparaffinized and rehydrated before hematoxylin-eosin (HE) or Masson staining or immunohistochemistry (IHC). HE staining was performed using an HE Kit (Absin, catalog no. abs9217), and Masson staining was performed using Masson’s Trichrome Stain Kit (Absin, catalog no. abs9347) according to the manufacturer’s instructions. The thickness of the layers or the areas of fibrosis in the tissues were calculated using ImageJ software by a blinded investigator. For IHC, the sections were completed by the Servicebio. The images were acquired by a microscope (Leica DMi8) and analyzed with Leica Application Suite X v.3.6.0.24 (Leica). For BubR1, cytoplasmic staining in cells was scored on the intensity of staining and the proportion of positive cells by a blinded investigator. The intensity of the staining was graded as 0 (negative, no color), 1 (low positive, light yellow), 2 (positive, brown yellow) or 3 (high positive, brown), and the number of positive cells was graded as 0 (<5%), 1 (5–25%), 2 (25–50%), 3 (51–75%) or 4 (>75%). The final score was defined as staining number score multiplied by staining color score. For lamin B1, p21 or IL-6, the proportion of positive cells was used to quantify the expression of protein in tissues by a blinded investigator.

### Open field assays

The mice were acclimated to the testing room for 2 h before the test. Before each individual trial, the testing apparatus was cleaned with 75% ethanol. Mice were placed individually in a random corner of the box (40 × 40 × 35 cm) facing the wall. Their movements, indicating locomotor activity, were recorded with a video camera for 20 min and were analyzed with Ethovision XT 10 (Noldus).

### Statistics and reproducibility

The results were compiled from at last three independent replicate experiments and are presented as mean ± s.d. Statistical parameters and methods are reported in the figure legends. Unless specified, the unpaired Student’s *t*-test (two-tailed) or two-way analysis of variance (ANOVA) was used to calculate the significance of differences between groups. Data were considered significant at *P* < 0.05 and the exact *P* values are indicated on each plot. Data distribution was assumed to be normal but this was not formally tested. Statistical analysis was conducted using GraphPad Prism v.7 software (GraphPad Software). For mouse experiments, investigators performing endpoint analyses were blinded to the treatment group. No statistical methods were used to predetermine sample sizes but our sample sizes are similar to those reported in previous publications^47–50,59^. Data distribution was assumed to be normal but this was not formally tested. All animals meeting criteria for inclusion were divided randomly into three groups according to genotype (*Lmna*^*+/+*^, *Lmna*^*G609G/G609G*^-Mock and *Lmna*^*G609G/G609G*^-UPCP). The tissues of mice in three groups were harvested for histological analysis by a blinded investigator. No animals or datapoints were excluded from the analyses in the study.

### Reporting summary

Further information on research design is available in the Nature Portfolio Reporting Summary linked to this article.

## Supplementary information

Supplementary Information Supplementary Data Figs. 1 and 2.

Reporting Summary

Supplementary Data 1 MS data of progerin-binding proteins.

## Data Availability

Any data and materials that can be shared will be released via a Data/Material sharing Agreement. All requests should be made to the primary or corresponding authors. RNA-seq data generated in the present study was deposited in the NCBI SRA (no. PRJNA817844)^59^. The MS proteomics data have been deposited to the ProteomeXchange Consortium via the PRIDE partner repository with the dataset identifier PXD039136. Source data are provided with this paper.

## Source data

Source Data Fig. 1 Statistical Source Data.
Source Data Fig. 2 Statistical Source Data.
Source Data Fig. 3 Statistical Source Data.
Source Data Fig. 4 Statistical Source Data.
Source Data Fig. 5 Statistical Source Data.
Source Data Fig. 6 Statistical Source Data.
Source Data Fig. 7 Statistical Source Data.
Source Data Extended Data Fig. 1 Statistical Source Data.
Source Data Extended Data Fig. 2 Statistical Source Data.
Source Data Extended Data Fig. 3 Statistical Source Data.
Source Data Extended Data Fig. 5 Statistical Source Data.
Source Data Extended Data Fig. 6 Statistical Source Data.
Source Data Extended Data Fig. 7 Statistical Source Data.
Source Data Extended Data Fig. 8 Statistical Source Data.
Source Data Extended Data Fig. 10 Statistical Source Data.
Source Data Fig. 1 Unprocessed western blots and/or gels.
Source Data Fig. 2 Unprocessed western blots and/or gels.
Source Data Fig. 3 Unprocessed western blots and/or gels.
Source Data Fig. 4 Unprocessed western blots and/or gels.
Source Data Fig. 5 Unprocessed western blots and/or gels.
Source Data Fig. 6 Unprocessed western blots and/or gels.
Source Data Fig. 7 Unprocessed western blots and/or gels.
Source Data Extended Data Fig. 1 Unprocessed western blots and/or gels.
Source Data Extended Data Fig. 2 Unprocessed western blots and/or gels.
Source Data Extended Data Fig. 3 Unprocessed western blots and/or gels.
Source Data Extended Data Fig. 4 Unprocessed western blots and/or gels.
Source Data Extended Data Fig. 5 Unprocessed western blots and/or gels.
Source Data Extended Data Fig. 6 Unprocessed western blots and/or gels.
Source Data Extended Data Fig. 7 Unprocessed western blots and/or gels.
Source Data Extended Data Fig. 8 Unprocessed western blots and/or gels.
Source Data Extended Data Fig. 9 Unprocessed western blots and/or gels.
Source Data Extended Data Fig. 10 Unprocessed western blots and/or gels.
