# Author Correction: Unique progerin C-terminal peptide ameliorates Hutchinson–Gilford progeria syndrome phenotype by rescuing BUBR1

**DOI:** 10.1038/s43587-023-00427-9

**Published:** 2023-05-02

**Authors:** Na Zhang, Qianying Hu, Tingting Sui, Lu Fu, Xinglin Zhang, Yu Wang, Xiaojuan Zhu, Baiqu Huang, Jun Lu, Zhanjun Li, Yu Zhang

**Affiliations:** 1 Key Laboratory of Molecular Epigenetics of Ministry of Education (MOE), Northeast Normal University, Changchun, China; 2 Key Laboratory of Zoonosis Research, Ministry of Education, College of Animal Science, Jilin University, Changchun, China; 3 Institute of Genetics and Cytology, Northeast Normal University, Changchun, China

**Keywords:** Senescence, Mechanisms of disease, Cell therapies, Ageing

Correction to: *Nature Aging* 10.1038/s43587-023-00361-w. Published online 2 February 2023.

In the version of this article initially published, in Figure 7, the authors misidentified esophageal tissue and esophageal lamina propria as aortic tissue and aortic adventitia, respectively. Labels that said “aorta” have been replaced with “esophagus” in panels a, e and f, and the label “Aortic adventitia” has been replaced with “Lamina propria” in panel f. Corresponding text in the figure legend and in the Results section on page 190 has been updated to reflect this change. The errors have been corrected in the HTML and PDF versions of the article.

