# Supplementary material for: Unique progerin C-terminal peptide ameliorates Hutchinson–Gilford progeria syndrome phenotype by rescuing BUBR1
Source: Nat Aging. 2023 Feb 2;3(2):185–201. doi: 10.1038/s43587-023-00361-w (PMC10154249; doi:10.1038/s43587-023-00361-w)
Supplement: Supplementary file 1 — Supplementary Data Figs. 1 and 2. [file 43587_2023_361_MOESM1_ESM.pdf]

nature aging

Article https://doi.org/10.1038/s43587-023-00361-w

Unique progerin C-terminal peptide ameliorates Hutchinson–Gilford progeria syndrome phenotype by rescuing BUBR1

In the format provided by the authors and unedited

Unique progerin-C terminal peptide ameliorates Hutchinson–Gilford progeria syndrome phenotype by rescuing BUBR1

Na Zhang[1#], Qianying Hu[1#], Tingting Sui[2], Lu Fu[3], Xinglin Zhang[1], Yu Wang[3], Xiaojuan Zhu[1], Baiqu Huang[1], Jun Lu[3,*], Zhanjun Li[2,*] and Yu Zhang[1,*]

[*]Corresponding Author: Jun Lu, luj809@nenu.edu.cn; Zhanjun Li, lizj_1998@jlu.edu.cn; Yu Zhang, zhangy288@nenu.edu.cn.

SUPPLEMENTARY FIGURES

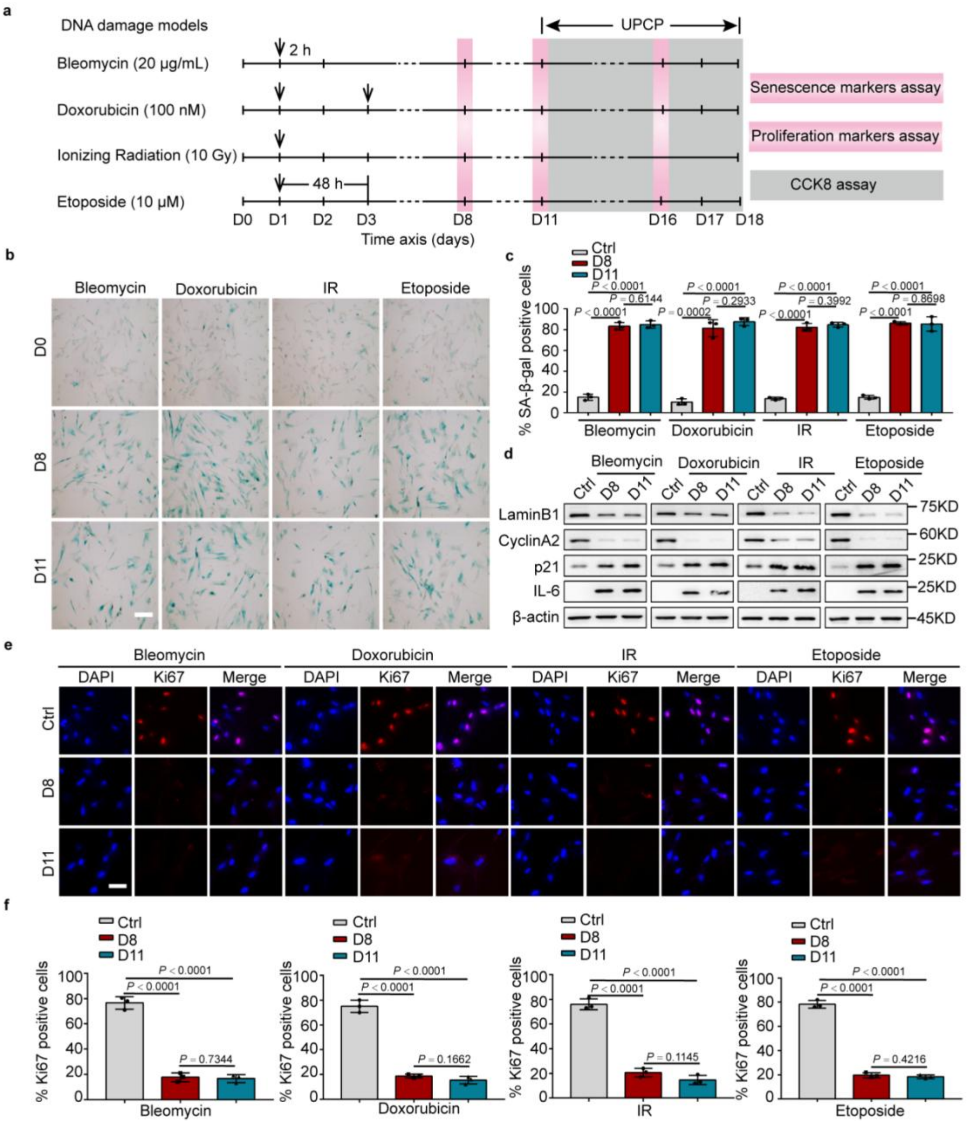

Supplementary Data Figure 1. DNA damage induces IMR90 cellular senescence. a. Schematic diagram of the time-axis for different DNA damage strategies [Bleomycin (20 μg/mL for 2 h), Doxorubicin (100 nM treatment twice for a 2 days interval), Ionizing Radiation (IR) (10 Gy Gamma rays) and Etoposide (10 μM for 48 h)]. **b,c.** β-Gal staining analysis of IMR90 cells with the indicated treatments. Representative images were showed (b) and the percentage of

SA-β-gal positive cells was calculated (c). Scale bars: 200 μm. **d.** Western blot analysis of Lamin B1, CyclinA2, IL-6 and p21 in senescent IMR90 cells with the indicated treatments. **e,f.** Immunofluorescence analysis of Ki67 in IMR90 cells with the indicated treatment. Representative images of Ki67 were showed (e) and the percentage of the Ki67 positive cells was calculated (f), n ≥ 200 cells. Scale bars: 50 μm. For bar and line graphs, data are presented as mean ± SD. Statistical significance was determined in **c** and **f** using two-tailed unpaired Student's t-test.

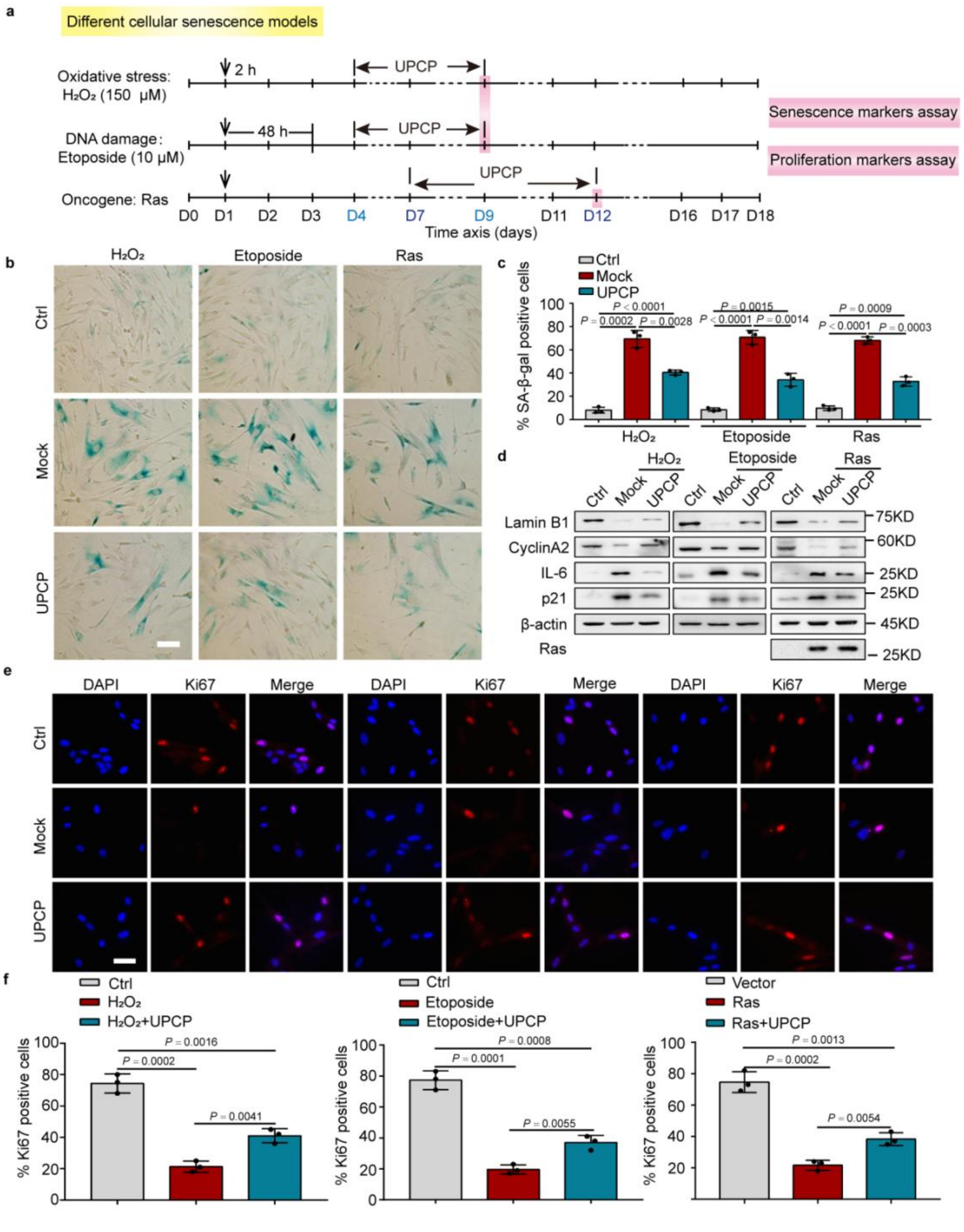

Supplementary Data Figure 2. UPCP delays Etoposide, Ras and H_2O_2 induced early stage cellular senescence. a. Schematic diagram of the time-axis for H_2O_2 (150 μM for 2 h), Etoposide (10 μM for 48 h) and Ras (expression for 7 days) induced cellular senescence. **b,c.** β-Gal staining analysis of IMR90 cells with the indicated treatments. Representative images of SA-β-Gal positive cells were showed (b) and the percentage of the SA-β-Gal positive cells was calculated (c). Scale

bars: 100 μm. **d.** Western blot analysis of Lamin B1, CyclinA2, Il-6, p21 and Ras in senescent IMR90 cells-induced by DNA damage Oxidative stress (H_2O_2), (Etoposide), or Oncogene (Ras) with or without UPCP (6 μM) treatment for another 5 days. **e,f.** Immunofluorescence analysis of Ki67 in IMR90 cells with the indicated treatment. Representative images of Ki67 were showed (e) and the percentage of the Ki67 positive cells was calculated (f), $n \geq 200$ cells. Scale bars: 50 μm. For bar and line graphs, data are presented as mean ± SD. Statistical significance was determined in **c** and **f** using two-tailed unpaired Student's t-test.
