# Supplementary material for: Unique progerin C-terminal peptide ameliorates Hutchinson–Gilford progeria syndrome phenotype by rescuing BUBR1
Source: Nat Aging. 2023 Feb 2;3(2):185–201. doi: 10.1038/s43587-023-00361-w (PMC10154249; doi:10.1038/s43587-023-00361-w)
Supplement: Supplementary file 2 — Reporting Summary [file 43587_2023_361_MOESM2_ESM.pdf]

nature portfolio

Corresponding author(s): Yu Zhang

Last updated by author(s): Nov 18, 2022

Reporting Summary

Nature Portfolio wishes to improve the reproducibility of the work that we publish. This form provides structure for consistency and transparency in reporting. For further information on Nature Portfolio policies, see our Editorial Policies and the Editorial Policy Checklist.

Statistics

For all statistical analyses, confirm that the following items are present in the figure legend, table legend, main text, or Methods section.

n/a	Confirmed	
☐	☒	The exact sample size (*n*) for each experimental group/condition, given as a discrete number and unit of measurement
☐	☒	A statement on whether measurements were taken from distinct samples or whether the same sample was measured repeatedly
☐	☒	The statistical test(s) used AND whether they are one- or two-sided<br>*Only common tests should be described solely by name; describe more complex techniques in the Methods section.*
☐	☒	A description of all covariates tested
☒	☐	A description of any assumptions or corrections, such as tests of normality and adjustment for multiple comparisons
☐	☒	A full description of the statistical parameters including central tendency (e.g. means) or other basic estimates (e.g. regression coefficient) AND variation (e.g. standard deviation) or associated estimates of uncertainty (e.g. confidence intervals)
☐	☒	For null hypothesis testing, the test statistic (e.g. *F*, *t*, *r*) with confidence intervals, effect sizes, degrees of freedom and *P* value noted<br>*Give P values as exact values whenever suitable.*
☒	☐	For Bayesian analysis, information on the choice of priors and Markov chain Monte Carlo settings
☒	☐	For hierarchical and complex designs, identification of the appropriate level for tests and full reporting of outcomes
☒	☐	Estimates of effect sizes (e.g. Cohen's *d*, Pearson's *r*), indicating how they were calculated

Our web collection on statistics for biologists contains articles on many of the points above.

Software and code

Policy information about availability of computer code

Data collection	-Olympus IPP software v. 7.0 was used for collecting the images of metaphase chromosome spread assay.<br>-QuantStuidoTM Design and Analysis Software v. 1.4.2 were used for collecting of the RT-PCR data.<br>-Tanon FLI Capture v 1.02 was used for Western blot.<br>-i-control v.2.0 was used for Cell viability assays.<br>- NIS-Elements AR 5.0.1(Nikon) , ZEN 2.3 SP1 (Carl Zeiss), FLUOVIEW Ver. 4.2b (OLYMPUS) and Leica Application Suite X 3.6.0.24(Lecia) were used for the acquirement and analysis of Images.<br>- Ethovision XT v. 10 was used for Open field assays.<br>-Nano Temper Monolith NT.115 was used for Microscale Thermophoresis assay.
Data analysis	-GraphPad Prism v. 7 was used for the statistical analysis.<br>-ImageJ v. 1.52a was used for calculating the thickness of the layers or the areas of fibrosis in the tissue or analyzing immunofluorescence signal intensity .<br>-SnapGene v. 5.0.5 was used for the genotypes analysis.<br>-Adobe Illustrator v. 21.0.0 was used for figures reorganization and model drawing.<br>-MO.AffinityAnalysis v2.2.4 was used for analysis and evaluation of MicroScale Thermophoresis data.<br>-Origin 2019 was used for drawing the curves of MicroScale Thermophoresis data.

For manuscripts utilizing custom algorithms or software that are central to the research but not yet described in published literature, software must be made available to editors and reviewers. We strongly encourage code deposition in a community repository (e.g. GitHub). See the Nature Portfolio guidelines for submitting code & software for further information.

Data

Policy information about availability of data

All manuscripts must include a data availability statement. This statement should provide the following information, where applicable:

- Accession codes, unique identifiers, or web links for publicly available datasets
- A description of any restrictions on data availability
- For clinical datasets or third party data, please ensure that the statement adheres to our policy

Any data and materials that can be shared will be released via a Data/Material sharing Agreement. All requests should be made to the primary or corresponding authors. RNA-seq data generated in the present study was deposited in the NCBI SRA (no. PRJNA817844).

Field-specific reporting

Please select the one below that is the best fit for your research. If you are not sure, read the appropriate sections before making your selection.

☒ Life sciences ☐ Behavioural & social sciences ☐ Ecological, evolutionary & environmental sciences

For a reference copy of the document with all sections, see nature.com/documents/nr-reporting-summary-flat.pdf

Life sciences study design

All studies must disclose on these points even when the disclosure is negative.

Sample size	No statistical methods were used to pre-determine sample sizes but our sample sizes was determined based on previously published reports (Koblan et al., Nature., 2021; Beyret et al., Nat Med., 2019; Santiago-Fernández et al. Nat Med., 2019； Chen et al., ELife., 2021; Hu et al., EMBO J., 2022).
Data exclusions	No data was excluded.
Replication	In vitro data was generated from technical triplicates performed on biological triplicate samples. in vivo data was generated from technical triplicate measurements of individual samples. All replicates were successful.
Randomization	All samples and animals used in this study were randomized into experimental groups.
Blinding	Ki67 positive cells, SA-β-gal positive cells, CyclinA2 positive cells, γH2AX or 53BP1 foci number, BUBR1 or PTBP1 nuclear membrane localization cells, aneuploid cells, chromosome laggings cells and abnormal spindles cells were quantified by investigators who were blinded to the identity of the analyzed cell. In the same way, for histological analysis (HE staining, Masson staining and immunohistochemistry) were also performed by investigators blinded to group identity.

Reporting for specific materials, systems and methods

We require information from authors about some types of materials, experimental systems and methods used in many studies. Here, indicate whether each material, system or method listed is relevant to your study. If you are not sure if a list item applies to your research, read the appropriate section before selecting a response.

Materials & experimental systems

n/a	Involved in the study
☐	☒ Antibodies
☐	☒ Eukaryotic cell lines
☒	☐ Palaeontology and archaeology
☐	☒ Animals and other organisms
☒	☐ Human research participants
☒	☐ Clinical data
☒	☐ Dual use research of concern

Methods

n/a	Involved in the study
☒	☐ ChIP-seq
☒	☐ Flow cytometry
☒	☐ MRI-based neuroimaging

Antibodies

Antibodies used	Anti-LaminA/C 1:3000 (Abcam, ab108595), Anti-progerin (13A4) 1:1000 (Abcam, ab66587), Anti-P-H3 (S10) 1:1000 (Cell Signaling Technology, #3377), Anti-H3K9me3 1:10000 (Millipore, #07-523), Anti-H3K27me3 1:10000 (Millipore, #07-449), Anti-BUBR1 1:1000 for western blot and 1:500 for immunofluorescence (Abcam, ab54894), Anti-BUBR1 1:1000 for western blot and 1:500 for immunofluorescence (Abcam ab209998), Anti-CDC20 1:1000 (Abcam, ab26483), Anti-BUB3 1:1000 (BD Biosciences, BD611730), Anti-CyclinA2 1:2000 (Abcam, ab181591), Anti-PTBP1 1:1000 for western blot and 1:500 for immunofluorescence (Santa Cruz, sc56701), Anti-Lamin B1 1:1000 (Santa Cruz, sc-6216), Anti-Ki67 1:500 for immunofluorescence (GeneTex, GTX16667), Anti-Flag

1:3000 for western blot and 1:500 for immunofluorescence (Sigma-Aldrich, F1804), Anti-HA 1:2000 (Sigma-Aldrich, H9658), Anti-γH2AX 1：2000 for western blot and 1:500 for immunofluorescence (Cell Signaling Technology, #80312), Anti-GFP 1:2000 for western blot (Sungene Biotech, KM8009),Anti-γH2AX 1：500 for immunofluorescence (Cell Signaling Technology, #9718), Anti-53BP1 1:500 for immunofluorescence (Cell Signaling Technology, #4937), Anti-IL6 1:2000 for western blot (Immunoway, YT5348), Anti-p21 1:2000 for western blot (Proteintech, 10355-1-AP), Anti-p53 1：1000 for western blot (Sigma-Aldrich, P6749). Normal mouse IgG 1:1000 (Santa Cruz Biotechnology, sc-2025), normal rabbit IgG 1:1000 (CST, #2729), secondary goat anti-mouse (1:3000), and goat anti-rabbit (1:2000) antibodies were obtained from ZSGB-BIO (Beijing, China).

Validation

All antibodies were validated based on manufacturer's statement with regards to either cellular localization or expected band in Western blot. All antibodies gave the correct localization pattern and gave the expected bands in whole cell extracts of human cells and mouse cells.

Eukaryotic cell lines

Policy information about cell lines

Cell line source(s)

Cell lines (IMR90, CRL-1474, HEK-293T, NIH-3T3 and H1299) were obtained from the American Type Culture Collection (ATCC, Manassas, VA, USA). Fibroblasts from patients with HGPS were obtained from The Progeria Research Foundation Cell and Tissue Bank (http://www.progeriaresearch.org). The following fibroblasts were used: HGADFN003 (2 years old), HGADFN167 (8 years old), HGMDFN090 (37 years old), HGFDFN168 (40 years old). Human dermal fibroblast (GM00038, 9 years old and AG09602, 92 years old) were obtained from Coriell Cell Repository (https://catalog.coriell.org).

Authentication

Fibroblasts from patients with HGPS (HGADFN003, HGADFN167) or healthy individuals (HGMDFN090 and HGFDFN 168) were authenticated by the Progeria Research Foundation. Human dermal fibroblasts (GM00038 and AG09602) were authenticated by the Coriell Cell Repository with appropriate authentication. Cell lines (IMR90, CRL-1474, HEK-293T, NIH-3T3 and H1299) were authenticated by the distributors.

Mycoplasma contamination

Cell lines are routinely tested for mycoplasma.

Commonly misidentified lines
(See ICLAC register)

No commonly misidentified cell lines were used in this study.

Animals and other organisms

Policy information about studies involving animals; ARRIVE guidelines recommended for reporting animal research

Laboratory animals

HGPS LmnaG609G/G609G mouse model (carrying the LmnaG609G by using the BE4-Gam system) was used in a ICR background. Both males and females were used in this study. Experiments were initiated at 2 weeks up to 20 weeks of age. Animals were housed in a 12 h light and dark cycle at a 20 degrees centigrade and 40% humidity-controlled room.

Wild animals

No wild animals were used in the study.

Field-collected samples

The study did not involve animals collected from the field.

Ethics oversight

All animal experiments were approved by the Ethics Committee of School of Life Sciences, Northeast Normal University, China (AP20191011).

Note that full information on the approval of the study protocol must also be provided in the manuscript.
