# Supplementary material for: Unique progerin C-terminal peptide ameliorates Hutchinson–Gilford progeria syndrome phenotype by rescuing BUBR1
Source: Nat Aging. 2023 Feb 2;3(2):185–201. doi: 10.1038/s43587-023-00361-w (PMC10154249; doi:10.1038/s43587-023-00361-w)

Figure 1c. Full length images of immunoblots.

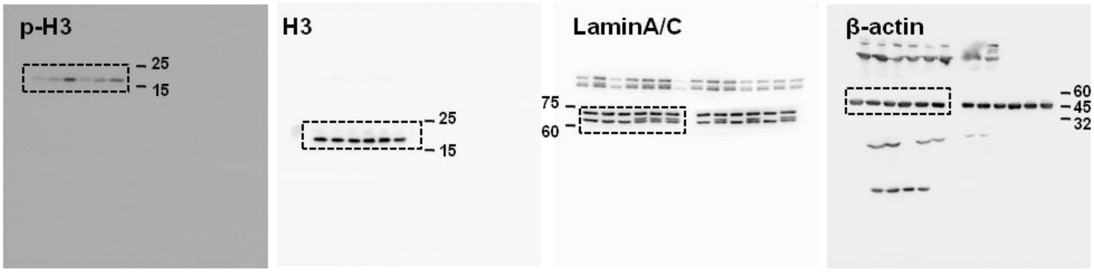

Figure 1d. Full length images of immunoblots.

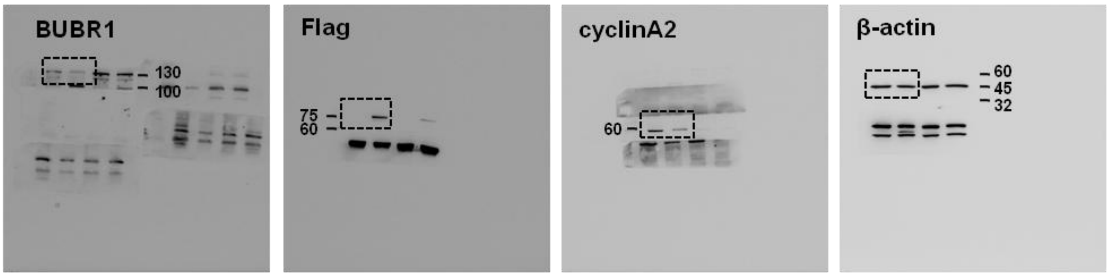

Figure 1d. Full length images of immunoblots.

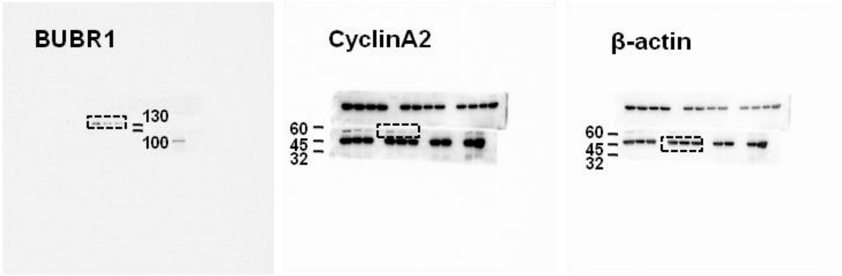

Figure 1h. Full length images of immunoblots.

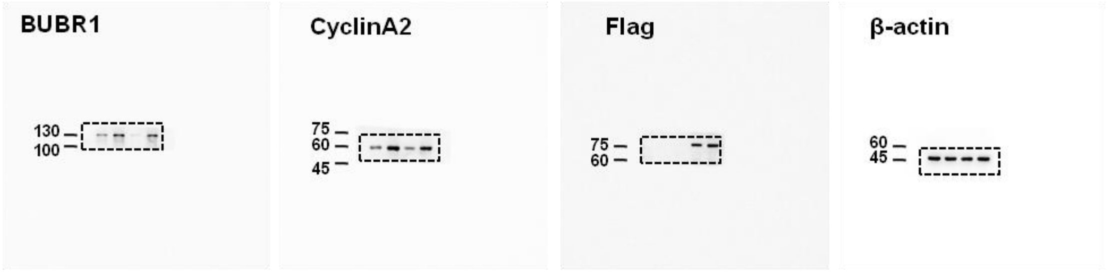

Figure 1j. Images of Immunofluorescence.

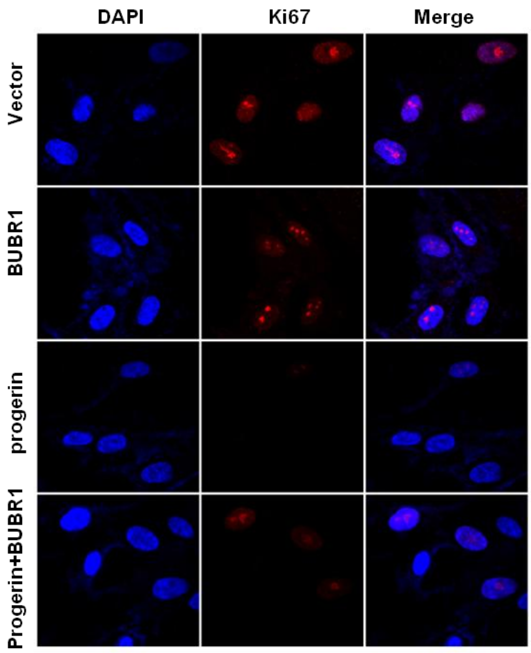

Figure 1l. Full length images of immunoblots.

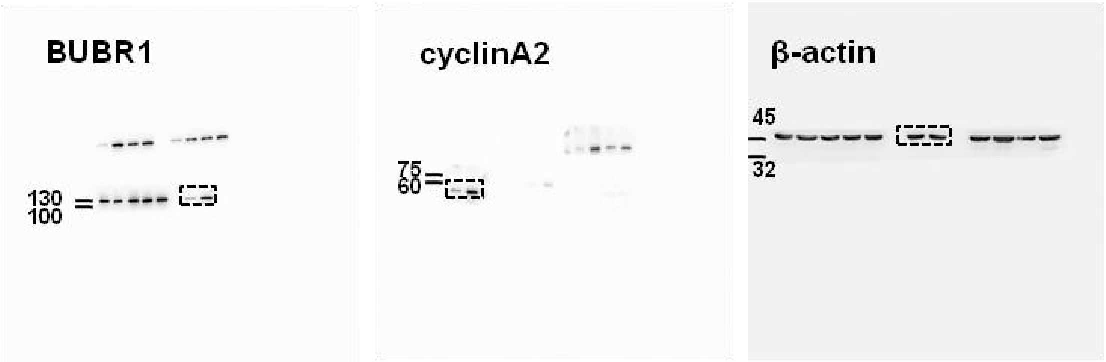

Figure 1n. Images of Immunofluorescence.

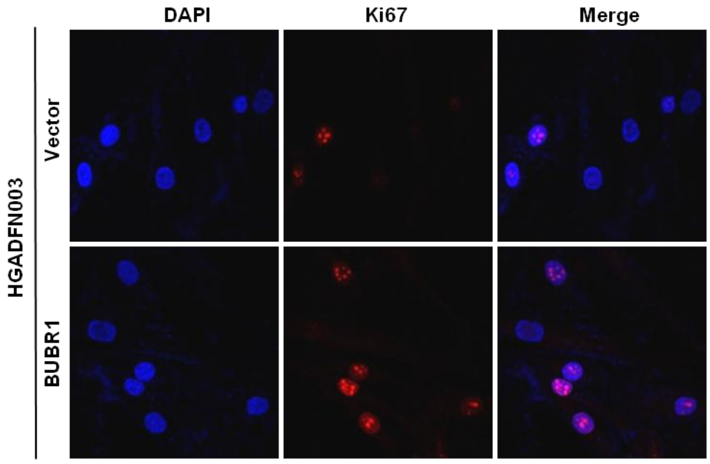

Supplement: Source Data Fig. 1 — Unprocessed western blots and/or gels. [file 43587_2023_361_MOESM19_ESM.pdf]
