# Supplementary material for: Unique progerin C-terminal peptide ameliorates Hutchinson–Gilford progeria syndrome phenotype by rescuing BUBR1
Source: Nat Aging. 2023 Feb 2;3(2):185–201. doi: 10.1038/s43587-023-00361-w (PMC10154249; doi:10.1038/s43587-023-00361-w)

Figure 2a. Images of Immunofluorescence.

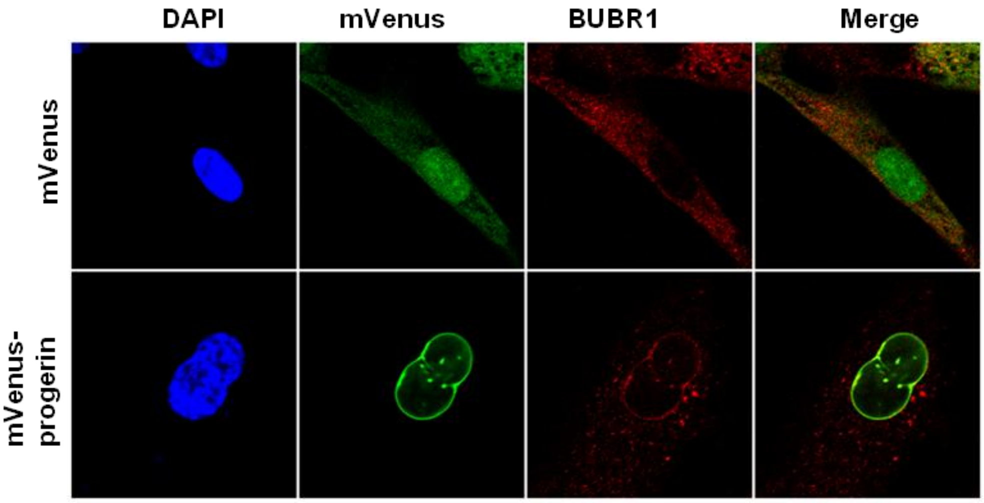

Figure 2d. Full length images of immunoblots.

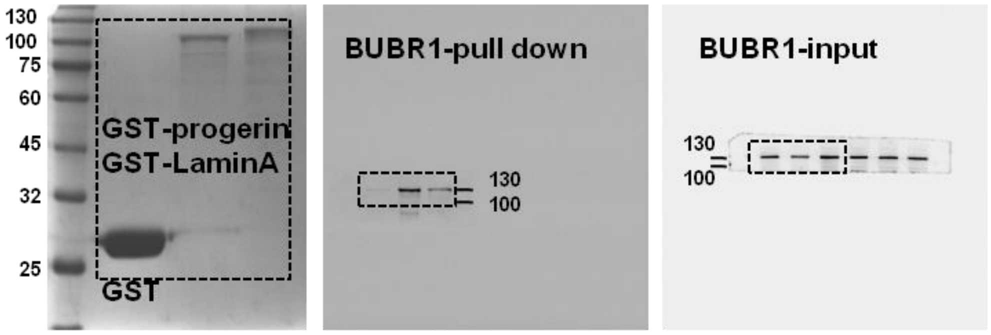

Figure 2e. Full length images of immunoblots.

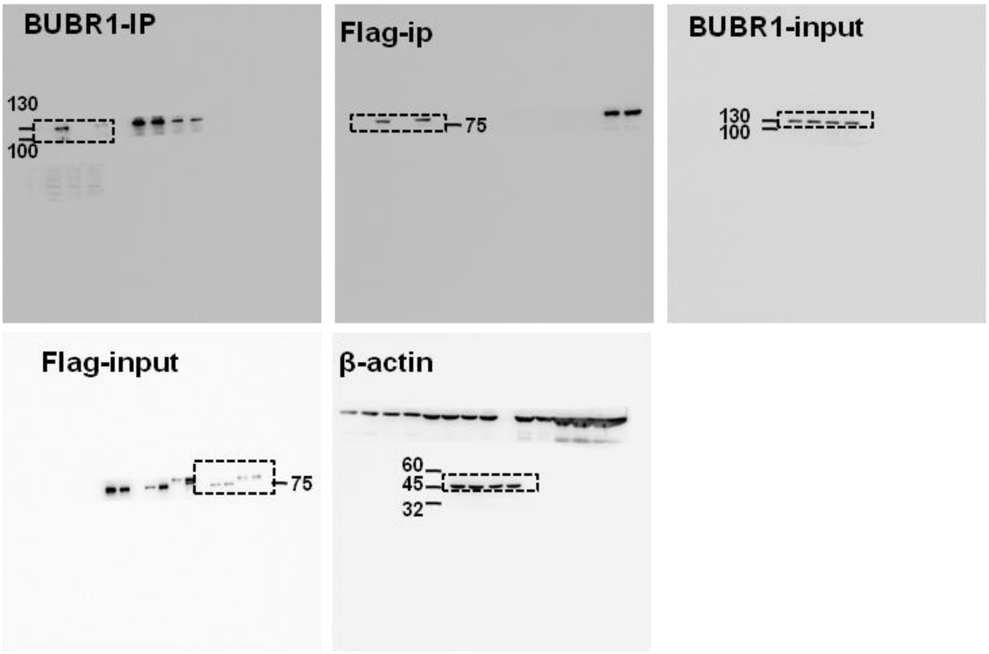

Figure 2g. Full length images of immunoblots.

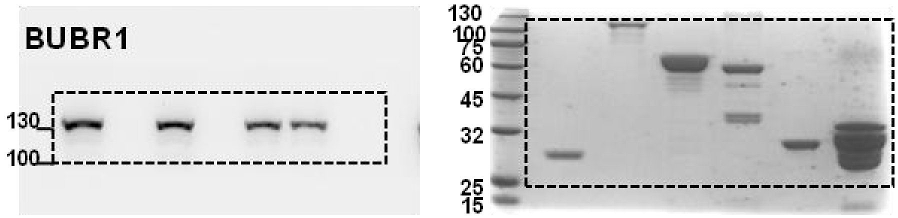

Figure 2h. Full length images of immunoblots.

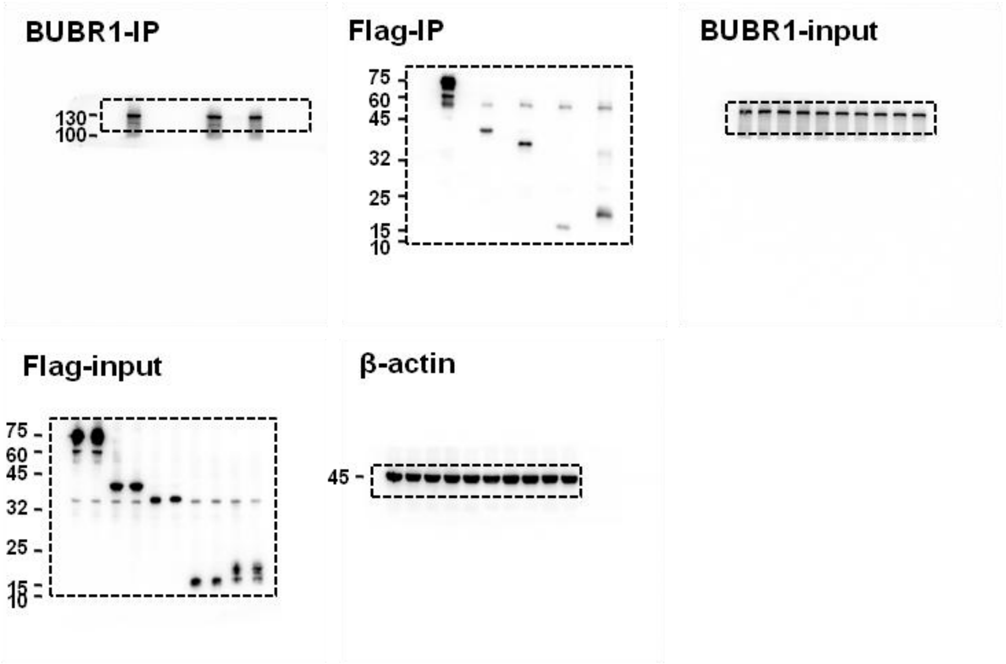

Figure 2j. Full length images of immunoblots.

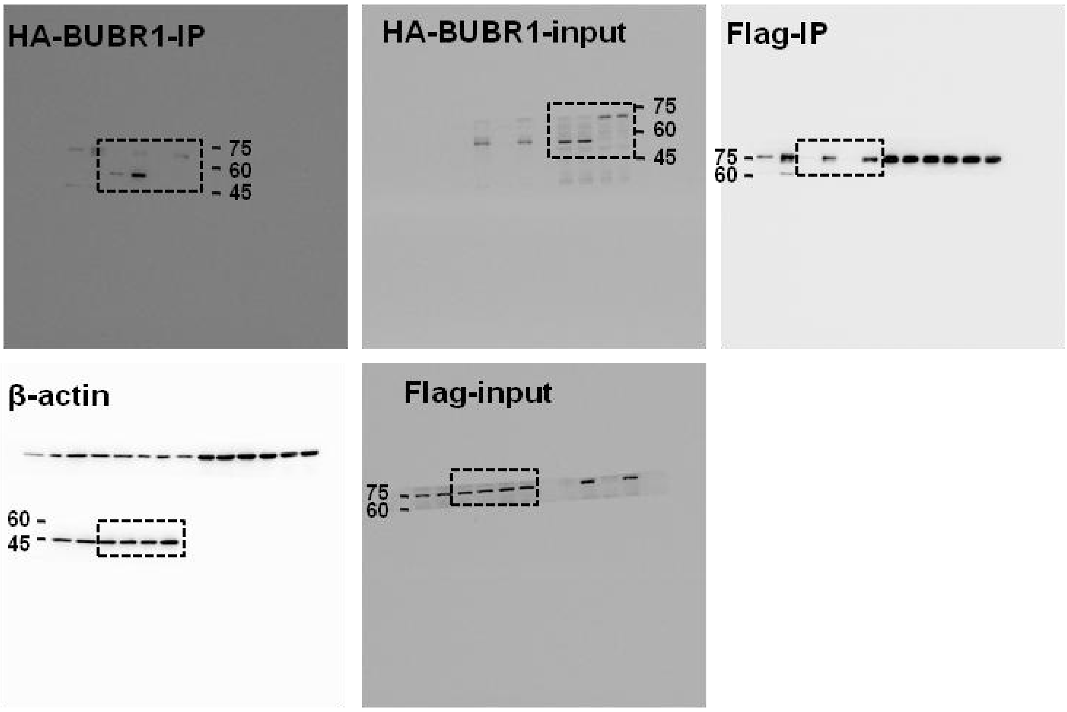

Figure 2k. Images of Immunofluorescence.

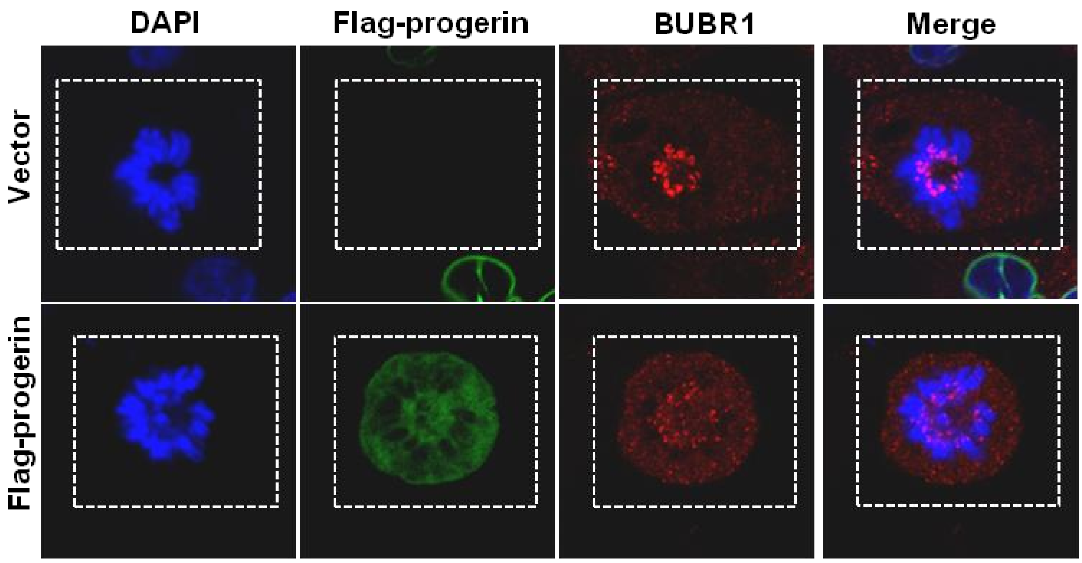

Figure 2m. Full length images of immunoblots.

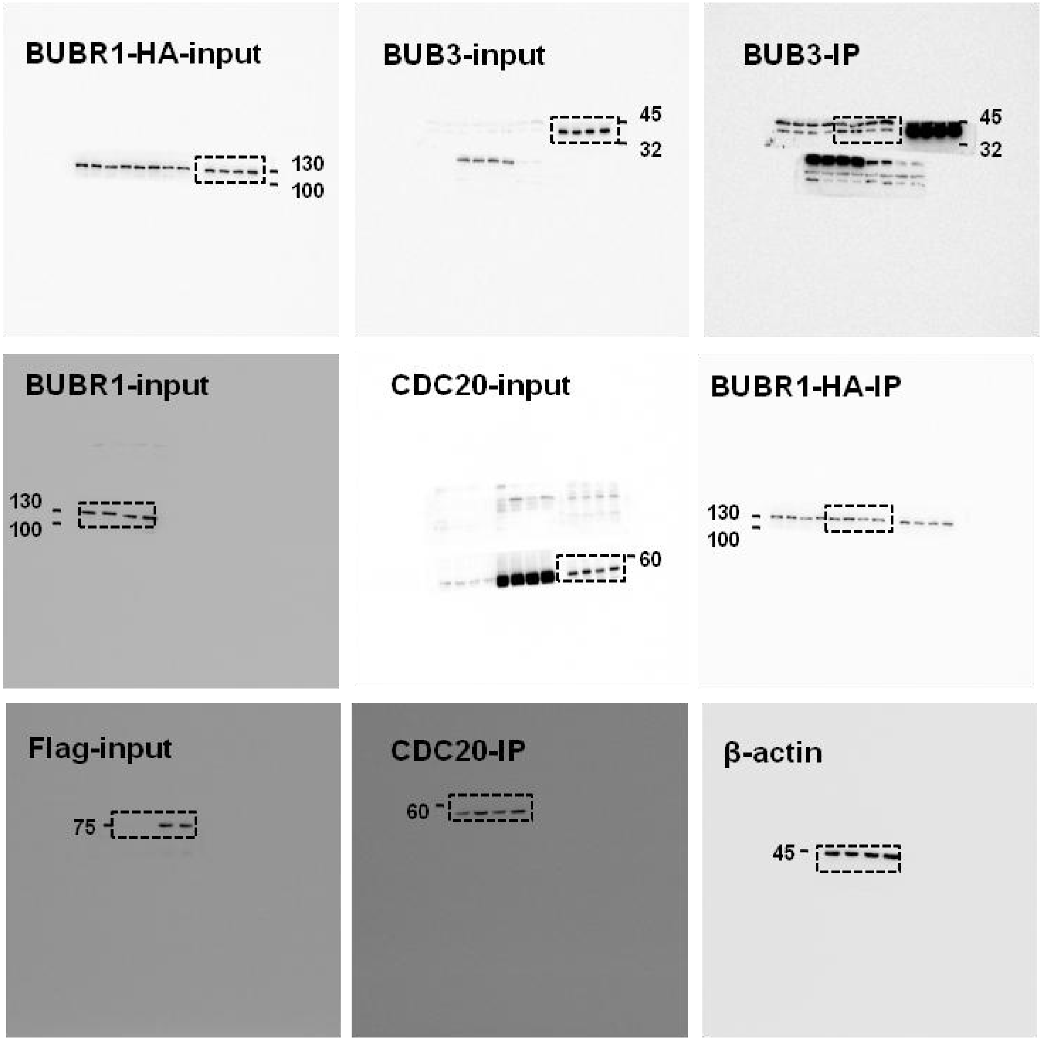

Supplement: Source Data Fig. 2 — Unprocessed western blots and/or gels. [file 43587_2023_361_MOESM20_ESM.pdf]
