# Supplementary material for: Unique progerin C-terminal peptide ameliorates Hutchinson–Gilford progeria syndrome phenotype by rescuing BUBR1
Source: Nat Aging. 2023 Feb 2;3(2):185–201. doi: 10.1038/s43587-023-00361-w (PMC10154249; doi:10.1038/s43587-023-00361-w)

Figure 3a. Full length images of immunoblots.

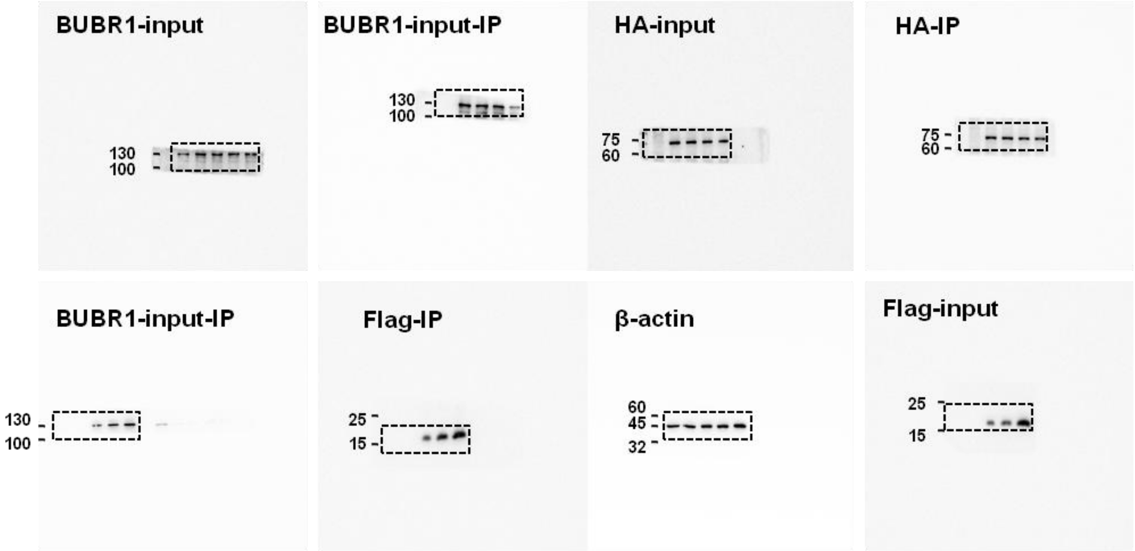

Figure 3c. Full length images of immunoblots.

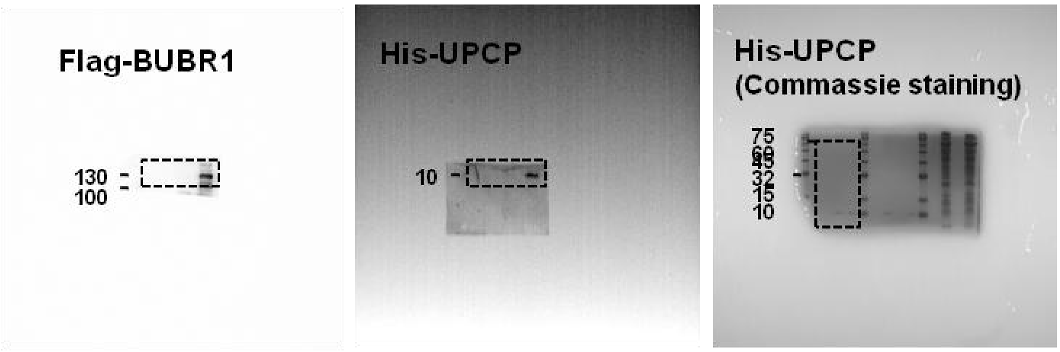

Figure 3e. Images of Immunofluorescence.

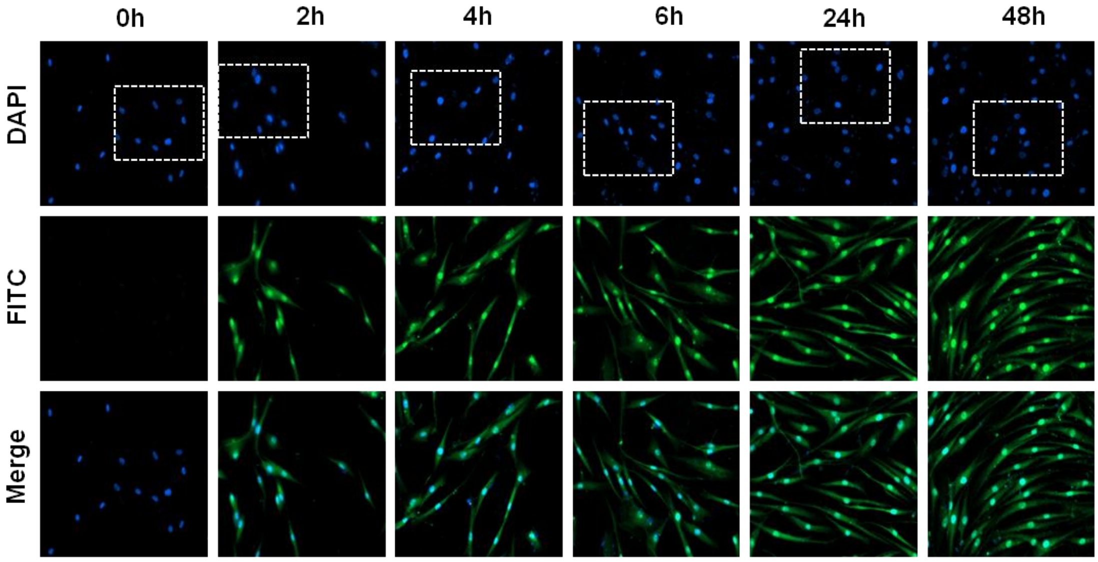

Figure 3f. Full length images of immunoblots.

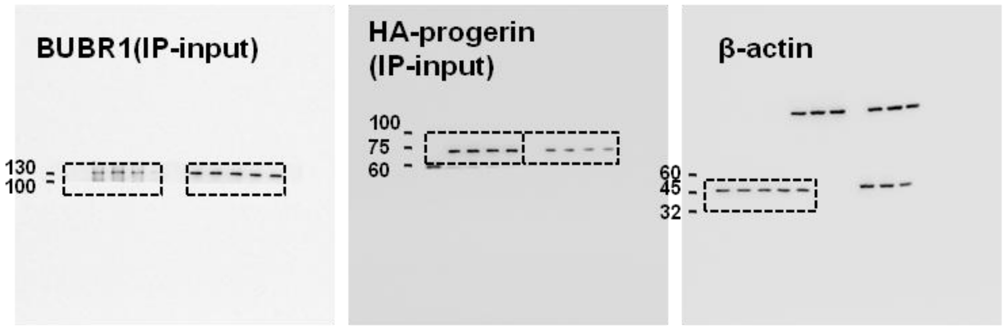

Figure 3g. Full length images of immunoblots.

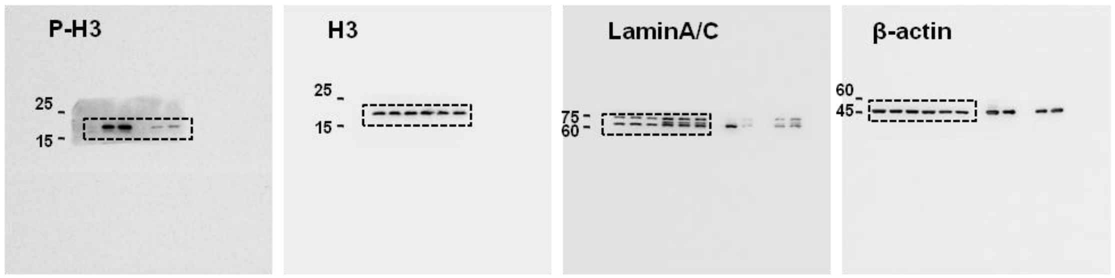

Figure 3i. Full length images of immunoblots.

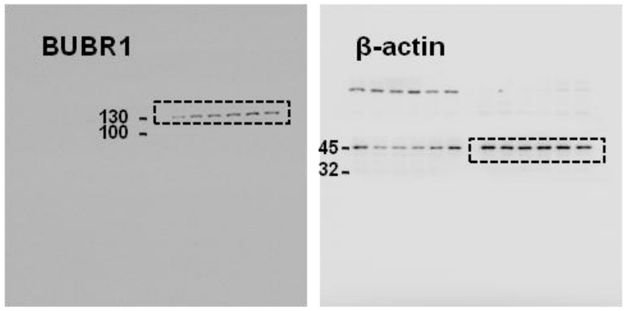

Figure 3j. Images of Immunofluorescence.

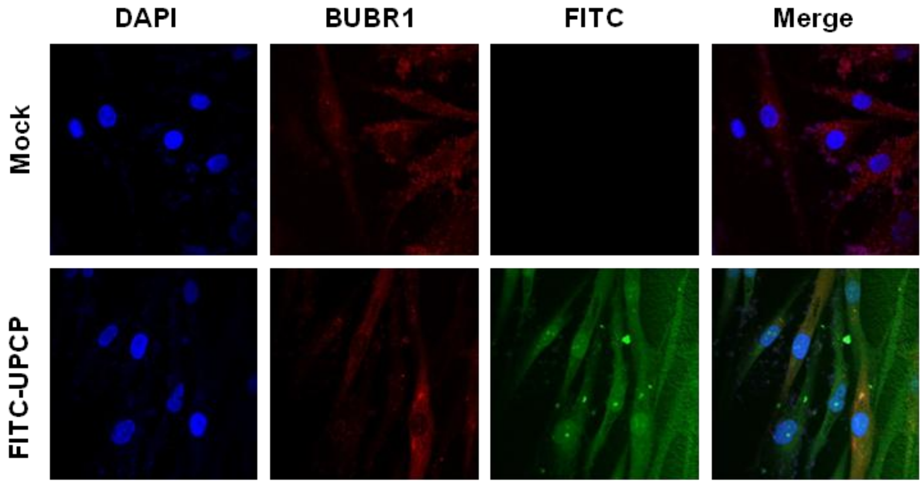

Figure 3k. Full length images of immunoblots.

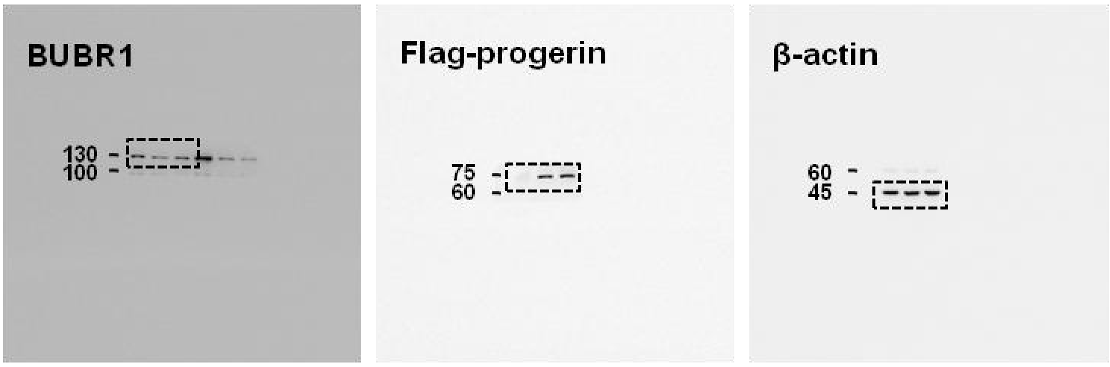

Figure 3I. Full length images of immunoblots.

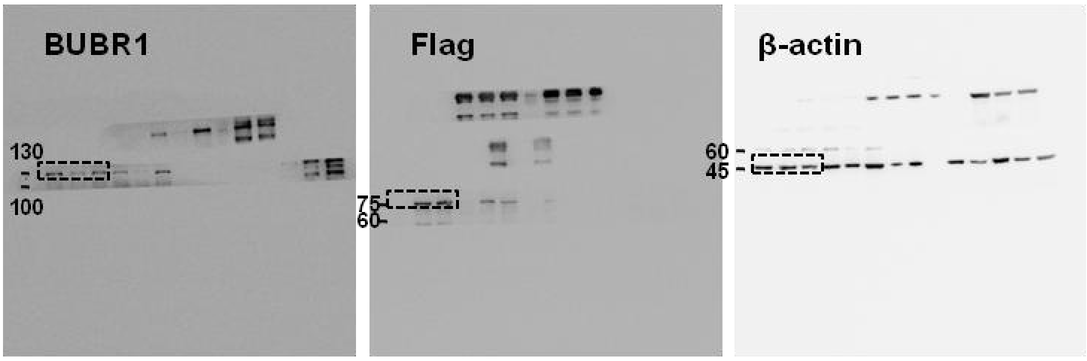

Supplement: Source Data Fig. 3 — Unprocessed western blots and/or gels. [file 43587_2023_361_MOESM21_ESM.pdf]
