# Supplementary material for: Unique progerin C-terminal peptide ameliorates Hutchinson–Gilford progeria syndrome phenotype by rescuing BUBR1
Source: Nat Aging. 2023 Feb 2;3(2):185–201. doi: 10.1038/s43587-023-00361-w (PMC10154249; doi:10.1038/s43587-023-00361-w)

Figure 4f. Full length images of immunoblots.

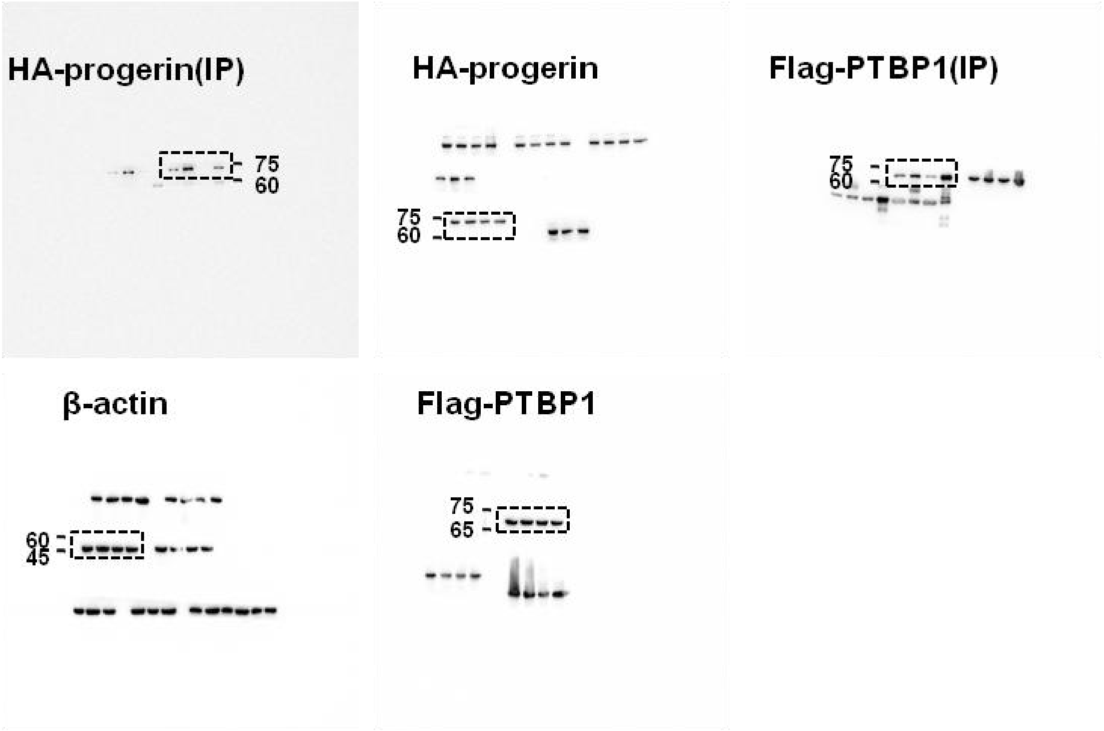

Figure 4i. Full length images of immunoblots.

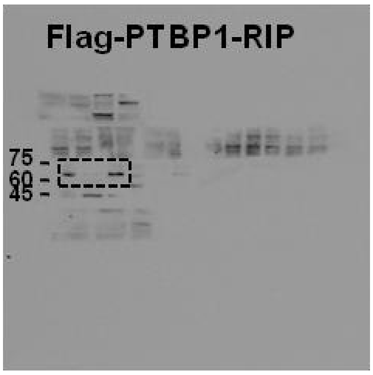

Figure 4j. Full length images of immunoblots.

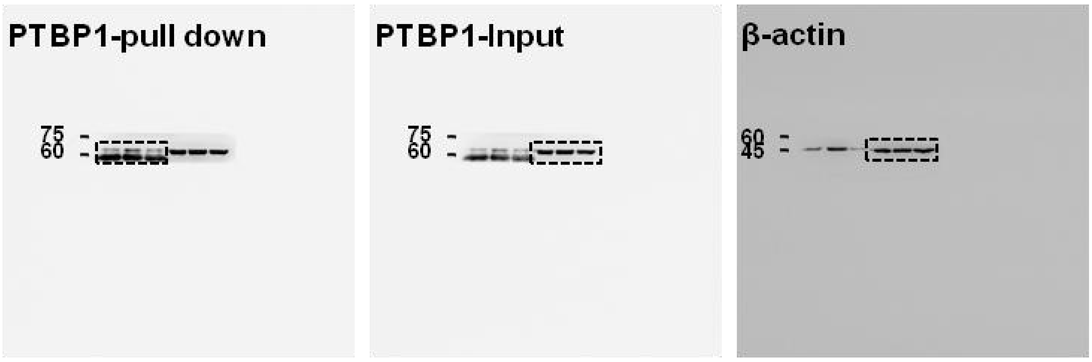

Figure 4m. Full length images of immunoblots.

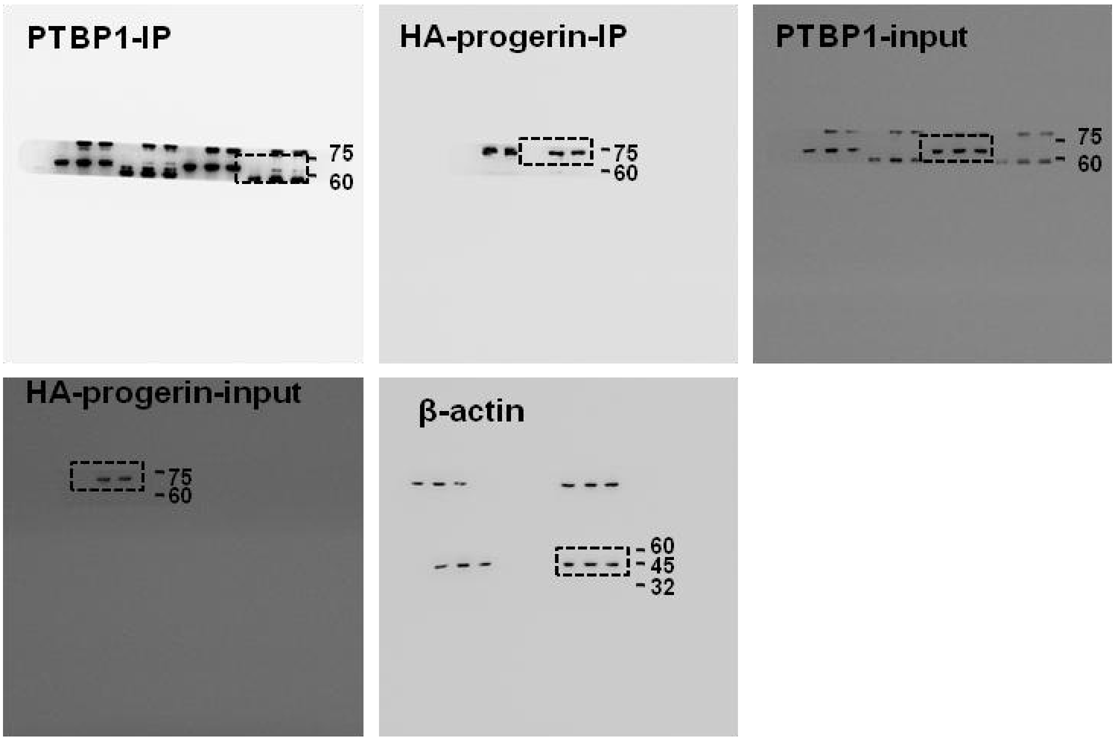

Figure 4n. Full length images of immunoblots.

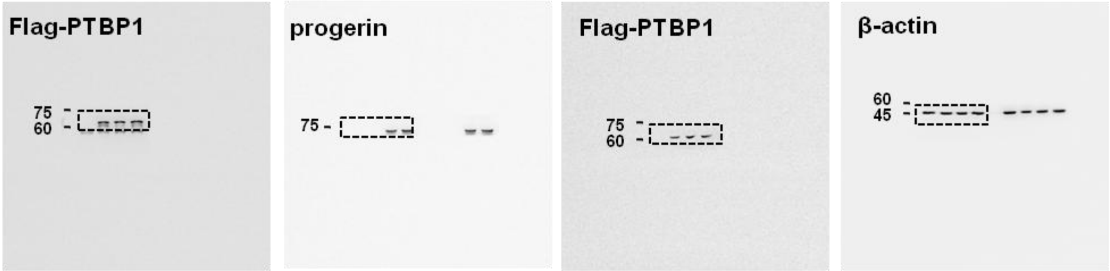

Figure 4o. Full length images of immunoblots.

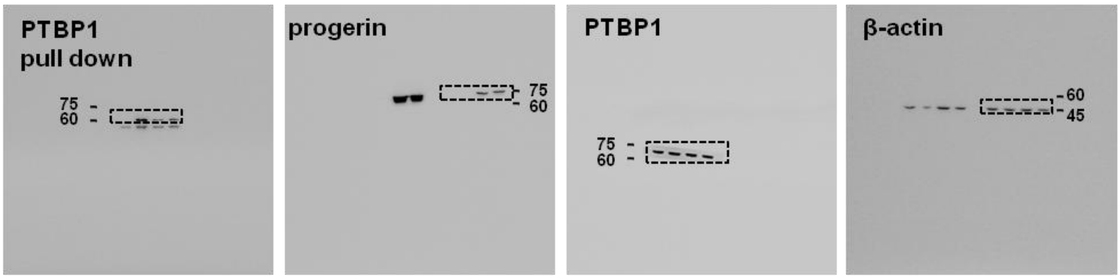

Supplement: Source Data Fig. 4 — Unprocessed western blots and/or gels. [file 43587_2023_361_MOESM22_ESM.pdf]
