# Supplementary material for: Unique progerin C-terminal peptide ameliorates Hutchinson–Gilford progeria syndrome phenotype by rescuing BUBR1
Source: Nat Aging. 2023 Feb 2;3(2):185–201. doi: 10.1038/s43587-023-00361-w (PMC10154249; doi:10.1038/s43587-023-00361-w)

Figure 5c. Full length images of immunoblots.

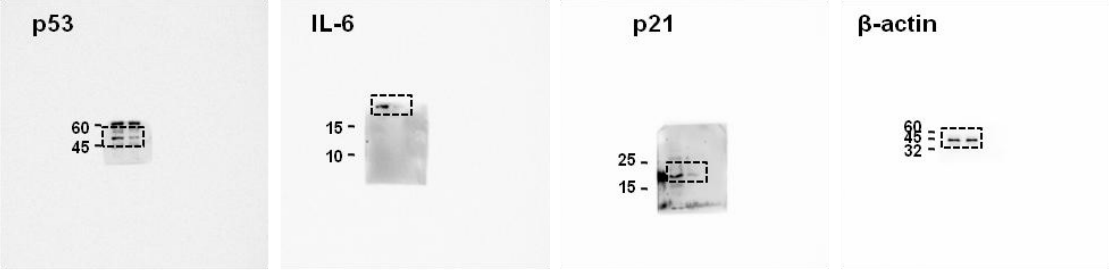

Figure 5d. Full length images of immunoblots.

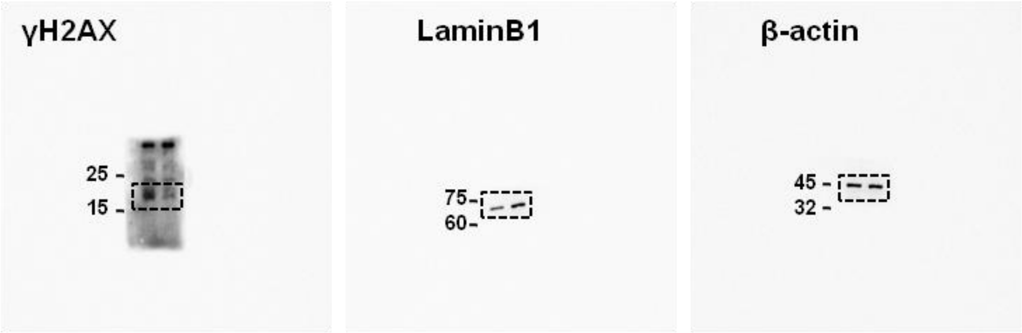

Figure 5e. Full length images of immunoblots.

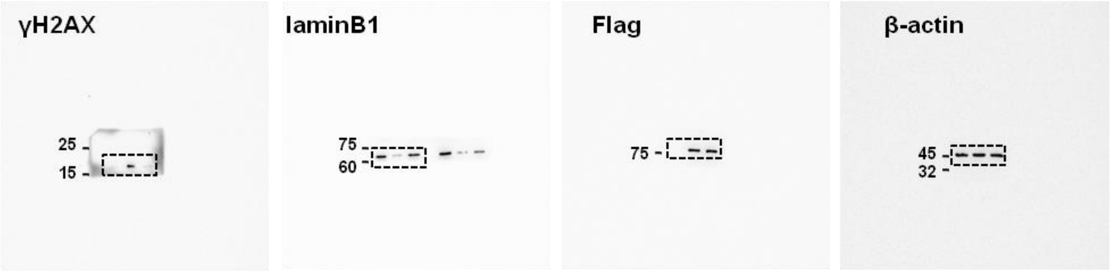

Figure 5f. Full length images of immunoblots.

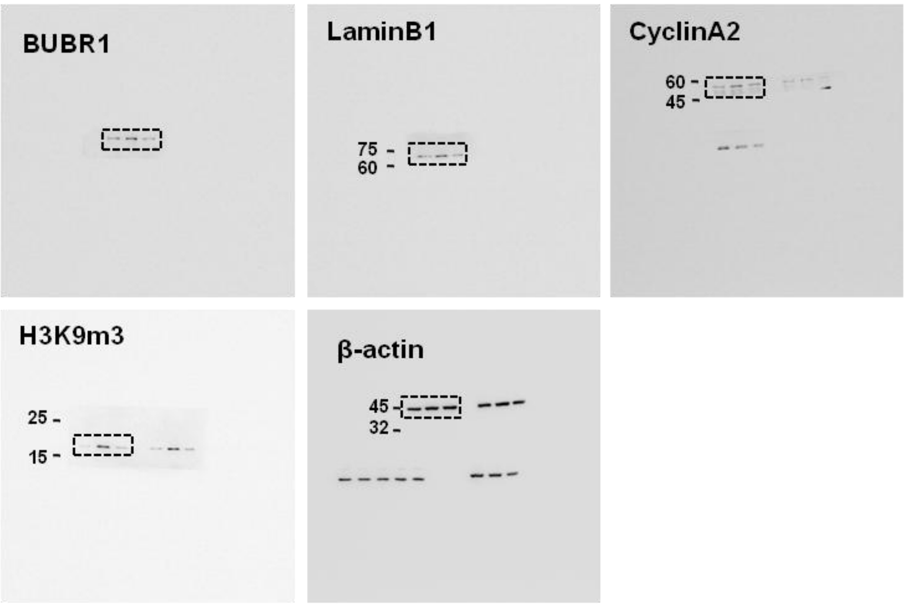

Figure 5h. Images of Immunofluorescence.

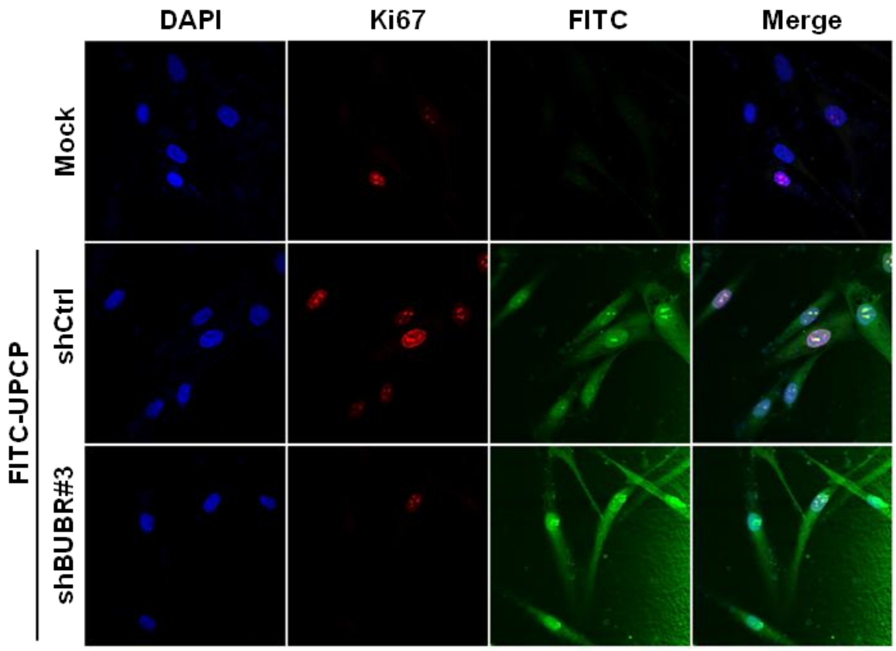

Figure 5k. Images of Immunofluorescence.

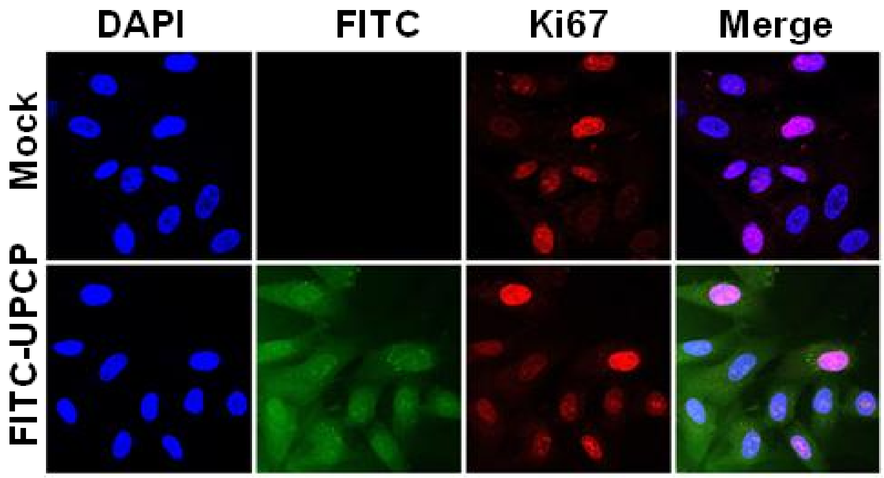

Figure 5m. Full length images of immunoblots.

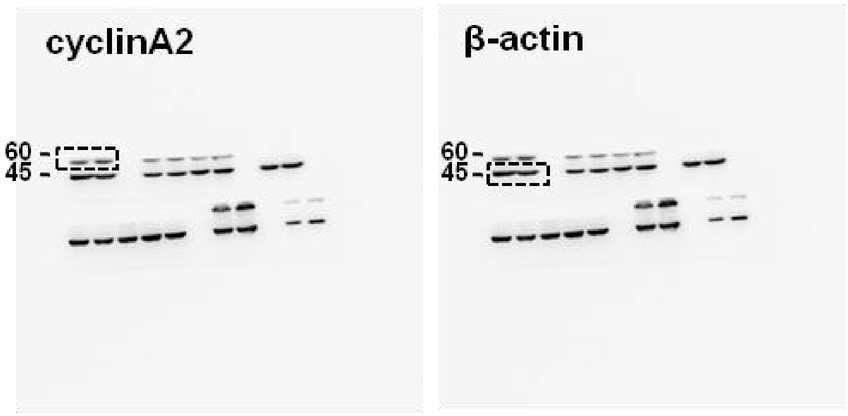

Supplement: Source Data Fig. 5 — Unprocessed western blots and/or gels. [file 43587_2023_361_MOESM23_ESM.pdf]
