# Supplementary material for: Unique progerin C-terminal peptide ameliorates Hutchinson–Gilford progeria syndrome phenotype by rescuing BUBR1
Source: Nat Aging. 2023 Feb 2;3(2):185–201. doi: 10.1038/s43587-023-00361-w (PMC10154249; doi:10.1038/s43587-023-00361-w)

Figure 6a,d. Representative photographs of treatment with UPCP for 12 week *Lmna*$^{G609G/G609G}$ female and male mice

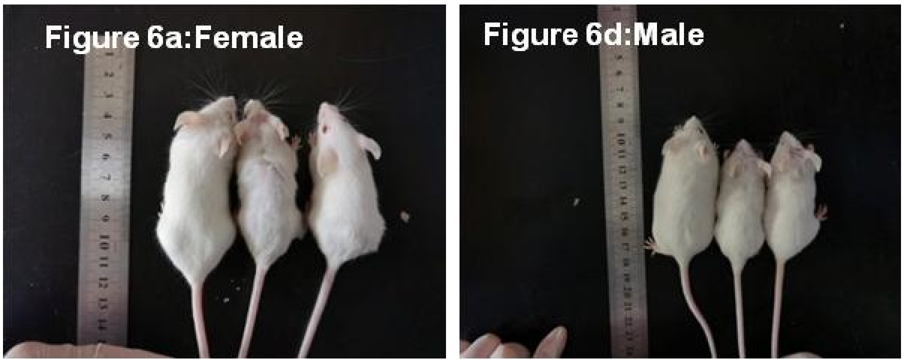

Figure 6g. Visual image of female mice motion trajectory in open field experiment.

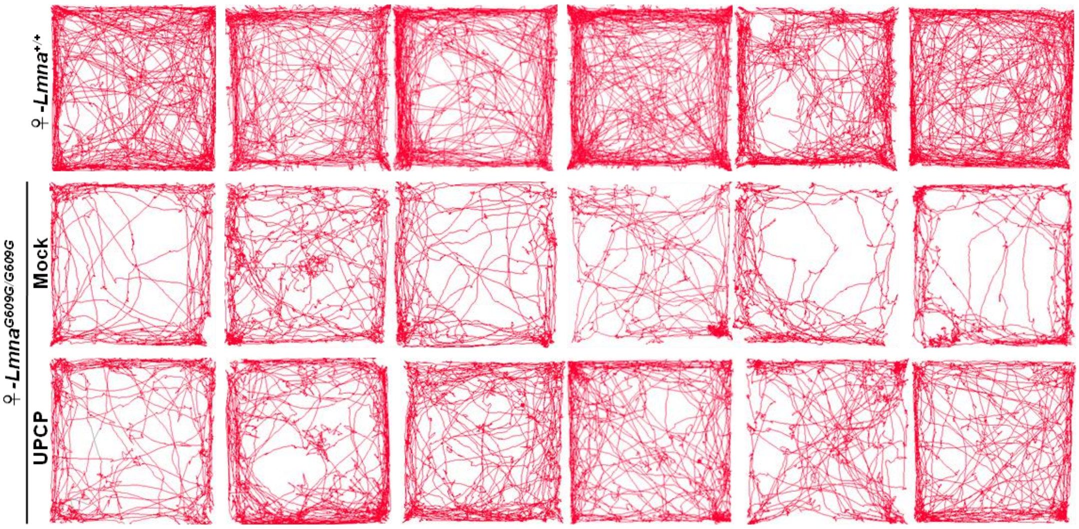

Figure 6h. Visual image of male mice motion trajectory in open field experiment.

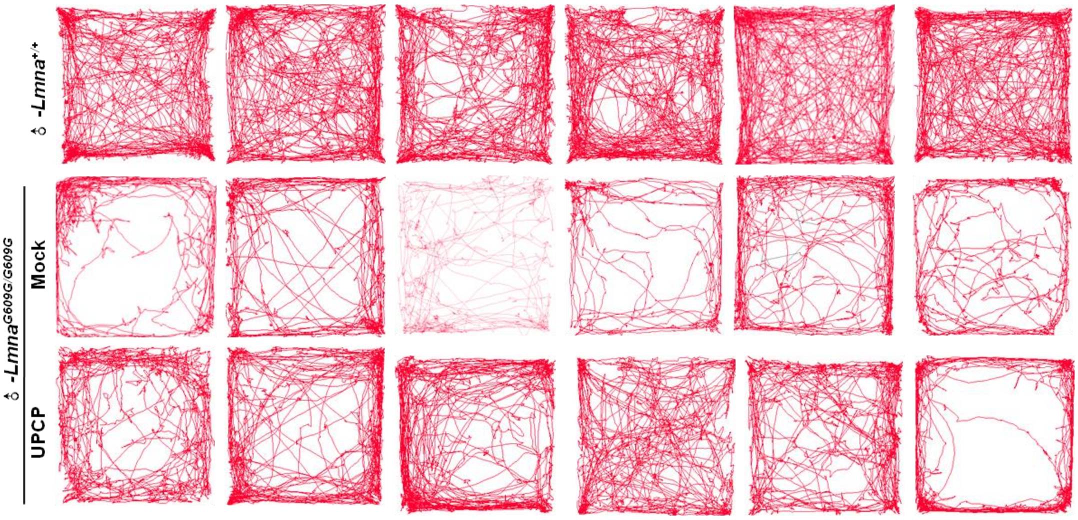

Supplement: Source Data Fig. 6 — Unprocessed western blots and/or gels. [file 43587_2023_361_MOESM24_ESM.pdf]
