# Supplementary material for: Unique progerin C-terminal peptide ameliorates Hutchinson–Gilford progeria syndrome phenotype by rescuing BUBR1
Source: Nat Aging. 2023 Feb 2;3(2):185–201. doi: 10.1038/s43587-023-00361-w (PMC10154249; doi:10.1038/s43587-023-00361-w)

Figure 7a. Immunohistochemistry of BubR1 in skin, aorta, lung and spleen.

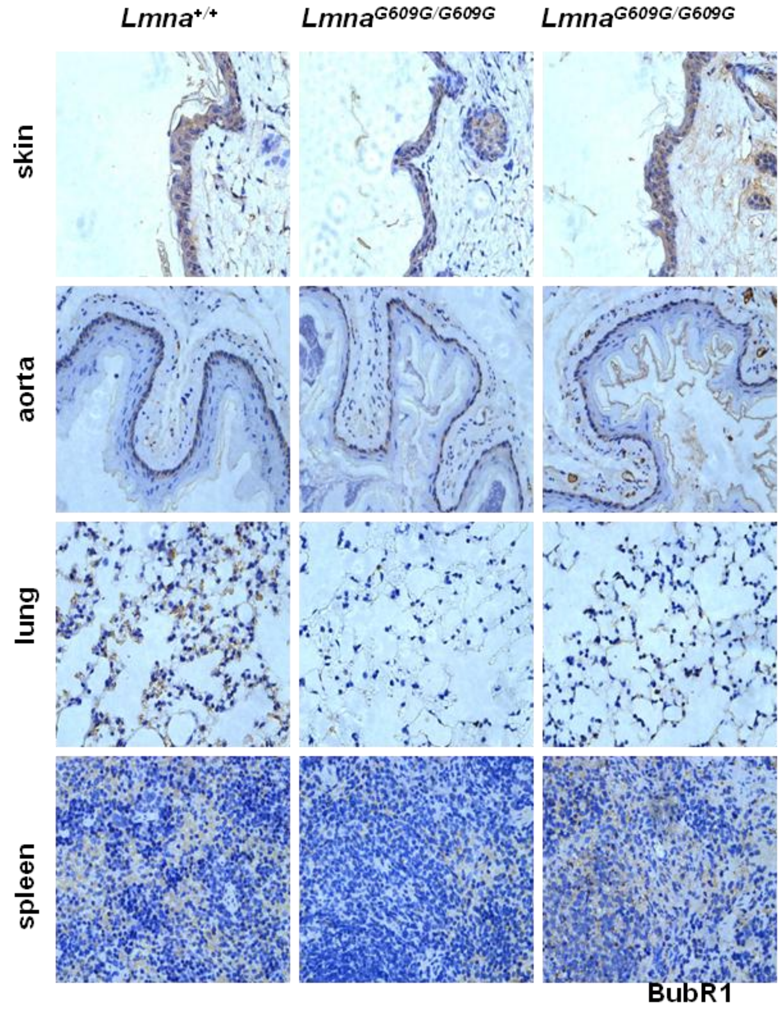

Figure 7c. Histological analyses of the skin derived from mice treatment with or without UPCP

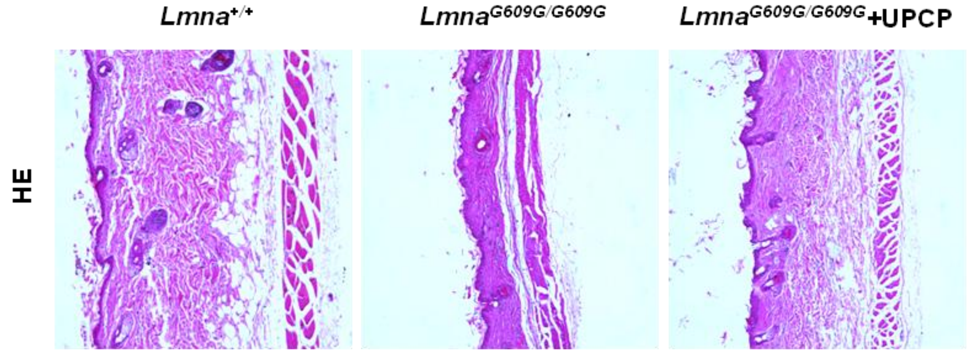

Figure 7e. Masson staining of aorta derived from mice treatment with or without UPCP.

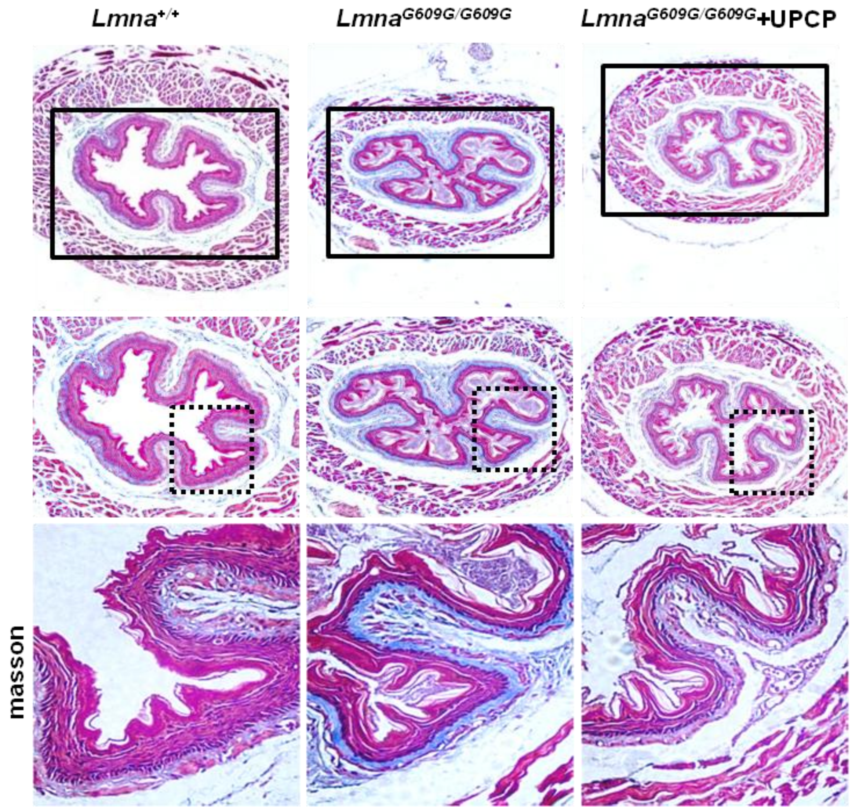

Figure 7g. Masson staining of aorta derived from mice treatment with or without UPCP.

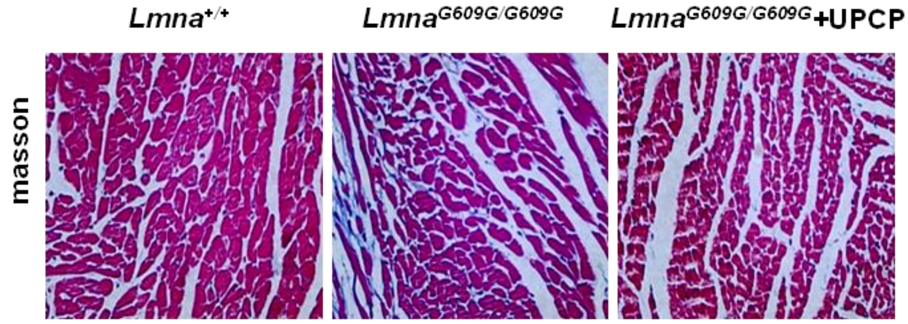

Figure 7g. Masson staining of aorta derived from mice treatment with or without UPCP.

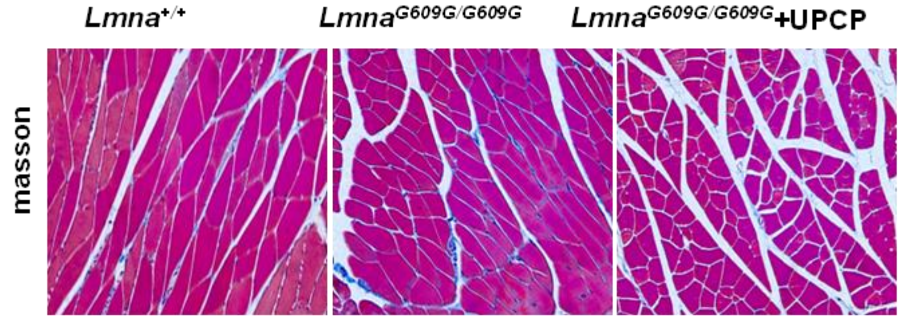

Supplement: Source Data Fig. 7 — Unprocessed western blots and/or gels. [file 43587_2023_361_MOESM25_ESM.pdf]
