# Supplementary material for: Unique progerin C-terminal peptide ameliorates Hutchinson–Gilford progeria syndrome phenotype by rescuing BUBR1
Source: Nat Aging. 2023 Feb 2;3(2):185–201. doi: 10.1038/s43587-023-00361-w (PMC10154249; doi:10.1038/s43587-023-00361-w)

Extended Data Figure 1a. Full length images of immunoblots.

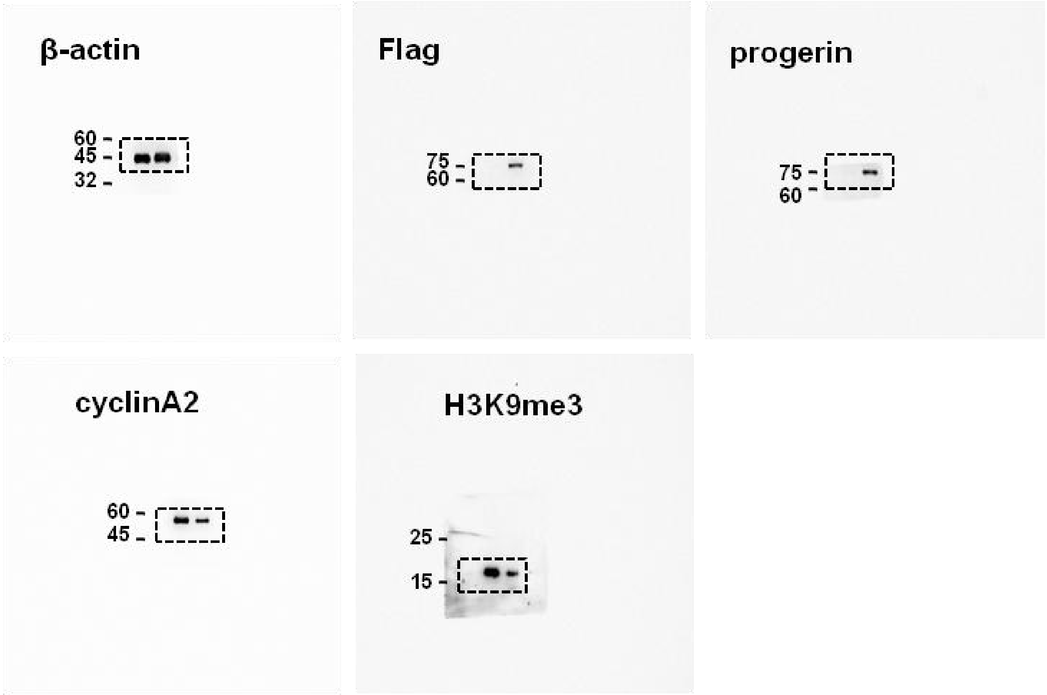

Extended Data Figure 1b. Images of β-Gal staining

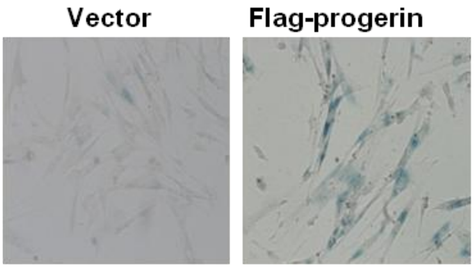

Extended Data Figure 1c. Images of Immunofluorescence.

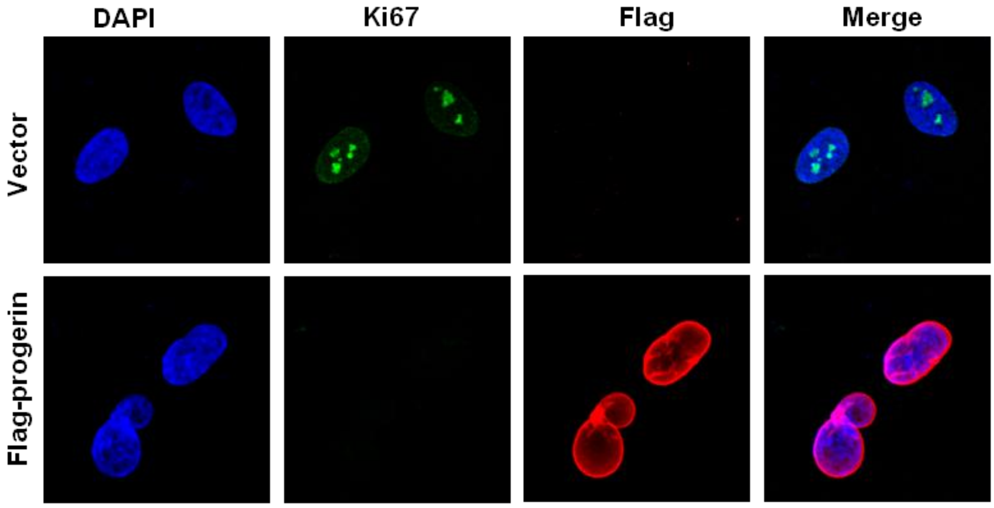

Extended Data Figure 1e. Images of Immunofluorescence.

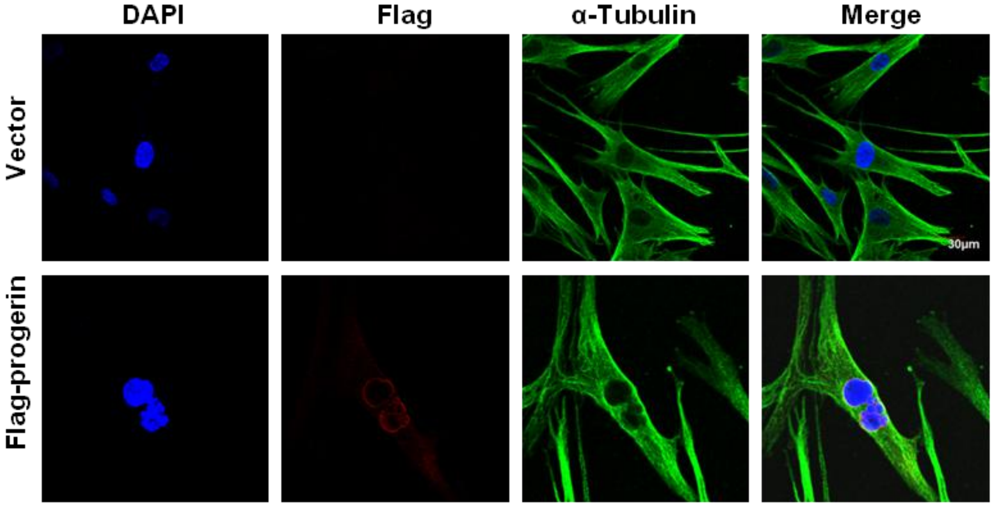

Extended Data Figure 1g. Images of Immunofluorescence.

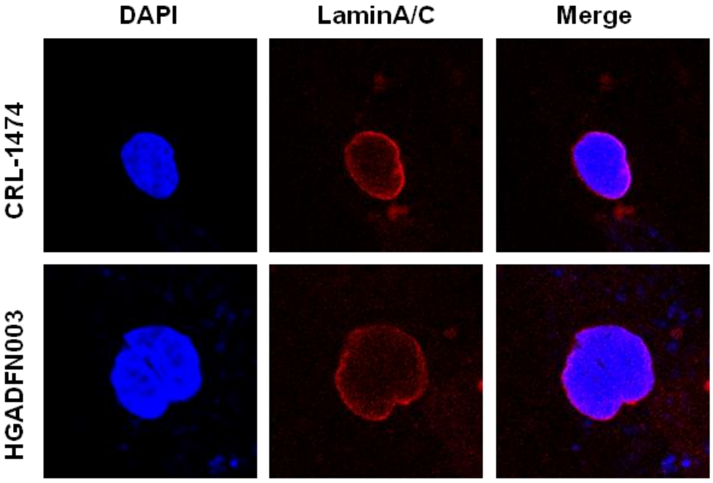

Extended Data Figure 1i. Chromosomes spread and visualize.

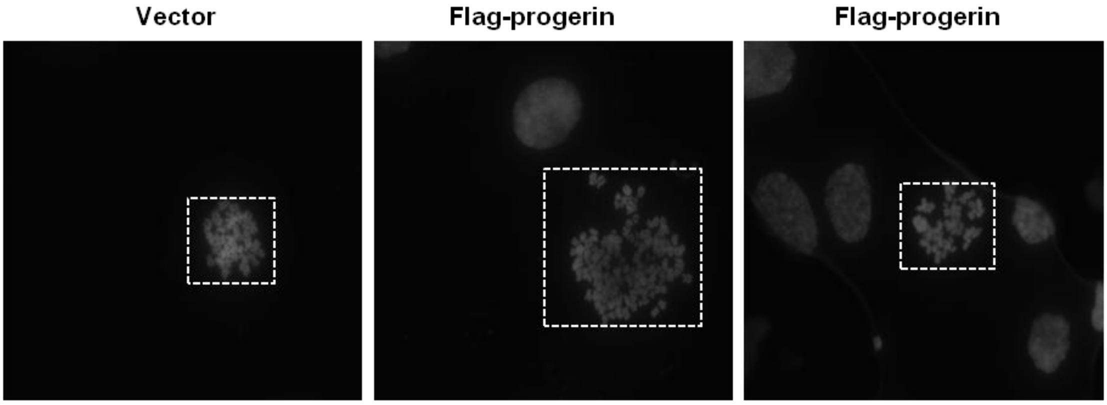

Extended Data Figure 1k. Chromosomes spread and visualize.

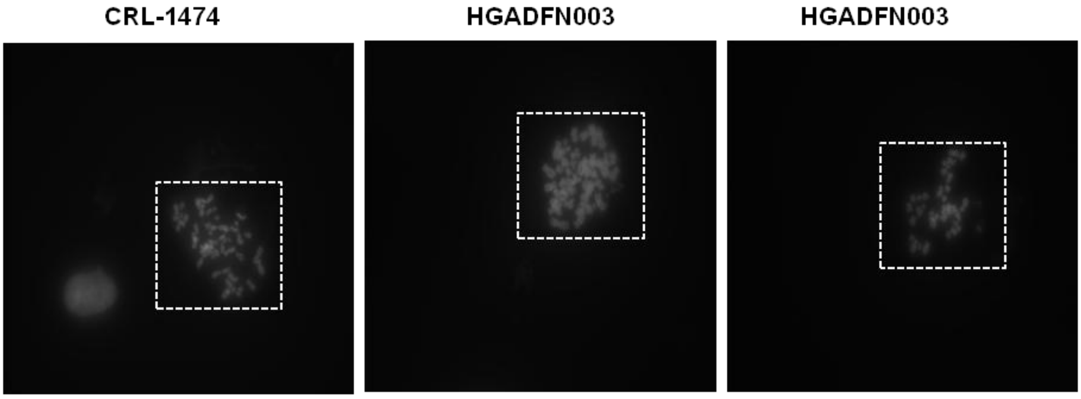

Extended Data Figure 1m. Images of Immunofluorescence.

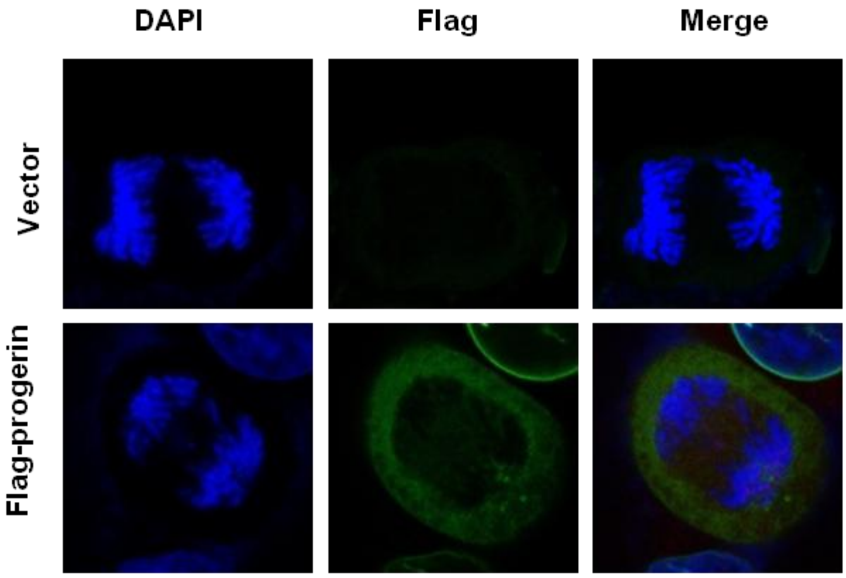

Extended Data Figure 1o. Images of Immunofluorescence.

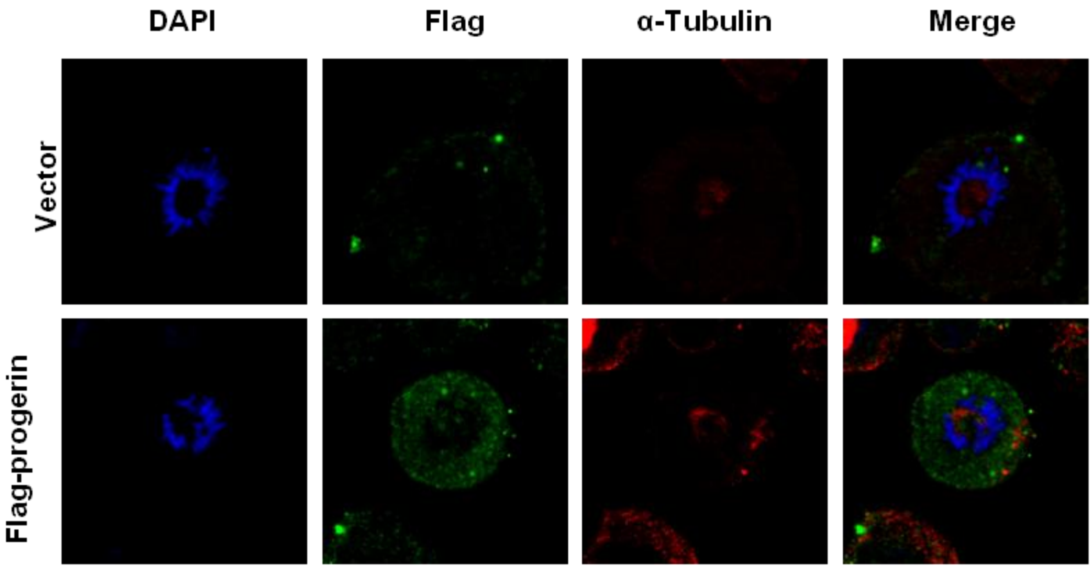

Supplement: Source Data Extended Data Fig. 1 — Unprocessed western blots and/or gels. [file 43587_2023_361_MOESM26_ESM.pdf]
