# Supplementary material for: Unique progerin C-terminal peptide ameliorates Hutchinson–Gilford progeria syndrome phenotype by rescuing BUBR1
Source: Nat Aging. 2023 Feb 2;3(2):185–201. doi: 10.1038/s43587-023-00361-w (PMC10154249; doi:10.1038/s43587-023-00361-w)

Extended Data Figure 2a. Full length images of immunoblots.

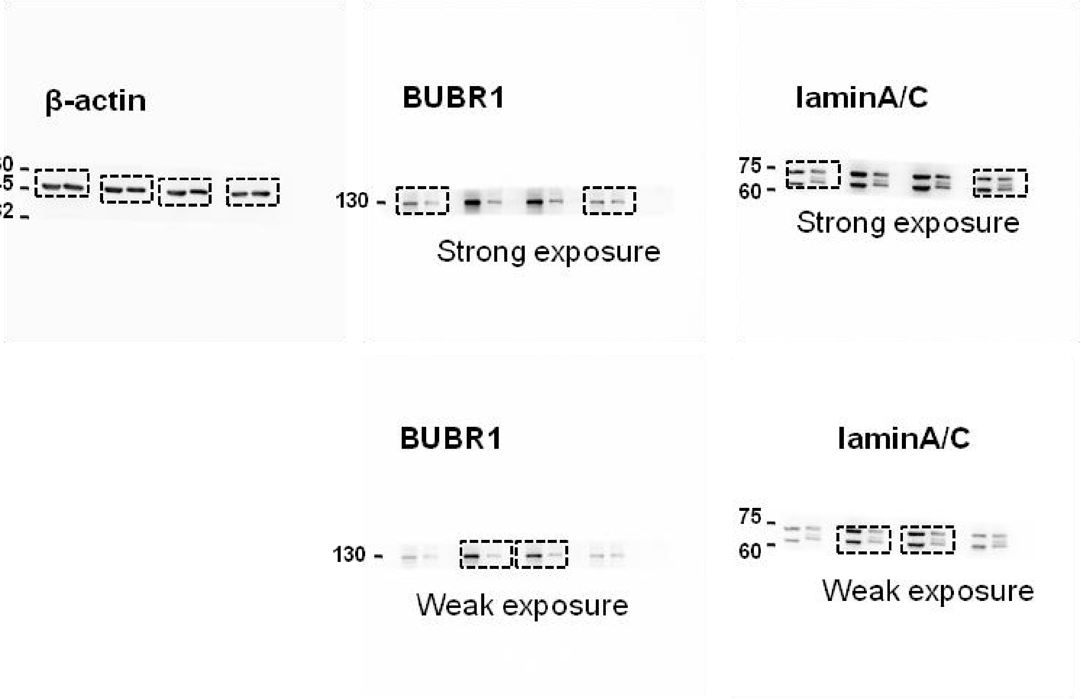

Extended Data Figure 2c. Full length images of immunoblots.

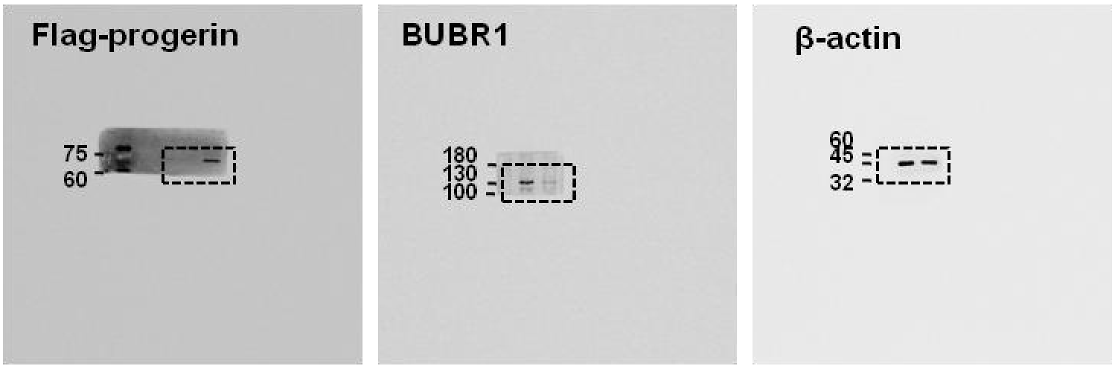

Extended Data Figure 2d. Full length images of immunoblots.

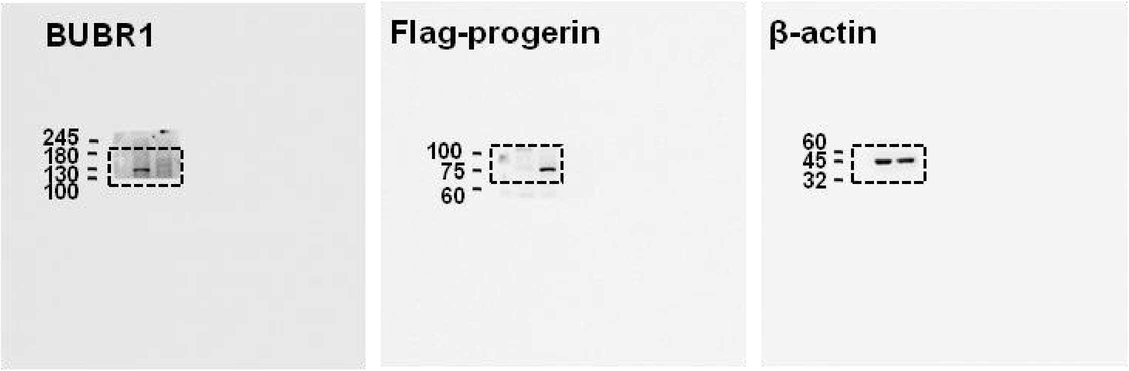

Extended Data Figure 2e. Full length images of immunoblots.

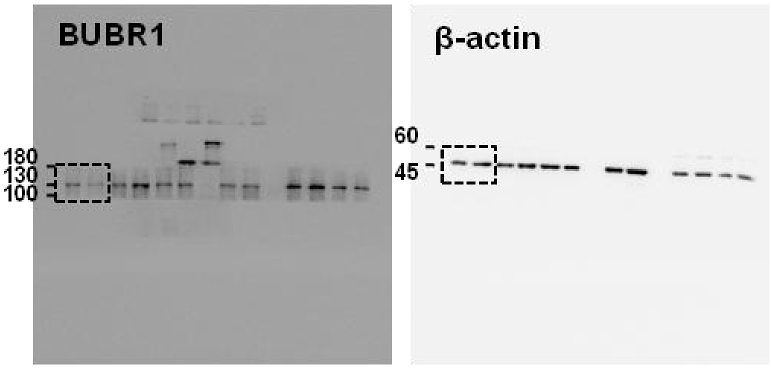

Extended Data Figure 2f. Images of β-Gal staining

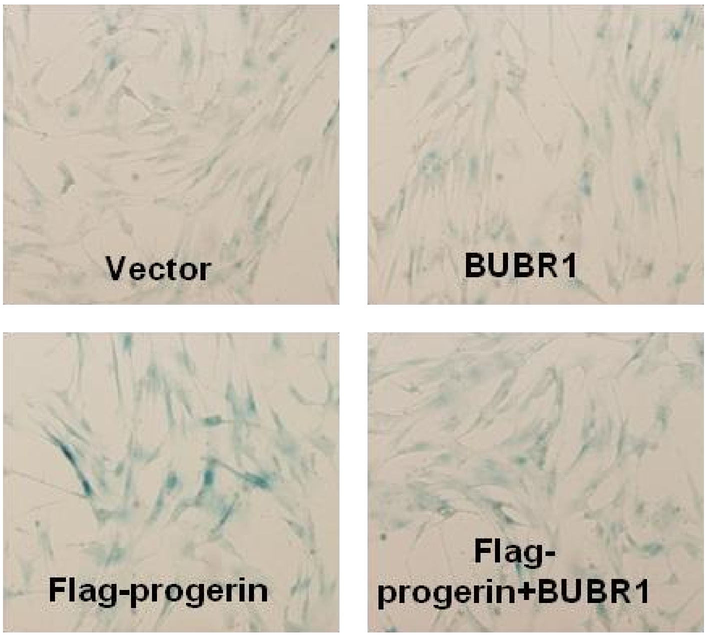

Extended Data Figure 2g. Images of β-Gal staining

HGADFN003

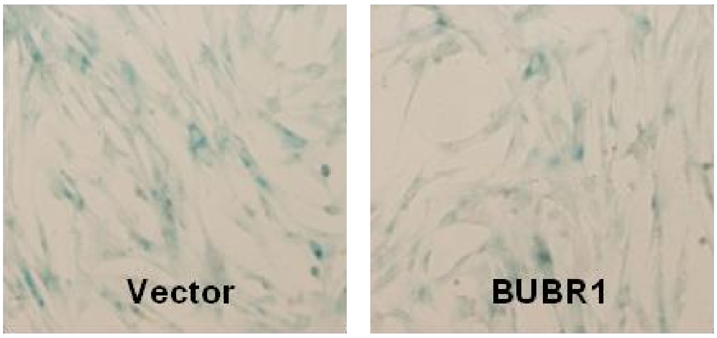

Extended Data Figure 2h. Full length images of immunoblots.

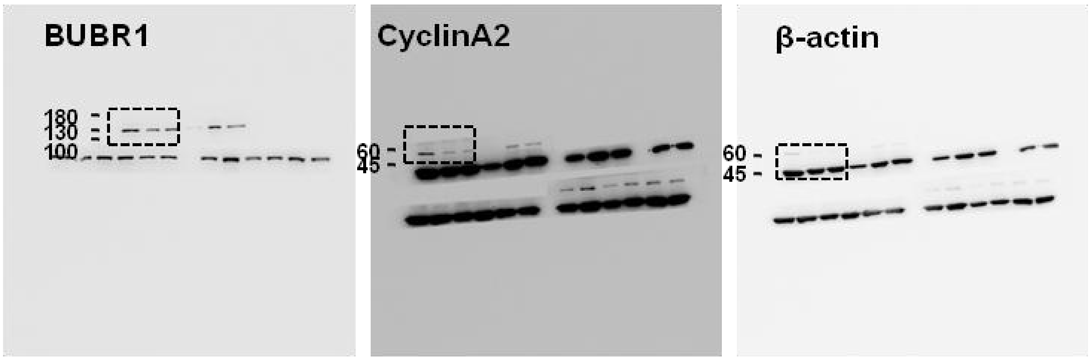

Extended Data Figure 2j. Images of Immunofluorescence.

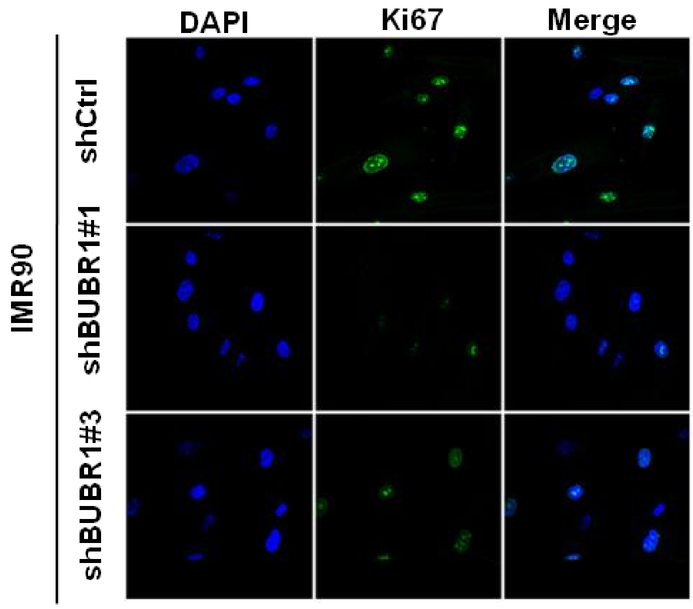

Extended Data Figure 2l. Images of β-Gal staining

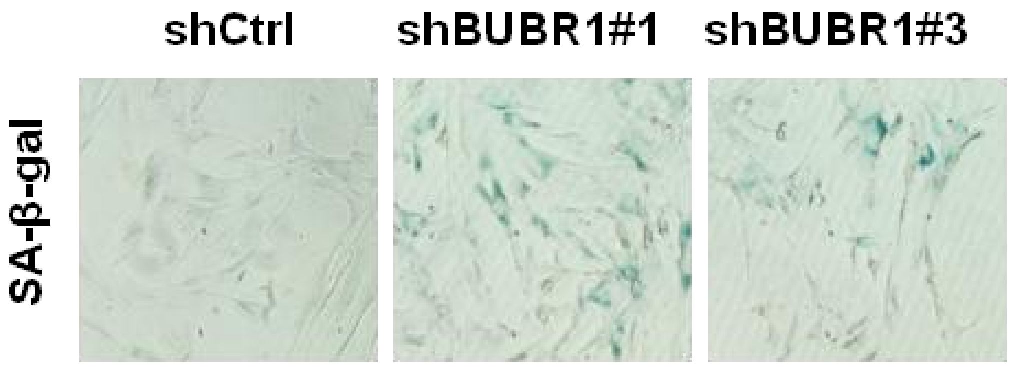

Supplement: Source Data Extended Data Fig. 2 — Unprocessed western blots and/or gels. [file 43587_2023_361_MOESM27_ESM.pdf]
