# Supplementary material for: Unique progerin C-terminal peptide ameliorates Hutchinson–Gilford progeria syndrome phenotype by rescuing BUBR1
Source: Nat Aging. 2023 Feb 2;3(2):185–201. doi: 10.1038/s43587-023-00361-w (PMC10154249; doi:10.1038/s43587-023-00361-w)
Supplement: Source Data Extended Data Fig. 3 — Unprocessed western blots and/or gels. [file 43587_2023_361_MOESM28_ESM.pdf]

Extended Data Figure 3a. Full length images of immunoblots.

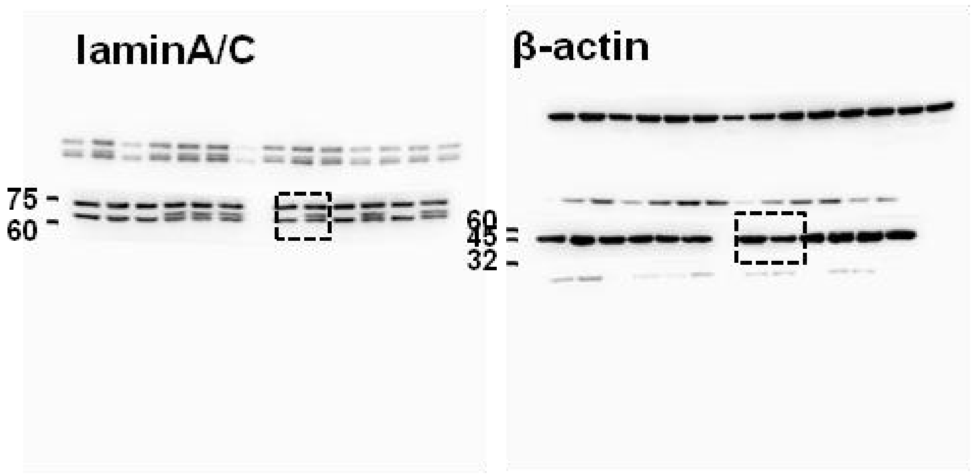

Extended Data Figure 3b. Images of Immunofluorescence.

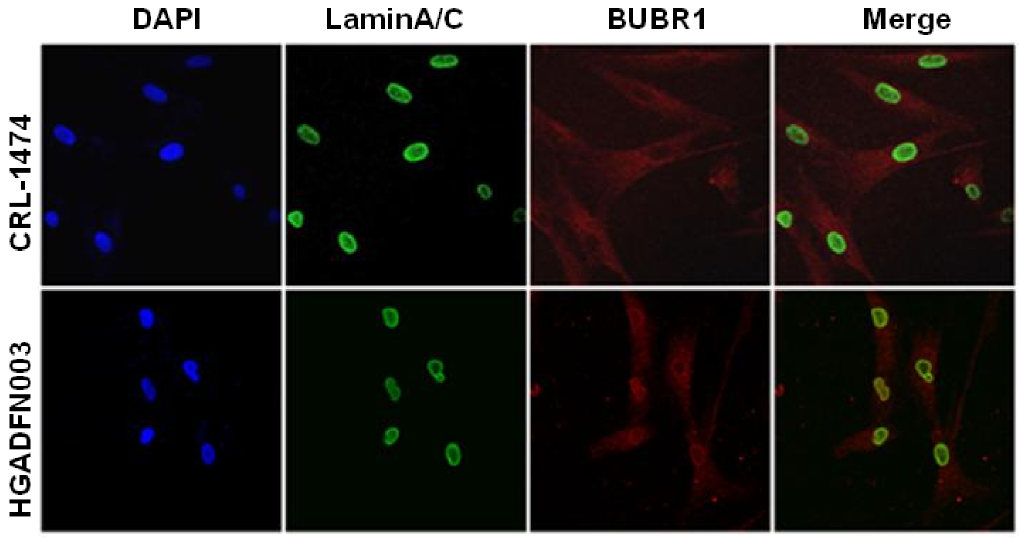

Extended Data Figure 3d. Full length images of immunoblots.

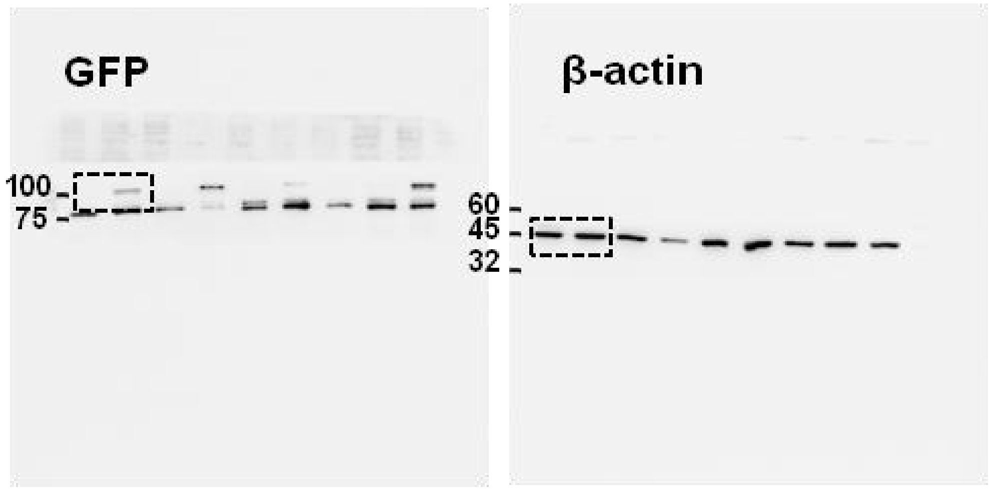

Extended Data Figure 3e. Images of Immunofluorescence.

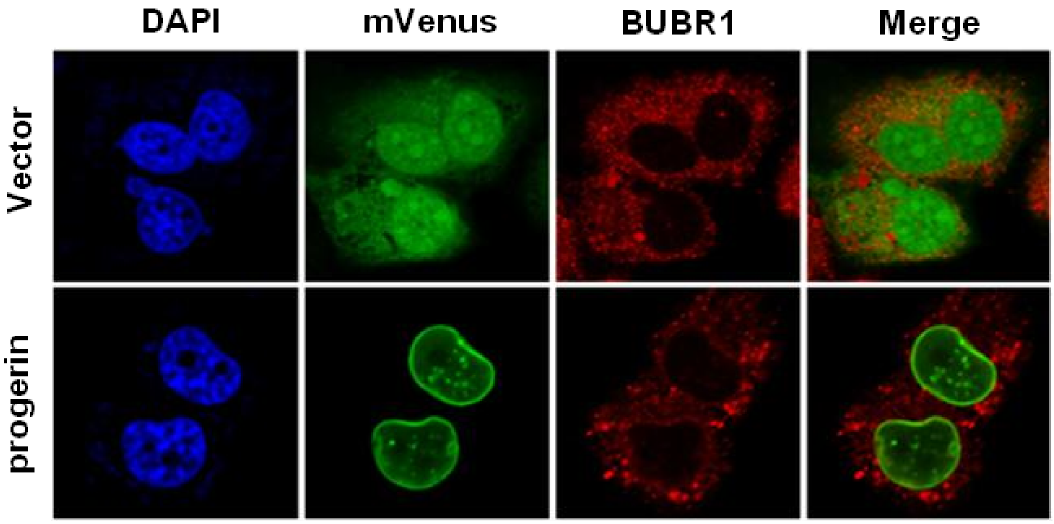

Extended Data Figure 3g. Full length images of immunoblots.

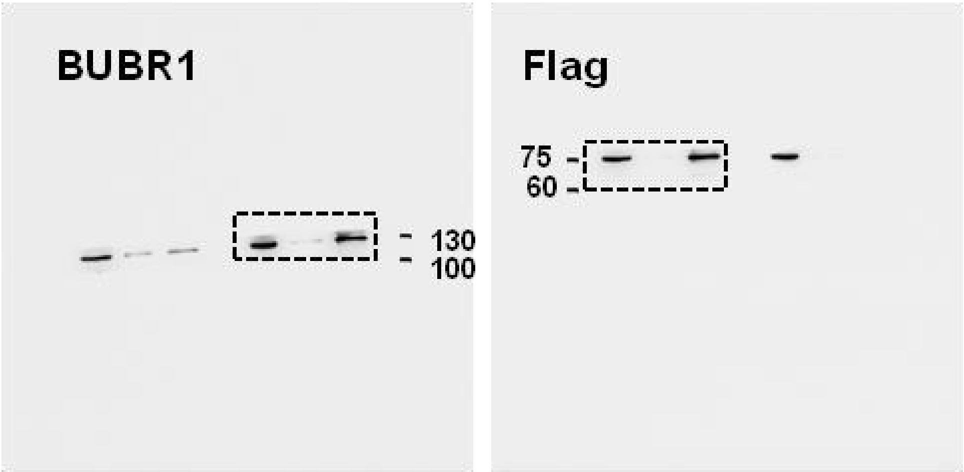

Extended Data Figure 3h. Full length images of immunoblots.

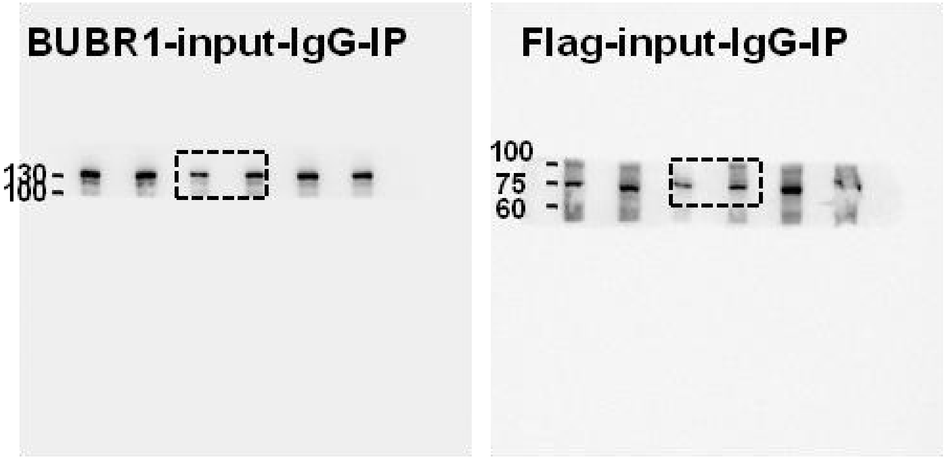

Extended Data Figure 3i. Full length images of immunoblots.

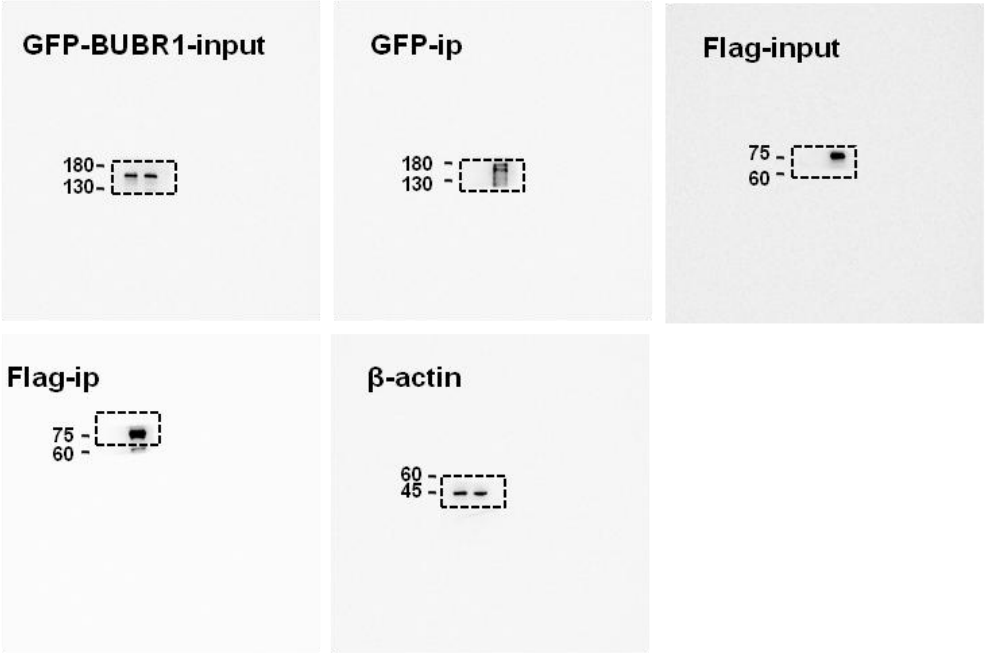

Extended Data Figure 3j. Full length images of immunoblots.

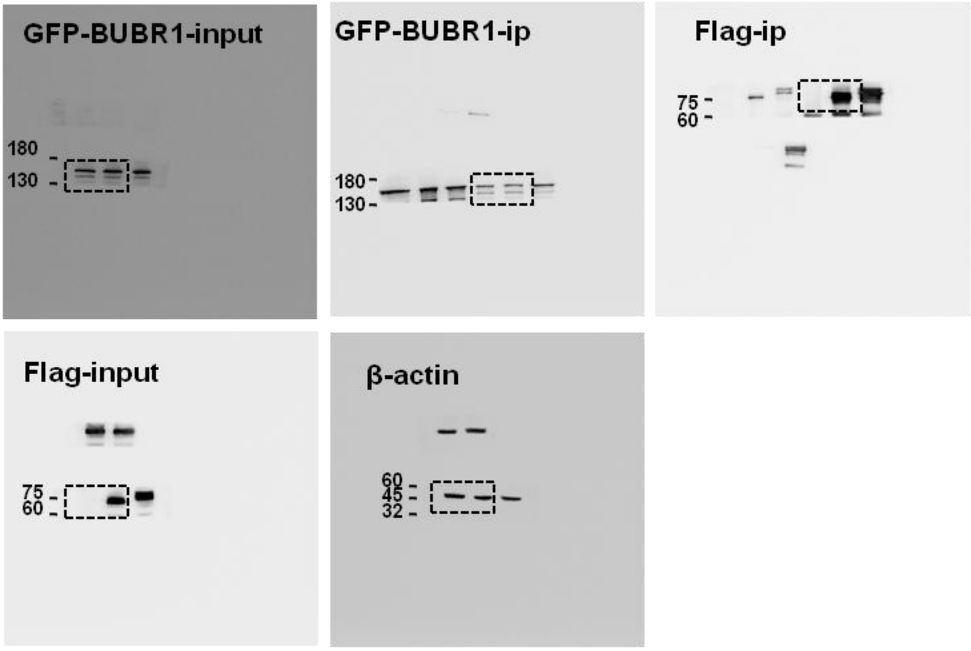

Extended Data Figure 3k. Full length images of immunoblots.

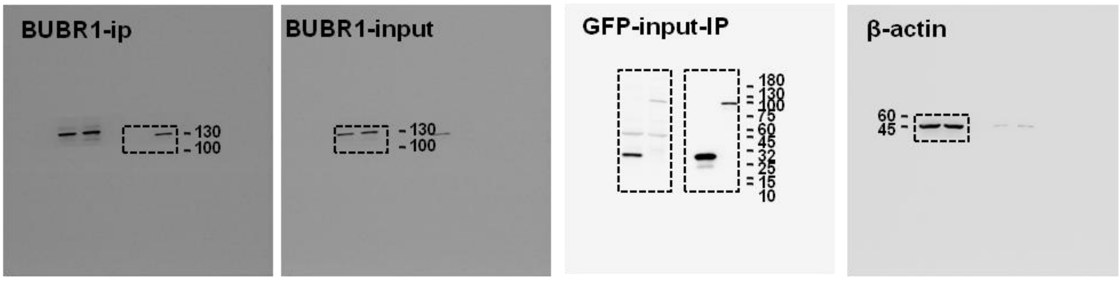

Extended Data Figure 3l. Full length images of immunoblots.

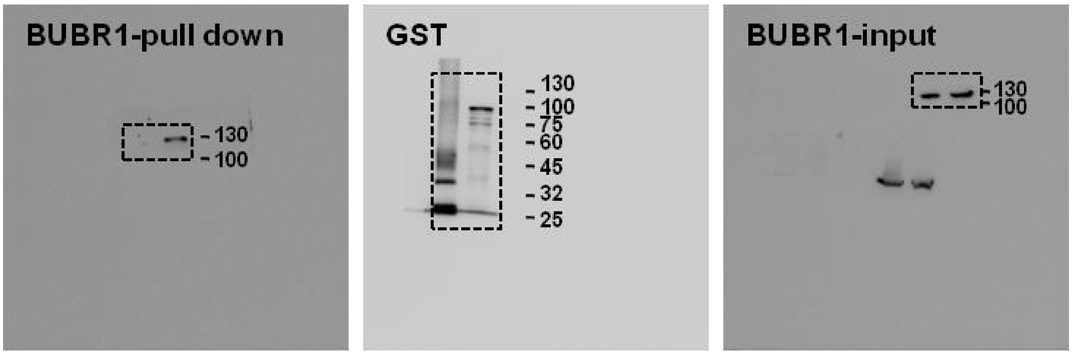
BUBR1-pull down
130
100
GST
130
100
75
60
45
32
25
BUBR1-input
130
100
