# Supplementary material for: Unique progerin C-terminal peptide ameliorates Hutchinson–Gilford progeria syndrome phenotype by rescuing BUBR1
Source: Nat Aging. 2023 Feb 2;3(2):185–201. doi: 10.1038/s43587-023-00361-w (PMC10154249; doi:10.1038/s43587-023-00361-w)

Extended Data Figure 4a. Images of Immunofluorescence.

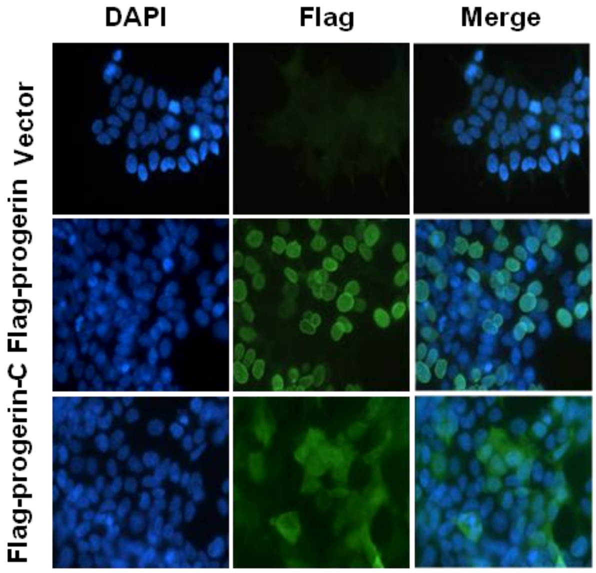

Extended Data Figure 4c. Full length images of immunoblots.

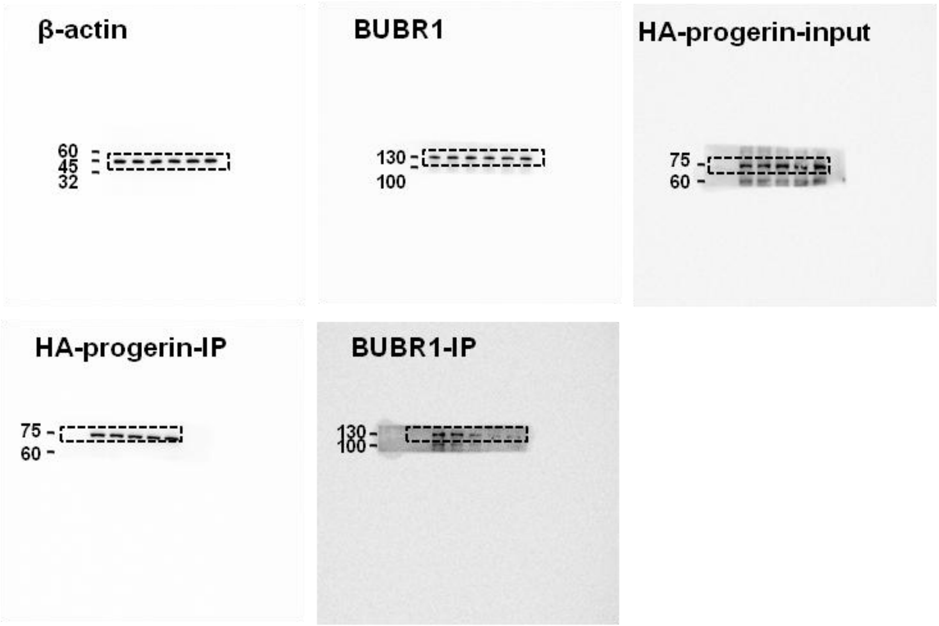

Extended Data Figure 4d. Full length images of immunoblots.

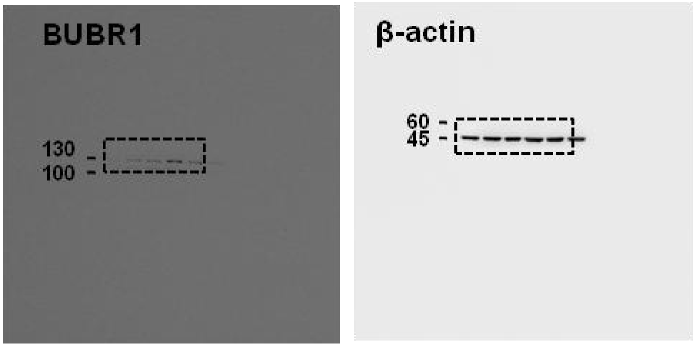

Extended Data Figure 4e. Full length images of immunoblots.

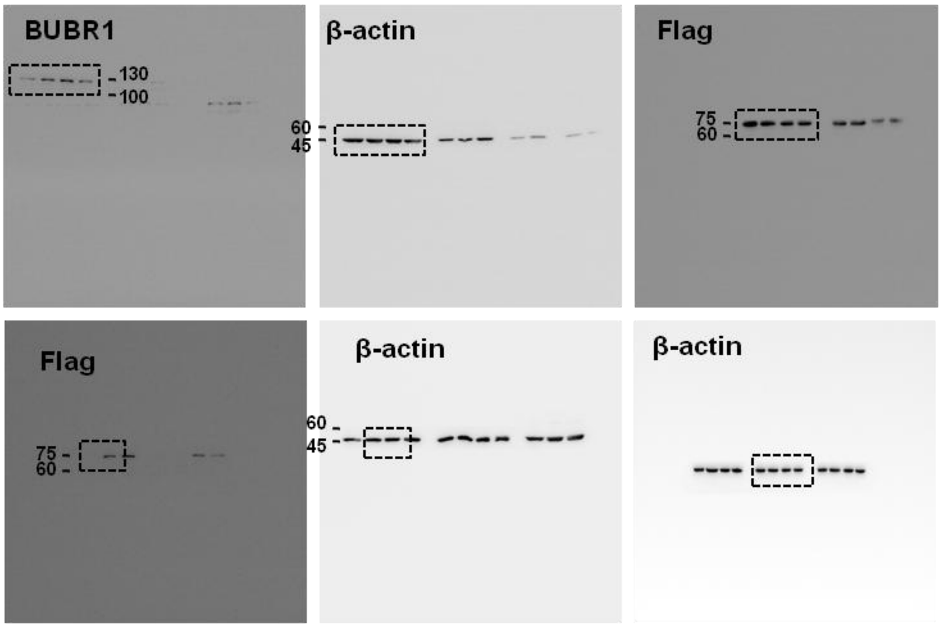

Extended Data Figure 4f. Full length images of immunoblots.

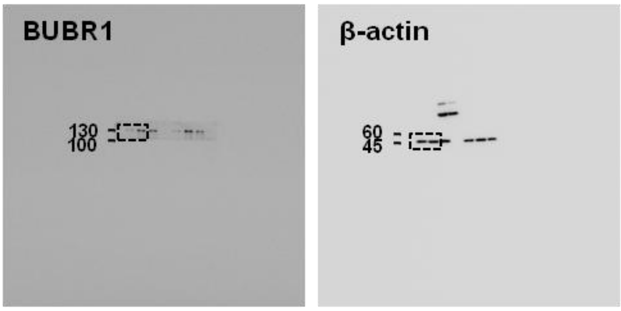

Supplement: Source Data Extended Data Fig. 4 — Unprocessed western blots and/or gels. [file 43587_2023_361_MOESM29_ESM.pdf]
