# Supplementary material for: Unique progerin C-terminal peptide ameliorates Hutchinson–Gilford progeria syndrome phenotype by rescuing BUBR1
Source: Nat Aging. 2023 Feb 2;3(2):185–201. doi: 10.1038/s43587-023-00361-w (PMC10154249; doi:10.1038/s43587-023-00361-w)

Extended Data Figure 5a. Full length images of immunoblots.

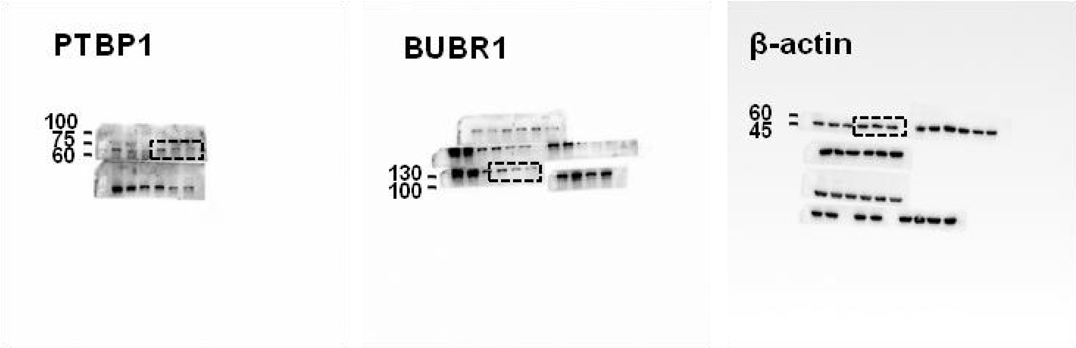

Extended Data Figure 5b. Full length images of immunoblots.

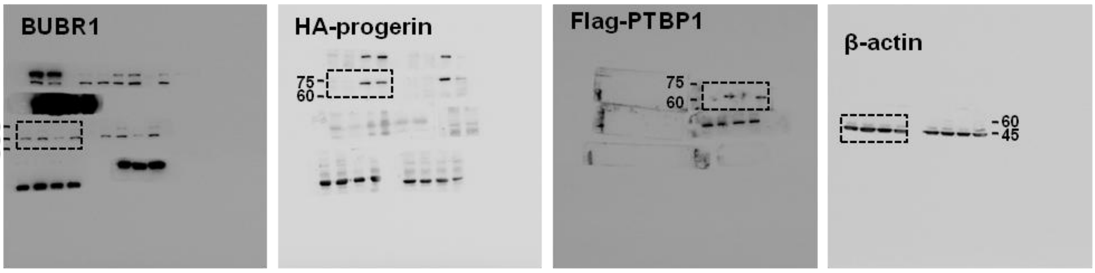

Extended Data Figure 5c. Full length images of immunoblots.

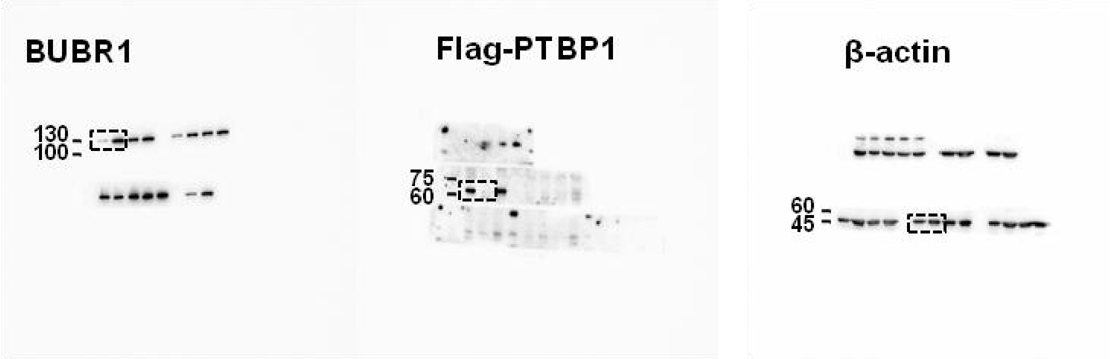

Extended Data Figure 5d. Full length images of immunoblots.

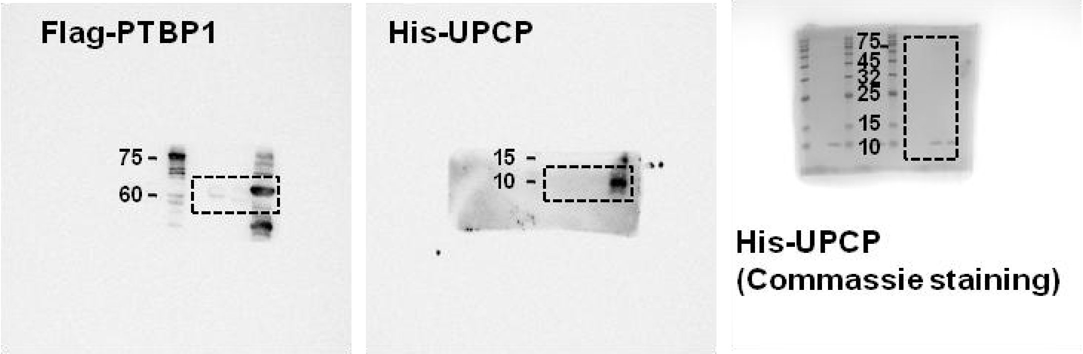

Extended Data Figure 5e. Images of Immunofluorescence.

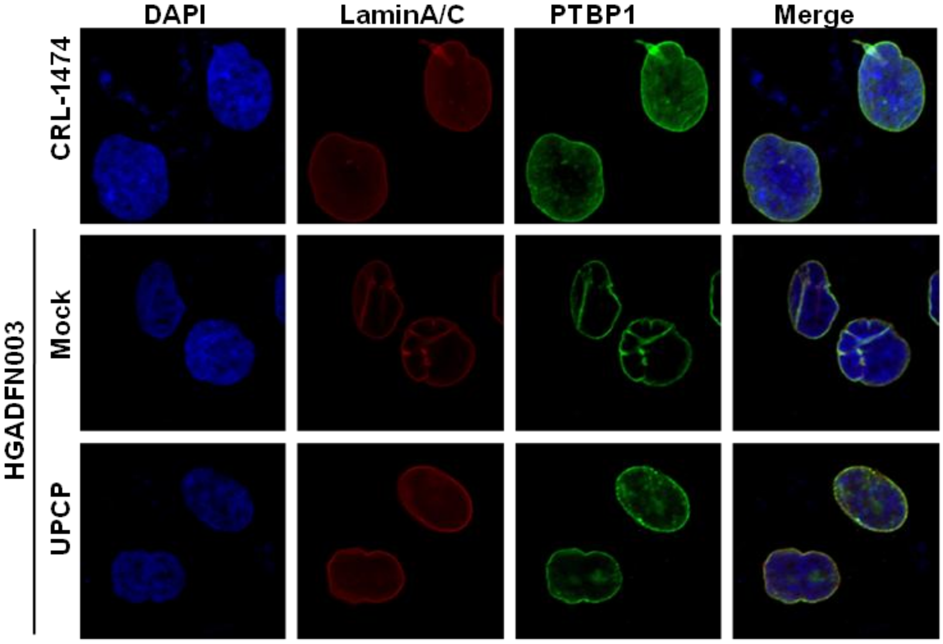

Supplement: Source Data Extended Data Fig. 5 — Unprocessed western blots and/or gels. [file 43587_2023_361_MOESM30_ESM.pdf]
