# Supplementary material for: Unique progerin C-terminal peptide ameliorates Hutchinson–Gilford progeria syndrome phenotype by rescuing BUBR1
Source: Nat Aging. 2023 Feb 2;3(2):185–201. doi: 10.1038/s43587-023-00361-w (PMC10154249; doi:10.1038/s43587-023-00361-w)

Extended Data Figure 6a. Images of β-Gal staining

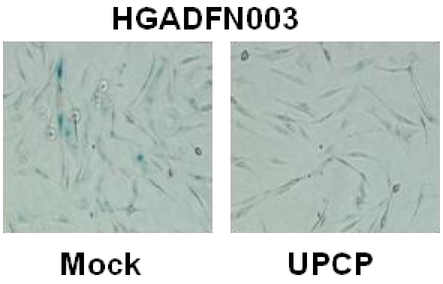

Extended Data Figure 6b. Images of Immunofluorescence.

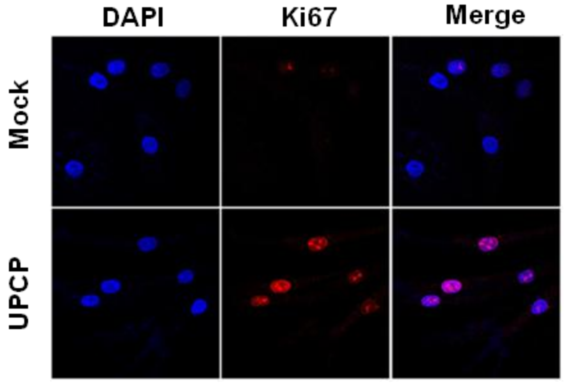

Extended Data Figure 6c. Images of β-Gal staining

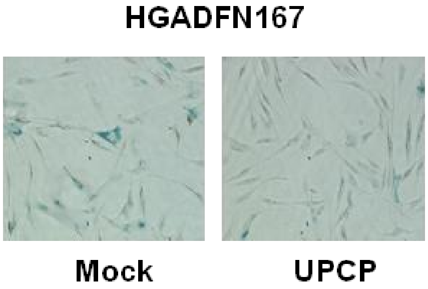

Extended Data Figure 6e. Images of Immunofluorescence.

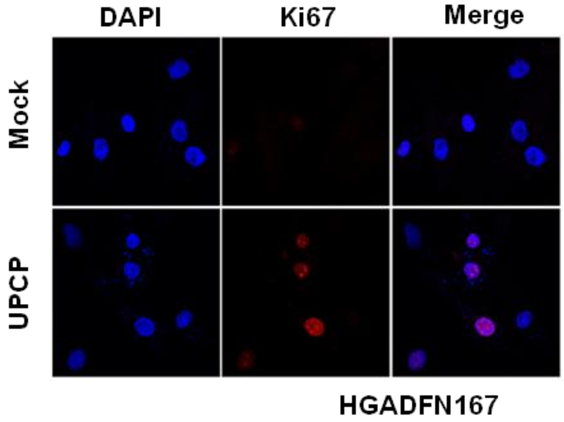

Extended Data Figure 6g. Images of Immunofluorescence.

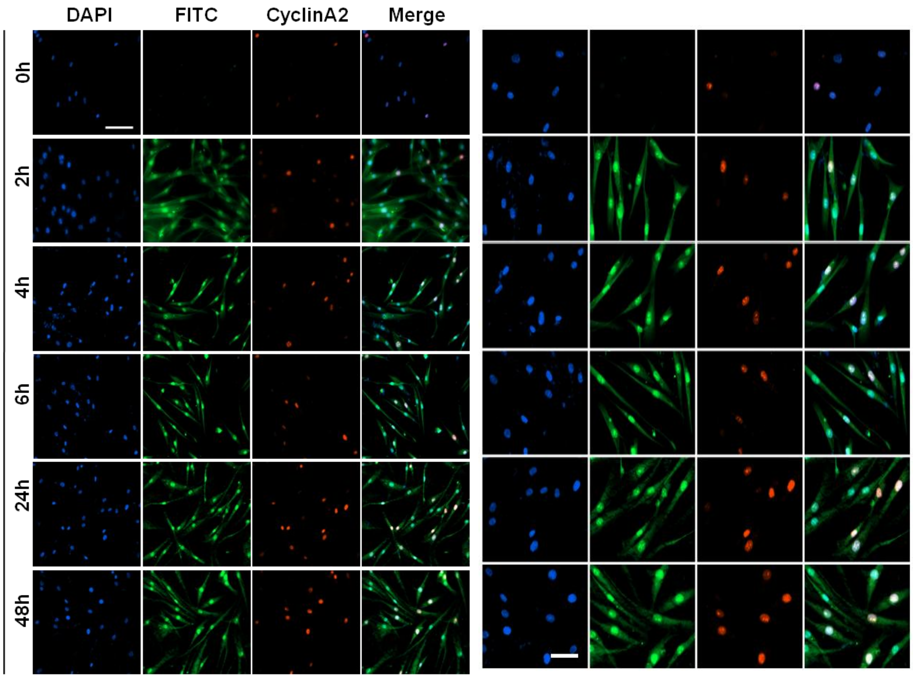

Extended Data Figure 6i. Images of Immunofluorescence.

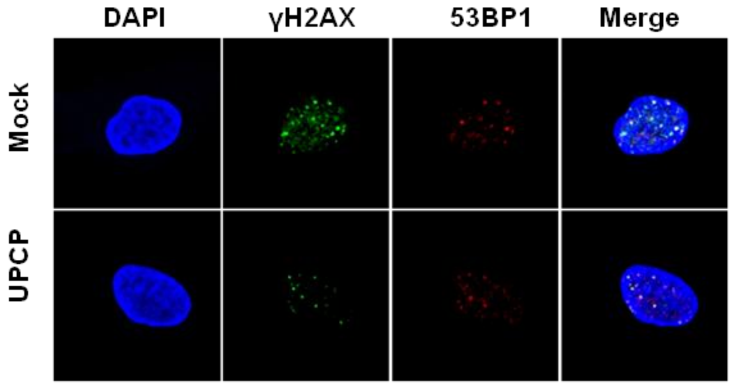

Extended Data Figure 6k. Images of Immunofluorescence.

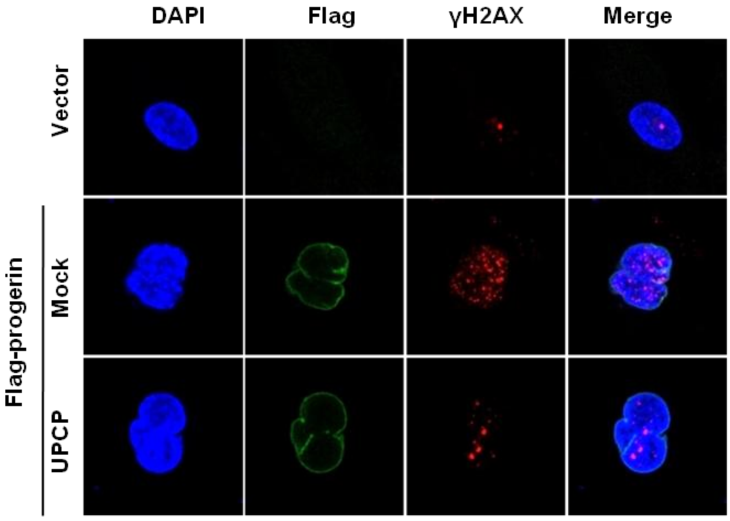

Extended Data Figure 6m. Images of Immunofluorescence.

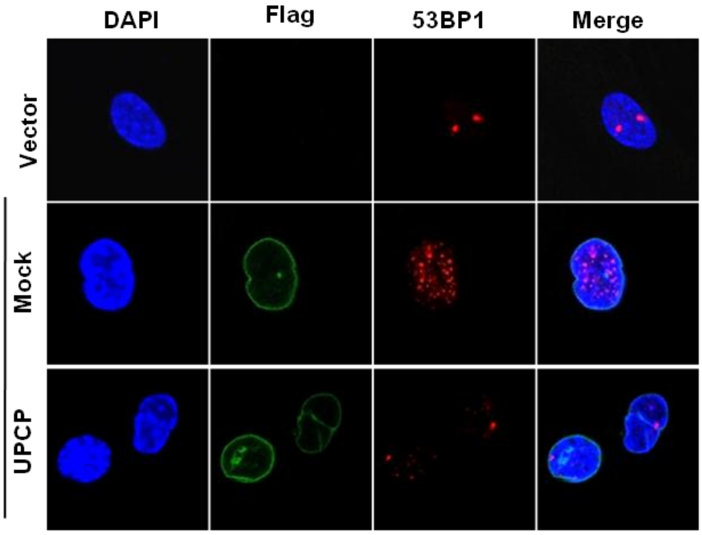

Supplement: Source Data Extended Data Fig. 6 — Unprocessed western blots and/or gels. [file 43587_2023_361_MOESM31_ESM.pdf]
