# Supplementary material for: Unique progerin C-terminal peptide ameliorates Hutchinson–Gilford progeria syndrome phenotype by rescuing BUBR1
Source: Nat Aging. 2023 Feb 2;3(2):185–201. doi: 10.1038/s43587-023-00361-w (PMC10154249; doi:10.1038/s43587-023-00361-w)

Extended Data Figure 7b. Images of β-Gal staining

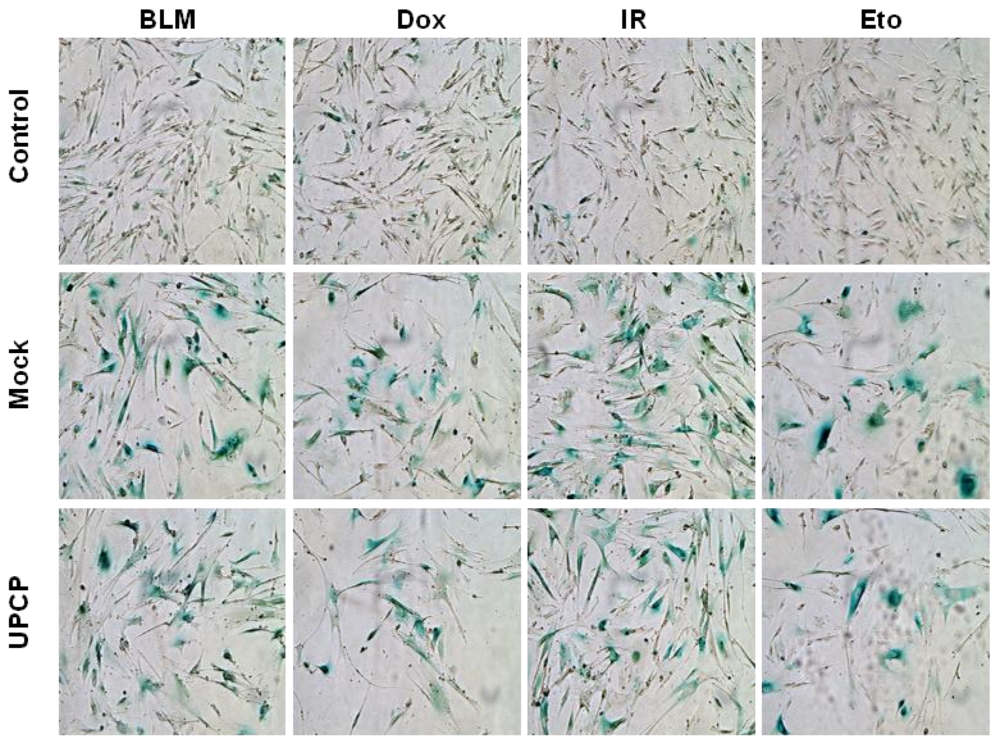

Extended Data Figure 7d. Full length images of immunoblots.

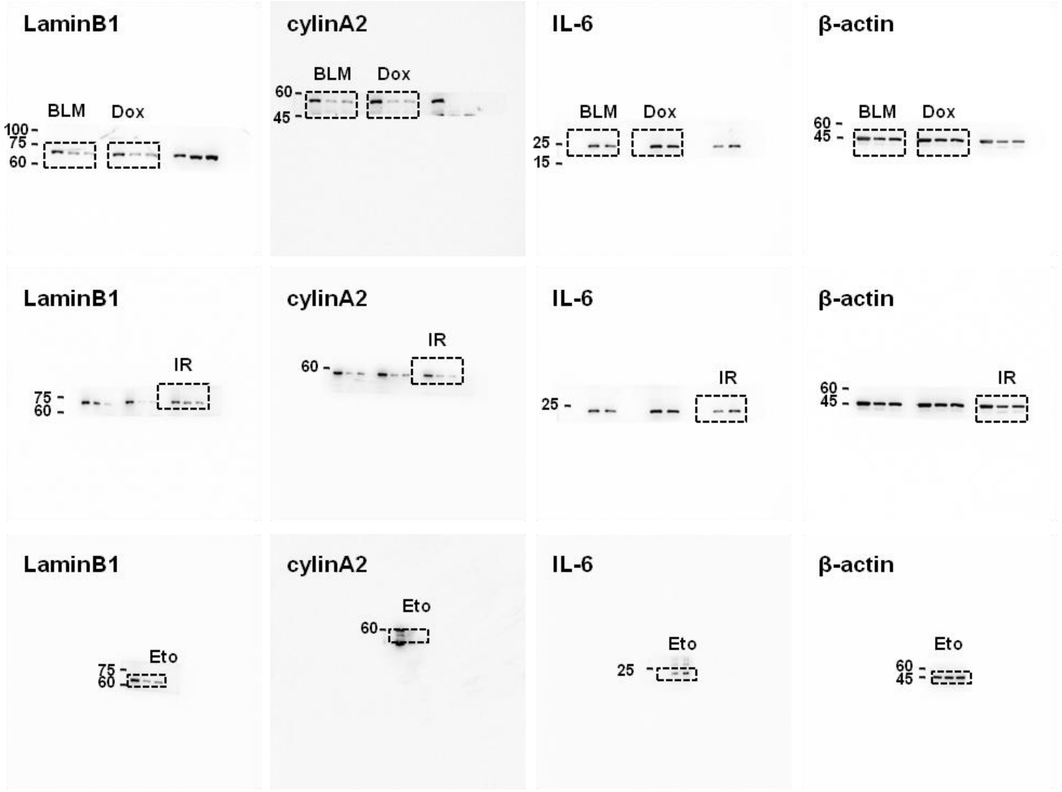

Extended Data Figure 7e. Images of Immunofluorescence.

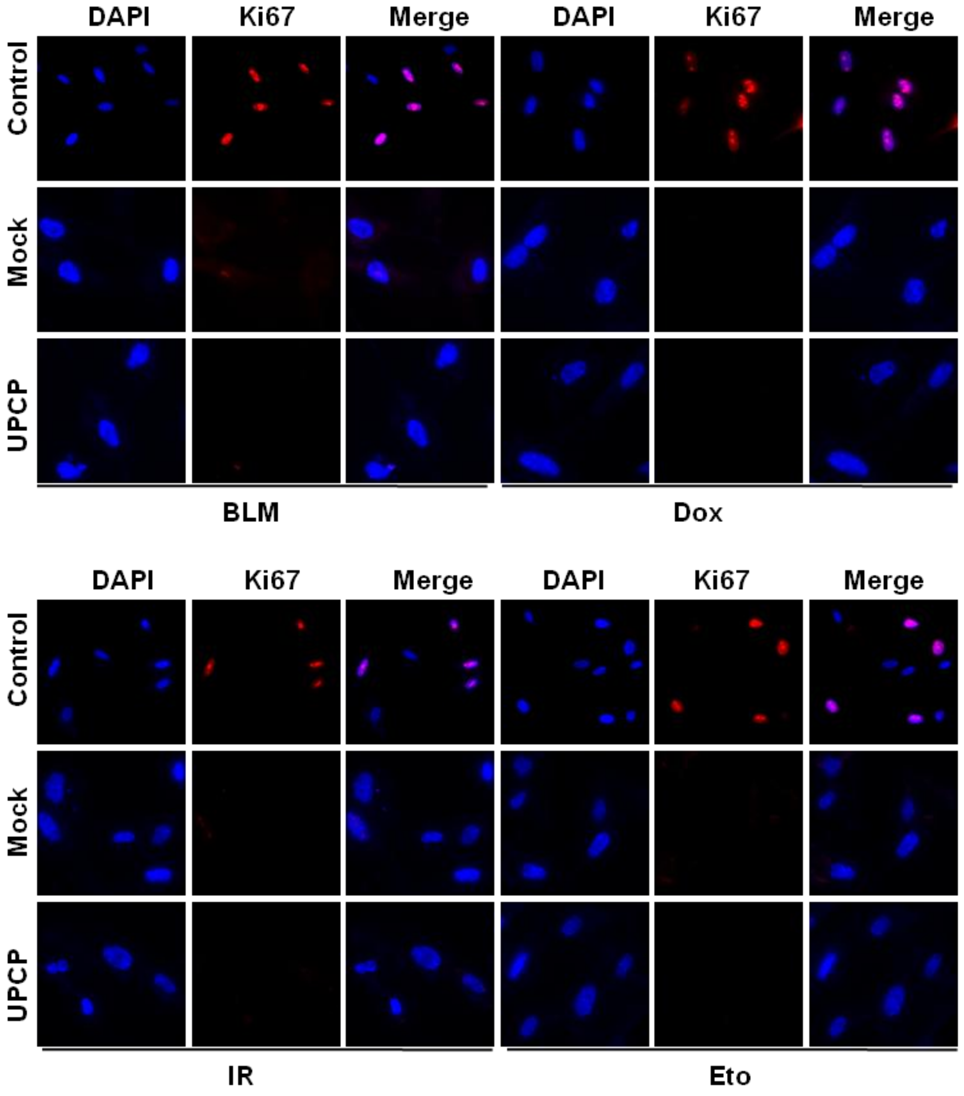

Supplement: Source Data Extended Data Fig. 7 — Unprocessed western blots and/or gels. [file 43587_2023_361_MOESM32_ESM.pdf]
