# Supplementary material for: Unique progerin C-terminal peptide ameliorates Hutchinson–Gilford progeria syndrome phenotype by rescuing BUBR1
Source: Nat Aging. 2023 Feb 2;3(2):185–201. doi: 10.1038/s43587-023-00361-w (PMC10154249; doi:10.1038/s43587-023-00361-w)

Extended Data Figure 8c. Images of 3 months *Lmna*$^{+/+}$ and *Lmna*$^{G609G/G609G}$ mice.

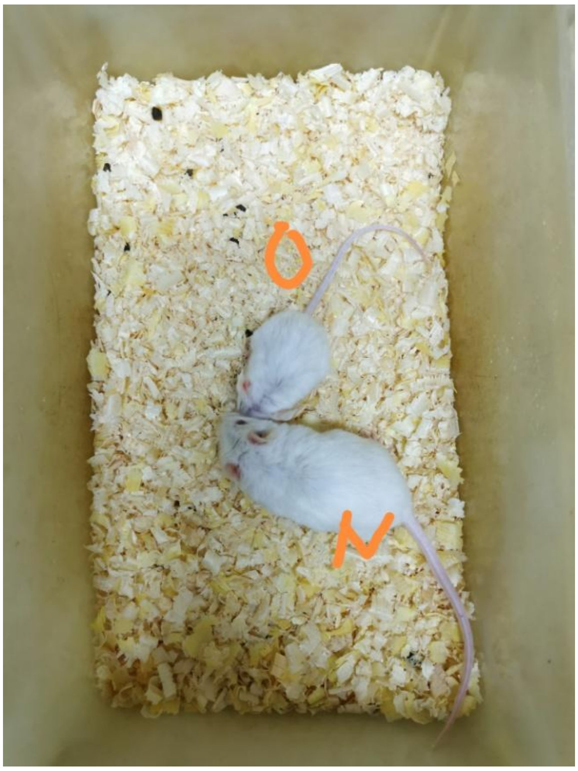

Extended Data Figure 8d. Full length images of immunoblots.

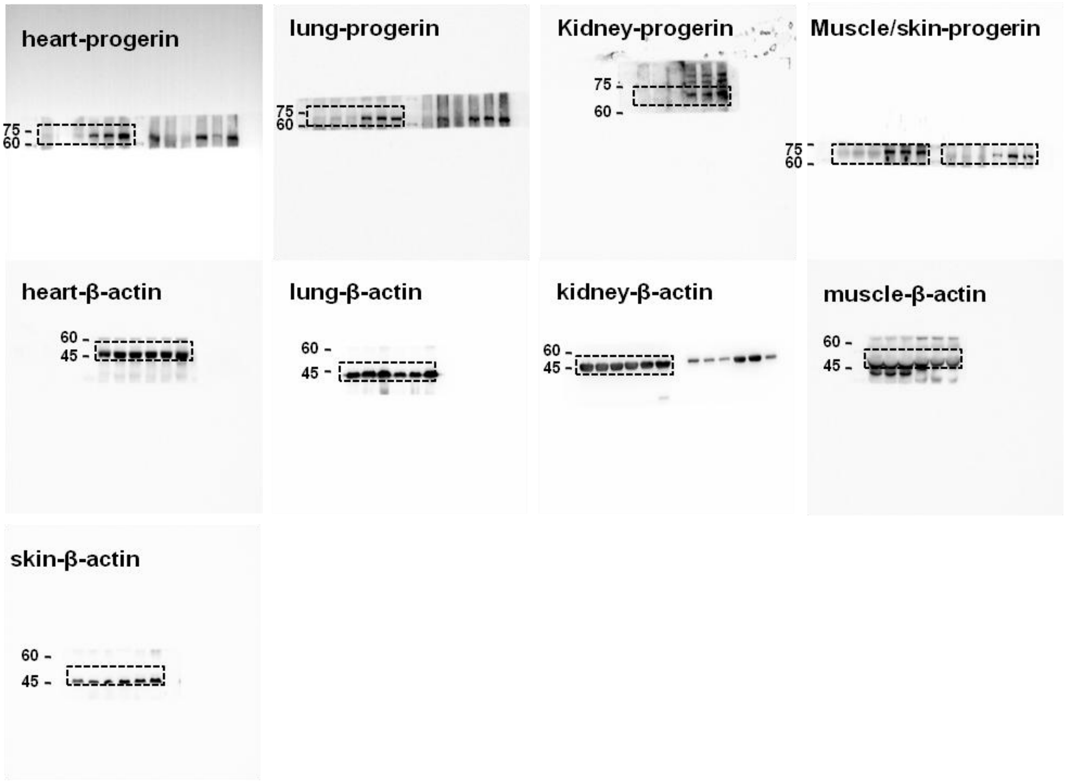

Extended Data Figure 8g. Images of IHC assay of LaminB1, p21 and IL-6 in skin of mice.

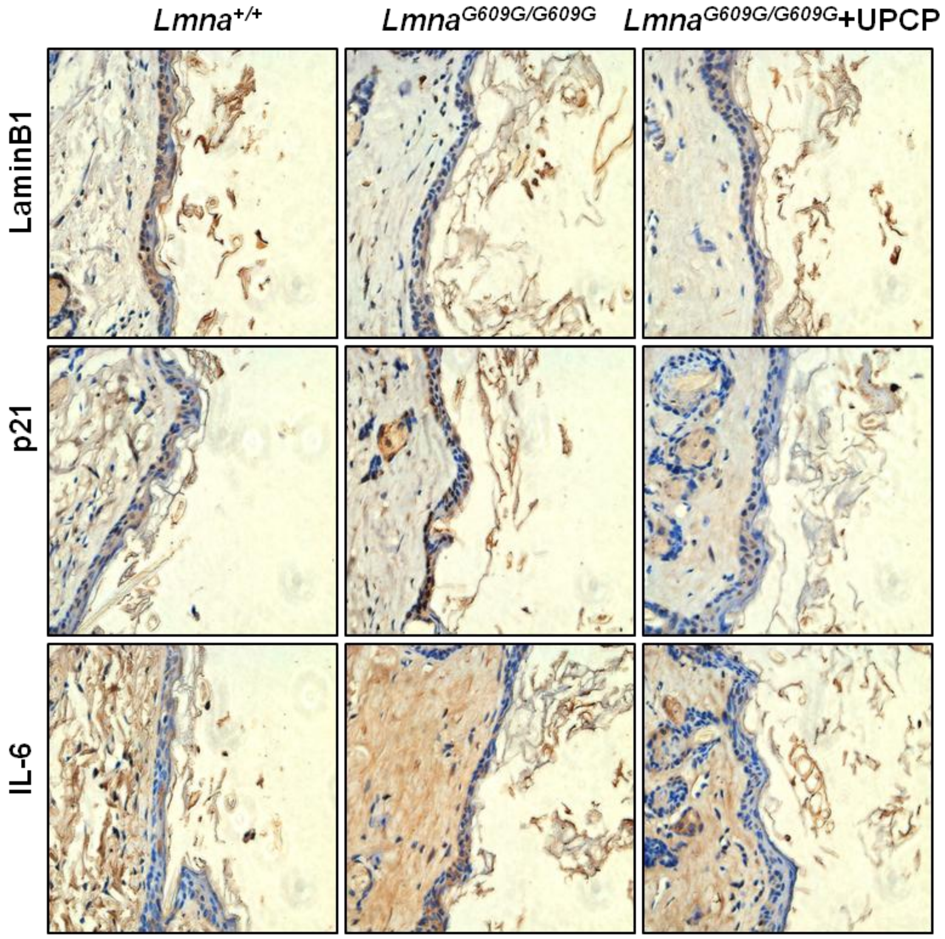

Supplement: Source Data Extended Data Fig. 8 — Unprocessed western blots and/or gels. [file 43587_2023_361_MOESM33_ESM.pdf]
