# Supplementary material for: Unique progerin C-terminal peptide ameliorates Hutchinson–Gilford progeria syndrome phenotype by rescuing BUBR1
Source: Nat Aging. 2023 Feb 2;3(2):185–201. doi: 10.1038/s43587-023-00361-w (PMC10154249; doi:10.1038/s43587-023-00361-w)

Extended Data Figure 9a,b. Full length images of immunoblots.

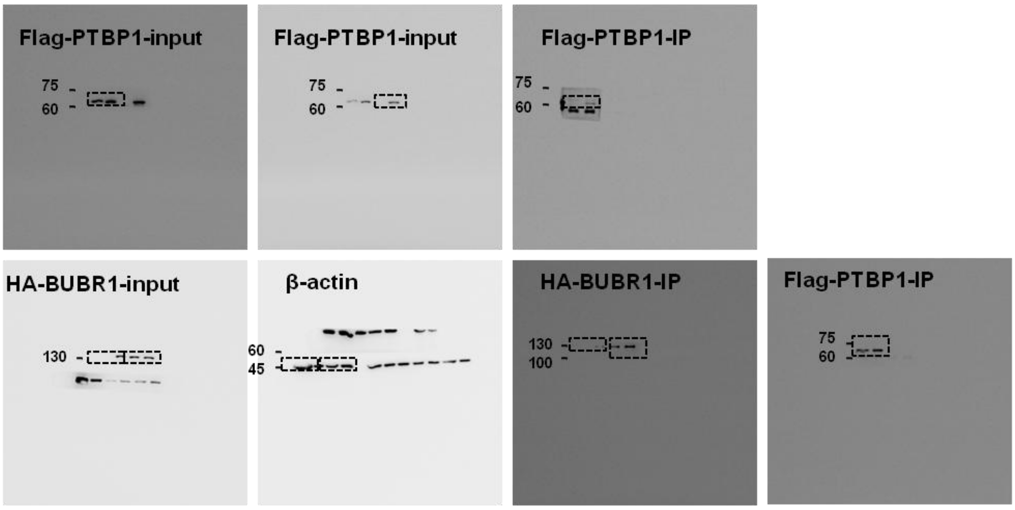

Extended Data Figure 9c. Full length images of immunoblots.

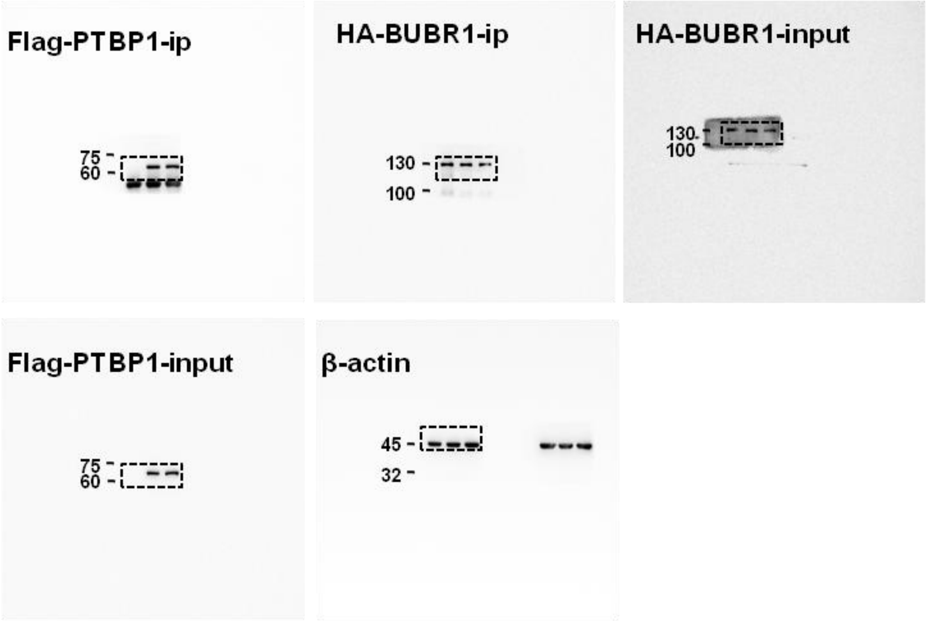

Extended Data Figure 9d. Full length images of immunoblots.

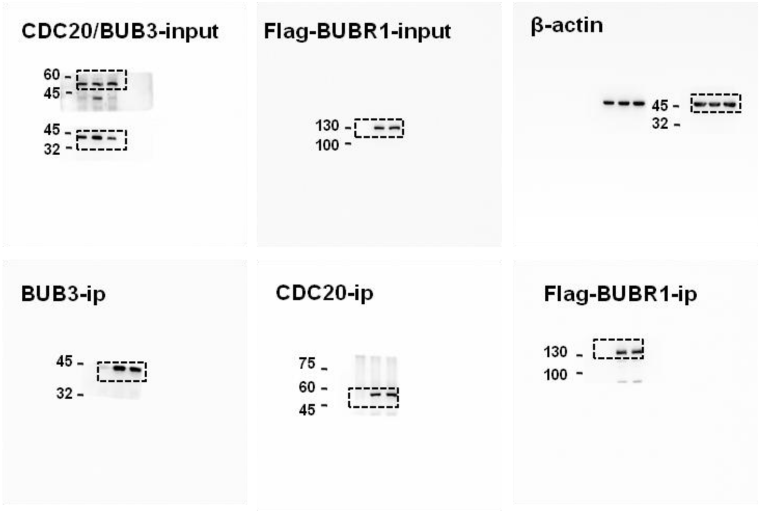

Supplement: Source Data Extended Data Fig. 9 — Unprocessed western blots and/or gels. [file 43587_2023_361_MOESM34_ESM.pdf]
